# Initiating olefin metathesis: alkylidenes from molecular Mo(iv)-oxo species, olefins and base-promoted proton transfer

**DOI:** 10.1039/d5sc06662j

**Published:** 2025-11-07

**Authors:** Darryl F. Nater, Felix J. de Zwart, Nicolas Kaeffer, Christophe Copéret

**Affiliations:** a Department of Chemistry and applied Biosciences, ETH Zürich Vladimir-Prelog-Weg 1-5 CH-8093 Zürich Switzerland ccoperet@ethz.ch; b Department of Electrosynthesis, Max Planck Institut for Chemical Energy Conversion Stiftstrasse 34-36 45470 Mülheim an der Ruhr Germany; c Université de Strasbourg, Université de Haute-Alsace, CNRS, LIMA, UMR 7042 67000 Strasbourg France

## Abstract

In olefin metathesis, metal alkylidenes and metallacyclobutanes are the two key intermediates of the Chauvin mechanism. In industrial metathesis catalysts based on supported group 6 metal oxides (*e.g.* MO_*x*_/SiO_2_), these intermediates, proposed to be in the +VI oxidation state, are postulated to be formed *in situ* from olefins and transient low-valent species in the +IV oxidation state. While recent studies have shown that molecularly-defined W(iv)-oxo species initiate olefin metathesis through C–H bond activation and proton transfer steps to generate metallacyclobutanes, less is known about the corresponding Mo-based systems. Here, we report the synthesis of a pyridine-stabilized Mo(iv)-oxo compound, [MoO(OC(CF_3_)_3_)_2_py_3_], and show that this compound also initiates olefin metathesis, once activated with B(C_6_F_5_)_3_. This activation is evaluated to have a low efficiency (≈0.2%) and illustrates the difficulty in generating active species from low valent sites, thus explaining the relatively low activity of classical heterogeneous catalysts. DFT calculations support that the initiation steps differ from W: Mo enters the catalytic cycles from the metal alkylidenes rather than metallacyclobutanes for W, while still involving C–H activation and base-assisted proton transfers as common elementary steps. Initiation through proton transfer better explains the role of promoters in the corresponding heterogeneous catalysts.

## Abstract 2

A molecular Mo(iv)-oxo complex initiates olefin metathesis *via* a base-assisted proton transfer to form Mo-alkylidenes, pointed out possible activation pathways in the corresponding heterogeneous metathesis catalysts.

## Graphical abstract

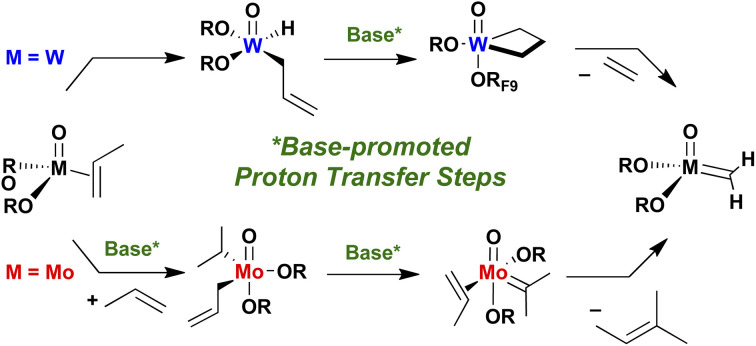

## Introduction

**Fig. 1:**
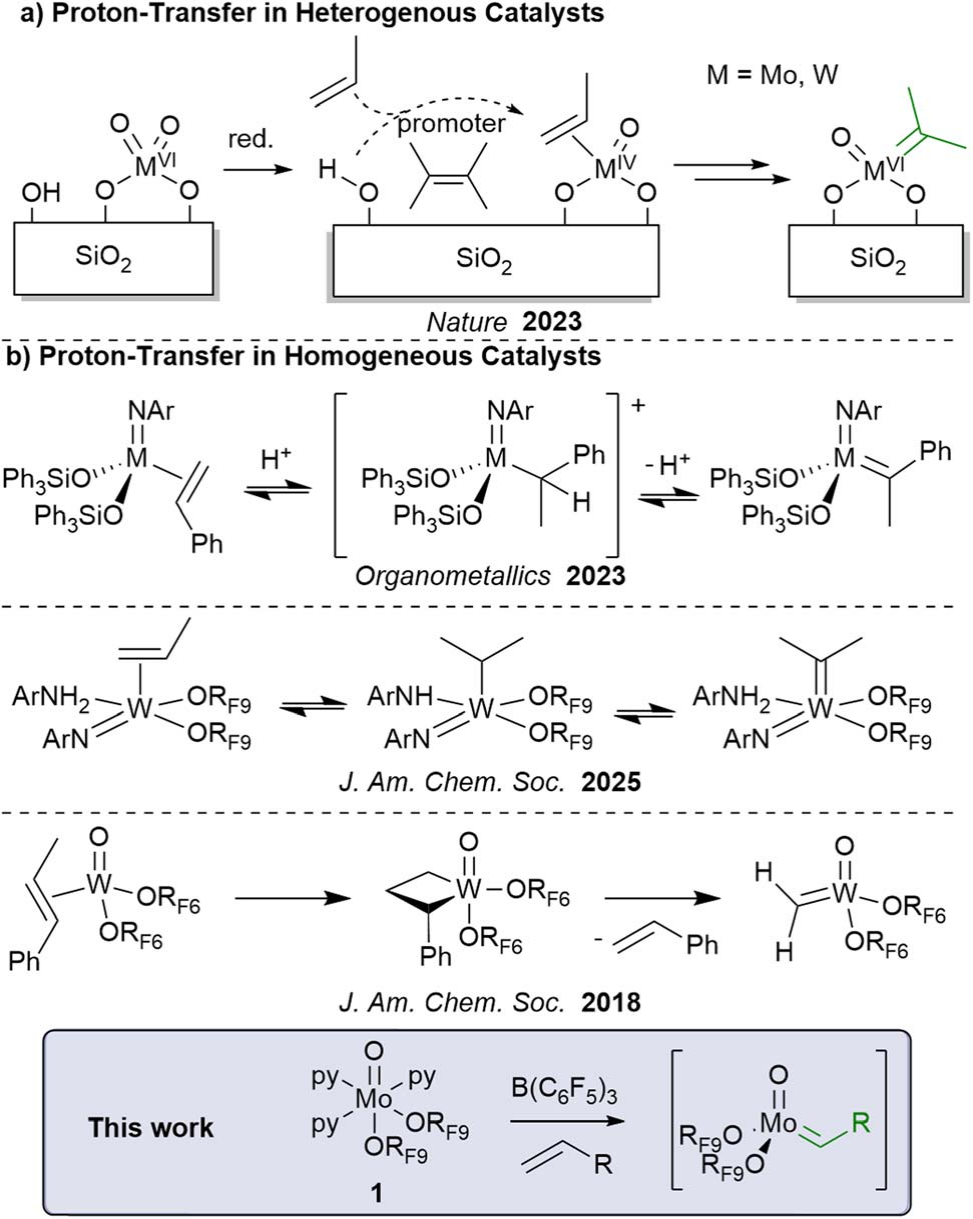
Formation of alkylidene active species in (a) heterogeneous and (b) homogeneous catalytic systems. OR_F6_=OC(CF_3_)_2_CH_3_, OR_F9_=OC(CF_3_)_3_.

Olefin metathesis is a highly atom-economical C=C bond forming reaction. As such, this transformation has received much attention, with industrial applications across scales, from petrochemicals to polymers and fine chemicals.^1^ Among olefin metathesis catalysts, those based on Mo and W stand out for being used in both homogeneous and heterogeneous processes, whether as well-defined Schrock high-valent alkylidenes,^2^ ill-defined molecular systems or supported metal oxides, *e.g.* MoO_3_/SiO_2_–Al_2_O_3_ and WO_3_/SiO_2_.^3^ Notably, supported Mo-based catalysts operate at lower temperatures and have been used in the Shell Higher Olefin Process (SHOP) to produce long chain α-olefins.^4^ Prepared by impregnation and calcination, the supported MoO_*x*_ catalysts contain high-valent Mo(vi) oxo species, from which alkylidenes and metallacyclobutanes, the reaction intermediates of the Chauvin mechanism, are formed under reaction conditions, *i.e.* a stream of olefins at high temperatures (typically >250 °C).^5^ Reduction of the metal sites and proton-transfer chemistry are both invoked for the generation of metathesis-active species. Heterogeneous processes have been proposed to involve the reduction of Mo(vi) to Mo(iv) species, which are then converted *in situ* into the Chauvin intermediates.^6,7^ Reinforcing this concept, chemically reduced silica-supported Mo(vi) or W(vi) oxo species initiate olefin metathesis at 70 °C, thanks to the formation of highly reactive M(iv) species that generate the metathesis-active species upon contact with olefins.^8,9^ In addition, recent works have shown that the number of active sites for silica-supported MO_*x*_-based catalysts can be increased upon addition of higher olefins in the gas feed, which act as proton shuttle reagents to help form the active species (Fig. 1a).^10,11^ In various molecular systems, proton transfer has also been recently shown to play a role in forming the metathesis intermediates from olefins (Fig. 1b).^12–19^

Overall, the roles of low-valent species and proton transfer in olefin metathesis have emerged in recent years, calling for a better understanding of the initiation process, using model systems.^20–22^ Here, we develop and fully characterize a Mo(iv)-oxo molecular compound bearing nonafluoro-*tert*-butoxide ligands (^*t*^Bu_F9_O) and stabilized by pyridine units (1), paralleling our earlier work on the corresponding W-based systems.^13^ We show in particular that such species are competent enough to initiate metathesis under mild conditions upon activation with B(C_6_F_5_)_3_ (Fig. 1). Detailed experimental and computational studies indicate that initiation takes place *via* base-assisted proton-transfer steps coupled with the oxidation of the metal center to generate the necessary alkylidenes. That mechanism is distinct from the W analogue that prefers to enter the catalytic cycle from the metallacyclobutane intermediates.

## Results

**Fig. 2:**
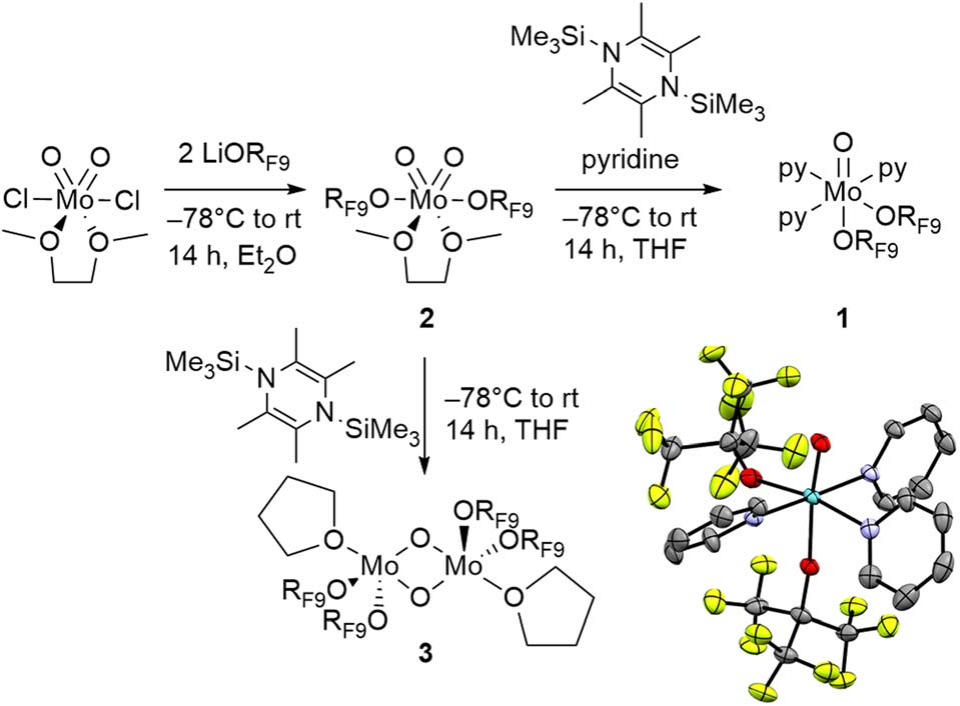
Synthesis of 1, 2 and 3; crystal structure of 1 shown as thermal ellipsoid plots at 50% probability with the hydrogen atoms omitted.

The Mo(iv) oxo compound 1 is prepared in two steps from [MoO_2_Cl_2_(dme)] (Fig. 2). Salt metathesis of [MoO_2_Cl_2_(dme)] with 2 equiv. of LiOR_F9_ (OR_F9_=OC(CF_3_)_3_) at low temperature provides [MoO_2_(OR_F9_)_2_(dme)] (2) in 74% yield after crystallization from Et_2_O at −30 °C (long white needles suitable for X-ray diffraction studies). The subsequent reaction of 2 with 1.1 equiv. of 2,3,5,6-tetramethyl-1,4-bis(trimethylsilyl)-1,4-diaza-2,5-cyclo-hexadiene (BTDC) at −78 °C in the presence of excess pyridine (Py) leads to the reduced compound [Mo^IV^O(OR_F9_)_2_Py_3_] 1 in 52% yield after crystallization in a toluene/pentane solution (black crystalline solid suitable for X-ray diffraction studies). We note that the use of less electron withdrawing alkoxide ligands (OR_F*i*_ with *i* = 6, 3 or 0) did not so far yield the analogues of 1 under a broad range of explored conditions and that attempts to produce low-valent derivatives of 1 using less strongly coordinating L ligands, such as THF and DME, invariably resulted in the formation of dimeric or oligomeric species such as 3.

Notably, the dioxo Mo(vi) compound 2 adopts a distorted octahedral geometry, where the alkoxides are positioned *trans* to each other with an R_F9_O–Mo–OR_F9_ angle of 152.02° and the *cis*-oxo ligands show an O=Mo=O angle of 104.12°. The Mo–O distances for the alkoxides and the oxo are 1.96 Å and 1.68 Å, respectively, in close accordance with similar reported compounds.^23,24^ Regarding 1, its XRD determined structure shows a slightly distorted octahedral geometry, where the alkoxide ligands are now *cis* to each other, with one in the axial position, *trans* to the oxo (RO–Mo=O angle of 171.01°) and the other one in an equatorial position with an RO–Mo=O angle of 101.49°. This structure of 1 is analogous to W compounds of the same form [WO(OR_F9_)_2_Py_3_].^13^ We also observe a significant elongation of the Mo–O bonds compared to 2, with Mo–OR_F9_ and Mo=O reaching 2.02 Å (equatorial OR_F9_), 1.98 Å (axial OR_F9_) and 1.77 Å, respectively.

With compound 1 in hand, we next explored its reactivity towards olefin metathesis using prototypical substrates.^25^ Contacting 1 with 1-nonene did not result in any metathesis activity, neither at 30 °C nor at 70 °C. This lack of reactivity is not surprising and can be attributed to saturation of the coordination sphere by pyridine, which hinders olefin coordination and further reaction. Addition of B(C_6_F_5_)_3_ as a pyridine scavenger to the reaction mixture results in metathesis activity at 70 °C (Table 1). The highest activity can be achieved by using 3 to 4 equiv. (≥1 equiv. with regards to pyridine) of B(C_6_F_5_)_3_ at 70 °C, while using 2 equiv. only provides low activity. The use of ZnCl_2_ instead of B(C_6_F_5_)_3_ results only in minute conversion (0.5%) after 24 h, indicating that a well-soluble Lewis acid like B(C_6_F_5_)_3_ is advantageous. Finally, one should note that small amounts of olefin isomerization products (∼2%) are also observed under these reaction conditions. In addition, we point out that, in contrast to its W analogue, contacting 1 with olefins (ethylene or propylene) does not result in the exchange of one pyridine with one olefin.^13^ Taken together, this data supports the need to generate highly active low coordinated species by removal of pyridine through the formation of py-B(C_6_F_5_)_3_ Lewis adducts.

**Table 1:** Catalytic activity of complex 1 with 300 equivalents of substrate in toluene

Substrate	B(C_6_F_5_)_3_/equiv. per Mo	Temperature (°C)	Conversion@ 24 h
1-Nonene	0	70	0.0%
1-Nonene	2	70	0.7%
1-Nonene	3	70	34.0%
1-Nonene	4	70	58.2%
4-Nonene	3	70	18.5%
Styrene	3	70	1.4%
Allylbenzene	3	70	37.0%
β-Methylstyrene	3	70	8.4%

Next, the amount of active species generated *in situ* is evaluated by comparing the metathesis activity of 1*vs.* that of the well-defined alkylidene equivalent [(R_F9_O)_2_MoO(=CHR)]. While the former reaches a TOF of 0.5 min^−1^ in 1-nonene metathesis, the latter has a reported TOF of 210 min^−1^ under the same conditions.^26^ Comparing these values indicates that only a small fraction of active species is generated from 1, *i.e.* on the order of *ca.* 0.2%.

With internal olefins like 4-nonene, we observe a long induction period and little activity with conversions after 24 h limited to 0.3% and 18.5% at 30 °C and 70 °C, respectively. The maximum observed TOF (0.05 min^−1^) is again much lower than that observed with the corresponding well-defined alkylidene (180 min^−1^), thus indicating the formation of even smaller amounts of active species (≪0.1%). Notably, allylbenzene and β-methylstyrene also undergo metathesis with 1, while styrene only shows a very limited conversion (1.4% after 24 h at 70 °C), probably indicating the importance of allylic protons for more efficient activation, paralleling observations with the W-analogue.^13^ Comparing allylbenzene to β-methylstyrene, the terminal olefin shows higher activity, similar to what was observed for 1-nonene and 4-nonene. We note again evidence of olefin isomerization in these cases.

**Fig. 3:**
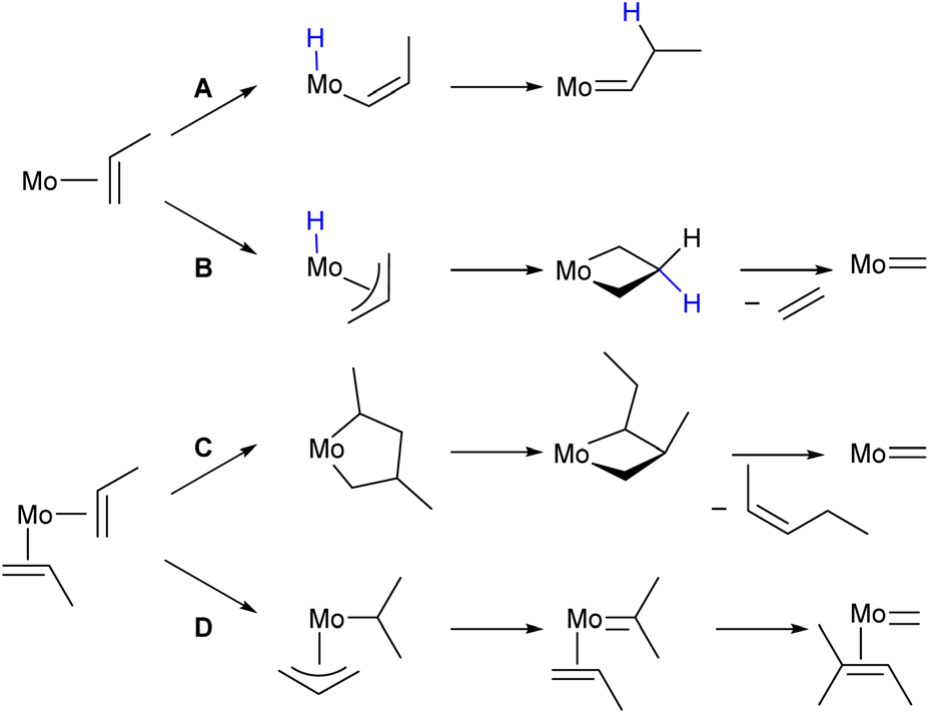
Metathesis initiation mechanisms under consideration for monomeric molybdenum(iv) species: (A) vinylic and (B) allylic C–H activation, (C) ring contraction and (D) alkyl–allyl mechanism.

Having established the catalytic capabilities of 1, we next investigate the initiation mechanism leading to the metathesis active species by DFT calculations, using propene as the simplest model substrate. Classical pathways can include: (A) allylic or (B) vinylic C–H activation, (C) metallacyclopentane (MCP) ring contraction or (D) a less common alkyl–allyl pathway (Fig. 3).^26–32^ We first explore the formation of olefin complexes by removing all pyridine ligands upon reaction with B(C_6_F_5_)_3_, a Lewis acid, and coordinating one olefin (Scheme S1). De-coordination of all pyridine ligands (*via* formation of py-B(C_6_F_5_)_3_ adducts) and coordination of propene leads to formation of olefin complex IM0. This overall reaction is energetically favoured, with a Δ*G* of −7.7 kcal mol^−1^, mirroring the previous study on W where a similar process is also found to be favoured (Δ*G* = −14.8 kcal mol^−1^).^13^ In view of the isolation of the dimeric compound 3 upon reduction of 2, we also explore the possibility of formation of a [Mo(O)(py)(OR_F9_)_2_]_2_ dimer. The overall reaction is only slightly endergonic from IM0 (Δ*G* = +5.0 kcal mol^−1^); considering the reaction conditions for the formation of 3 and metathesis, such a byproduct is likely accessible, consistent with the low efficiency of activation and the low amount of active sites generated upon activation.

**Fig. 4:**
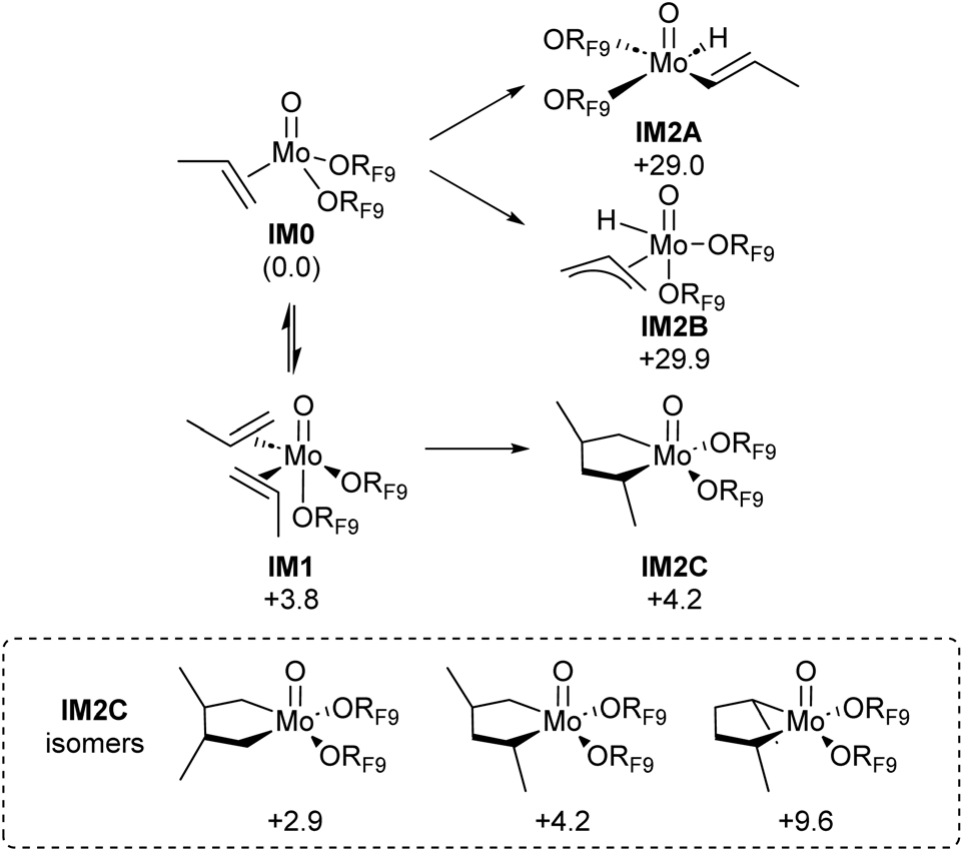
Computed Gibbs free energies (Δ*G*, kcal mol^−1^) of the first intermediates generated through vinylic and allylic C–H activation, or oxidative coupling yielding metallacyclopentanes. Calculated at the B3LYP-D3(BJ)/def2-TZVP level of theory. Gibbs free energies in kcal mol^−1^ relative to intermediate IM0.

As there is no strong evidence either for or against any of the initiation pathways proposed in the literature (*vide supra*), we investigate further the initiation steps for all three mechanisms, using the olefin complexes IM0 and IM1 as an entry point (Fig. 4). The vinylic or allylic C–H activation pathways from IM0 are prohibitively high in energy with Δ*G* = +29.0 kcal mol^−1^ for IM2A (pathway A) and Δ*G* = +29.9 kcal mol^−1^ for IM2B (pathway B), which is notably disfavoured by comparison with W.^13^

The four-coordinate olefin complex IM0 can accommodate another equivalent of olefin to form olefin complex IM1. We note that DFT calculated translational entropies of propene are slightly overestimated compared to experimentally determined solvation entropies.^33–35^ Therefore, to provide a fair comparison of mechanisms including additional propene, the Gibbs free energy for steps involving propene binding is calculated by including only two thirds of the entropy contribution in that step.^36,37^ This provides a Gibbs free energy of +3.8 kcal mol^−1^ for IM1. With 2 equiv. of propene coordinated in IM1, the formation of metallacyclopentane (pathway C) intermediate IM2C from this olefin complex can be very facile, depending on the position of the two (methyl) substituents (in *cis* or *trans* configurations, following a head-to-head, head-to-tail or tail-to-tail coupling) and is associated with low energy barriers (Scheme S2, TS1C). The most stable MCP bears the two substituents in the β-position (Δ*G* = 2.9 kcal mol^−1^), while the most unstable ones have the two substituents in the α-position (Δ*G* = +9.6 kcal mol^−1^). The α,β-substituted structures typically have intermediate formation energies, with *trans* diastereomers being lower in energy than their *cis* counterparts (Δ*G* = +4.2 kcal mol^−1^) (Fig. 4).

**Fig. 5:**
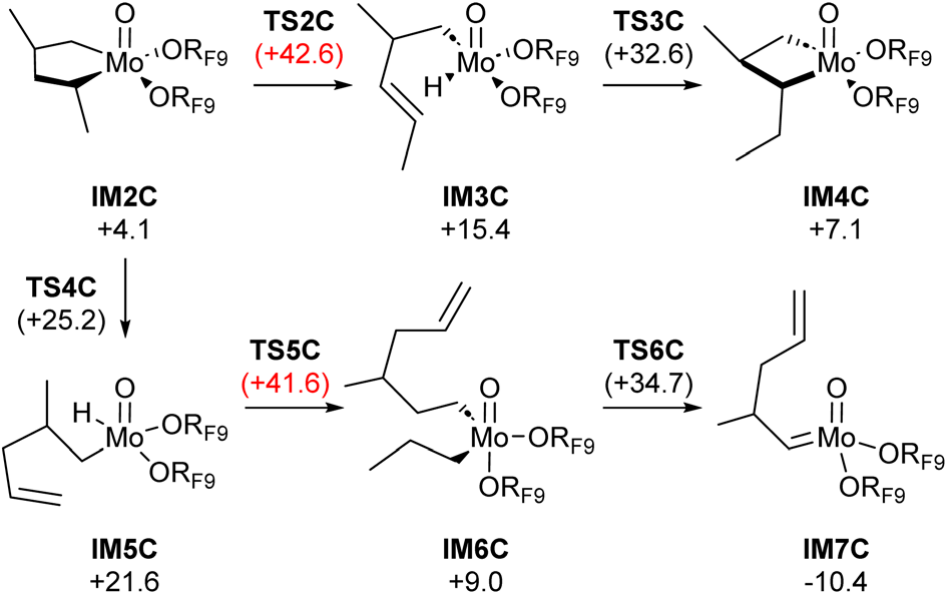
Activation through molybdenum hydride intermediates from metallacyclopentane: minimum-energy reaction pathway calculated at the B3LYP-D3(BJ)/def2-TZVP level of theory. Gibbs free energies in kcal mol^−1^ relative to IM0.

We next study the conversion of metallacyclopentanes into the metallacyclobutane olefin metathesis intermediate, *via* ring contraction, involving β-H transfer and insertion steps (Fig. 5).^27^ From intermediate IM2C, β-H transfer can involve an exocyclic or endocyclic proton. For the endocyclic proton, β-H transfer providing intermediate IM3C is prohibitively high with a barrier of Δ*G*^‡^ = +42.6 kcal per mol (TS2C). In contrast, α-substituted metallacyclopentanes can also undergo β-H transfer from an exocyclic methyl substituent (TS4C). This step is significantly more accessible, with an energy barrier of Δ*G*^‡^ = +25.2 kcal mol^−1^. That result aligns with previous investigations into isomerization over Mo(iv) complexes.^30^ The accessibility of intermediate IM5C is presumably responsible for the isomerization activity of this catalytic system. However, the conversion of IM5C into a metathesis active intermediate, involving a third propene molecule, is too energy demanding with a considerably high energy barrier of Δ*G*^‡^ = +41.6 kcal mol^−1^, arising from the large entropy inflicted by the association of the third equivalent of propene required for productive catalysis (see Scheme S3 for enthalpies). Overall, while the formation of the metallacyclopentane intermediates can explain the observed isomerization activity, no reasonable pathway can be found to generate metathesis intermediates through the classical proposed pathways (Fig. 3A–C).

**Fig. 6:**
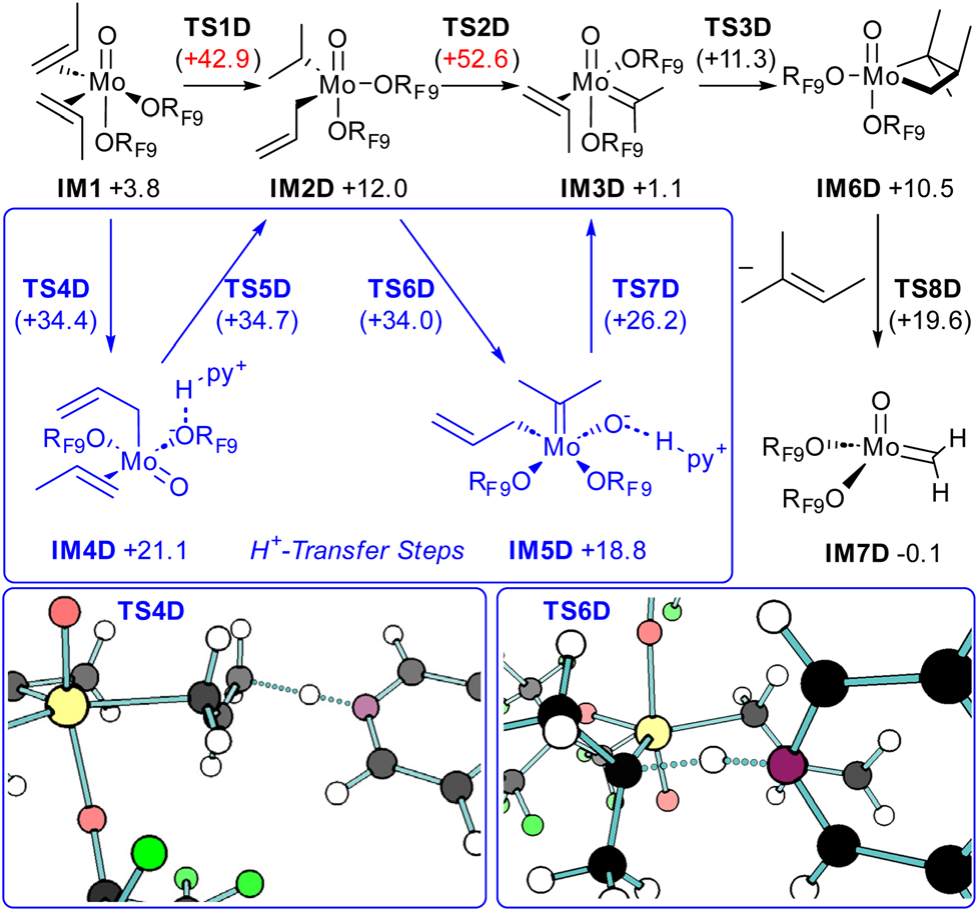
Activation through allyl–alkyl mechanism: minimum-energy reaction pathway, calculated at the B3LYP-D3(BJ)/def2-TZVP level of theory. Gibbs free energies in kcal mol^−1^ relative to intermediate IM0.

Having exhausted the unassisted pathways that involve C–H activation over the Mo centre, we set out to calculate the recently proposed alkyl–allyl pathway D (Fig. 6).^31,32^ Direct activation of one propylene ligand by another leads to isopropyl–allyl intermediate IM2D, with a prohibitively high barrier of +42.9 kcal mol^−1^. From this dialkyl Mo(vi) intermediate, α-H transfer from the isopropyl to the allyl comes with an even higher barrier of +52.6 kcal mol^−1^, but directly forms metathesis active intermediate IM3D (see Scheme S4 for enthalpies).

While these barriers are clearly unsurmountable, the presence of pyridine significantly lowers energy barriers, thus providing additional base-assisted reaction pathways. From IM1, deprotonation of one propylene ligand through transition state TS4D, with a barrier of +34.4 kcal mol^−1^, provides intermediate IM4D, which in the lowest energy conformation contains a hydrogen bond between the alkoxide moiety and the pyridinium ion. The oxo-moiety of the molybdenum complex plays an active role in stabilizing the charged intermediates but is not protonated itself as is the case with imido complexes.^21^ The subsequent reprotonation is coupled with the oxidation of the metal center and affords the Mo(vi) intermediate IM2D through transition state TS5D. Another deprotonation of the isopropyl ligand can now occur through transition state TS6D, with a barrier of +34.0 kcal mol^−1^, providing intermediate IM5D. A last protonation finally provides the metathesis active carbene complex IM3D, which readily interconverts to TBP complex IM6D, and terminal carbene IM7D. Involvement of additional B(C_6_F_5_)_3_ has negligible impact on the transition state energies (Scheme S5). In this mechanism, the rate determining step is the protonation of the olefin complex to form an allyl type species with a barrier of +34.7 kcal mol^−1^. This barrier is sufficient to explain the low initiation efficiency, and is in line with the estimated 0.2% of the catalyst forming active species under the reaction conditions. Therefore, the limited amount of active catalyst can be attributed to the slow initiation step in competition with other pathways.

## Conclusions

**Fig. 7:**
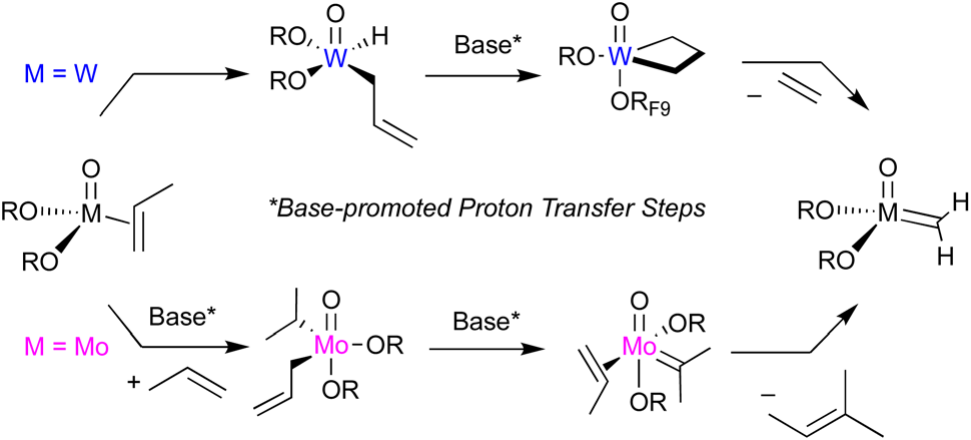
Base-promoted initiation of M(iv) olefin metathesis precatalysts: Mo *vs.* W.

A well-defined, low-valent Mo(iv) oxo-complex, stabilized by pyridine ligands, has been shown to initiate olefin metathesis upon activation with B(C_6_F_5_)_3_, paralleling what has been observed for the corresponding W analogues. However, Mo displays a distinct reactivity from W (Fig. 7). First, Mo has a greater propensity than W for post-olefin isomerization, and in addition, no stable olefin adduct can be observed upon exchange with the pyridine ligand. Computational studies support that the direct activation of a C–H bond on Mo(IV) sites (freed from pyridine), in particular the allylic ones yielding a π-allyl hydride, is disfavoured, sharply diverging from the situation with W. The formation of metallacyclopentane intermediates *via* oxidative coupling followed by ring contraction is also shown to be disfavoured. Yet, this pathway leads to accessible Mo–H *via* β-H transfer on an exocyclic alkyl substituent of metallacyclopentane intermediates, explaining the observed post-isomerization. Instead, Mo prefers a Lewis-base (here pyridine) assisted oxidation-coupled proton transfer from the bis-olefin complex, yielding an alkyl–allyl species, which can generate the necessary alkylidene intermediate *via* α-H abstraction. This process is associated with a sizeable energy barrier and competes with other non-productive pathways, thus explaining the low amount of active sites generated from Mo(iv) species. Overall, this study highlights similitudes and differences between Mo and W, both sharing oxidation, C–H activation and proton transfer as key steps to generate the Chauvin intermediates. However, while the direct activation of an allylic C–H bond of a coordinated olefin enables W to generate a metallacyclobutane intermediate *via* base-assisted proton transfer, Mo prefers base-assisted proton transfer from the bis-olefin complex, yielding an alkyl–allyl complex that can enter the Chauvin cycle *via* an alkylidene intermediate upon α-H abstraction. The additional evidence for base-assisted proton-transfer pathways, here for a molecularly-defined system, further supports what has been proposed on supported Mo- and W-based olefin metathesis catalysts, where bulky olefins, unamenable to metathesis, can facilitate proton transfer and hence increase the number of active sites.^10,11^

## Author contributions

D. F. N. performed the synthesis, analysis and catalytic testing. F. d. Z. performed the DFT calculations. All authors contributed to the planning, data analysis and writing of the manuscript.

## Conflicts of interest

There are no conflicts of interest to declare.

## Supplementary Material

SC-OLF-D5SC06662J-s001

SC-OLF-D5SC06662J-s002

## Data Availability

CCDC 2117645 and 2482767 contain the supplementary crystallographic data for this paper.^38*a*,*b*^ The data supporting this article have been included as part of the supplementary information (SI). Supplementary information: synthetic details, catalytic data and computational coordinates. See DOI: https://doi.org/10.1039/d5sc06662j.
