# Supplementary material for: Initiating olefin metathesis: alkylidenes from molecular Mo(iv)-oxo species, olefins and base-promoted proton transfer
Source: Chem Sci. 2025 Nov 7;16(48):23351–6. doi: 10.1039/d5sc06662j (PMC12593810; doi:10.1039/d5sc06662j)
Supplement: SC-016-D5SC06662J-s001 [file SC-016-D5SC06662J-s001.pdf]

Supplementary Information (SI) for Chemical Science.
This journal is © The Royal Society of Chemistry 2025

Initiating Olefin Metathesis: Alkylidenes from Molecular Mo(IV)–Oxo Species, Olefins and Base-Promoted Proton Transfer

Darryl F. Nater, Felix J. de Zwart, Nicolas Kaeffer and Christophe Copéret*

Table of Contents

Table of Contents 2
Experimental Procedures 3
General Methods 3
Synthetic Procedures 3
Catalytic Procedures 4
Computational Details 4
Crystallographic data 5
Catalytic Details 7
NMR Spectra of compounds 9
Presence of py·B(C_6F_5)$_3$ Adduct 11
Computational Results 12
Influence of substitution pattern on the energies of metallacyclopentane species 12
Potential energy surfaces of Pathway C 13
Pathway D, unassisted 13
Coordinates of calculated structures 16
References 35

Experimental Procedures

General Methods

All experiments were performed under dry and oxygen free argon atmosphere, either in a glovebox or using standard Schlenk techniques. Pentane, diethyl ether and toluene were purified using an MBRAUN SPS system equipped with double alumina columns and degassed by 3 freeze-pump-thaw cycles before use. C_6D_6 and THF were distilled from Na/benzophenone. Molybdenum-dioxo-dichloride was purchased from Sigma Aldrich. 2,3,5,6-tetramethyl-1,4-bis(trimetylsilyl)-1,4-diaza-2,5-cyclohexadiene and MoO_2Cl_2(dme) and $MoO_2(OC(CF_3)_3)_2$(dme) **2** were synthesized according to literature procedures.[1,2,3] Pyridine was distilled from Na and filtered through a plug of alumina directly before use. Perfluoro tert-butanol (HO^tBuF_9) was purchased from Fluorochem. LiO^tBu_{F9} was prepared by treating the corresponding alcohol with *n*BuLi in diethyl ether at 0°C; the alkoxide salt was sublimed in *vacuo* at 60°C before use. Tris(pentafluorophenyl)borane was purchased from TCI and sublimed before use. 1- and 4-Nonenes were purchased from TCI and distilled from Na. Styrene, β-Methylstyrene and Allylbenzene was purchased from ABCR and distilled from CaH_2. All olefins were treated with Selexorb for 5 hours and passed through a plug of neutral alumina before use. Solution NMR was recorded on Bruker 200, 300, 400 and 500 MHz spectrometers at 298K. Electrochemical measurements were conducted inside an Argon filled glovebox using a Metrohm autolab PGSTAT101. An electrolyte solution of 0.1 M $TBAPF_6$ in dry THF was employed with the analyte complex in concentrations of 1 mM.

Synthetic Procedures

1. Synthesis of $[MoO(OC(CF_3)_3)_2py_3]$ **(1)**

$[MoO_2(OC(CF_3)_3)_2(dme)]$ (300 mg, 0.45 mmol, 1 equiv.) was dissolved in 10 mL THF. Subsequently, 1 mL of freshly purified pyridine was added. The mixture was then cooled to -78°C and a solution of 2,3,5,6-tetramethyl-1,4-bis(trimetylsilyl)-1,4-diaza-2,5-cyclohexadiene (141 mg, 0.5 mmol, 1.1 equiv) was added dropwise with vigorous stirring. The reaction mixture was warmed up to room temperature overnight (in the acetone-dry ice bath); it initially turned light purple and then darkened gradually. Subsequently, the volatiles were removed and the residue extracted with toluene. The toluene solution was layered with pentane and cooled to -30°C. The resulting black crystalline solid was then recrystallized again from toluene/pentane to yield **1**. The product could be isolated as a black crystalline solid in 52% yield.

X-Ray quality crystals could be obtained by extracting the initial residue with pentane and cooling to -30°C. NMR: ^1H (C_6D_6, *200 MHz*): 7.11 (b, 6H), 5.81 (m, 3H), 4.93 (m, 6H); ^{19}F (C_6D_6, *188 MHz*): -73.07; ^{13}C (C_6D_6, *50 MHz*): 149.50 (*o*-CH in NC_5H_5; *trans* to NC_5H_5), 148.95 (*o*-CH in NC_5H_5; *trans* to OR_{F9}) 137.85 (*p*-CH in NC_5H_5; *trans* to NC_5H_5), 136.57 (*p*-CH in NC_5H_5; *trans* to OR_{F9}), 128.18 (CF_3, partial overlap with solvent signal), 123.29 ($OC(CF_3)_3$), 122.91(*m*-CH in NC_5H_5; *trans* to NC_5H_5), 122.51 (*m*-CH in NC_5H_5; *trans* to OR_{F9}). EA could not be obtained for this compound due to its slight vacuum sensitivity and high degree of fluorination.

2. Synthesis of $[MoO(OC(CF_3)_3)_2(thf)]_2$ **(3)**

$[MoO_2(OC(CF_3)_3)_2(dme)]$ (300 mg, 0.45 mmol, 1 equiv.) was dissolved in 10 mL THF. Subsequently, 1 mL of styrene was added. The mixture was then cooled to -78°C and a solution of 2,3,5,6-tetramethyl-1,4-bis(trimetylsilyl)-1,4-diaza-2,5- cyclohexadiene (141 mg, 0.5 mmol, 1.1 equiv) was added dropwise with vigorous stirring. The reaction mixture was warmed up to room temperature overnight (in the acetone-dry ice bath). Subsequently, the volatiles were removed and the residue extracted with pentane. The pentane solution was concentrated and cooled to -30°C. The product could be isolated as a dark red crystalline solid in 40% yield.

Catalytic Procedures

Preparation of stock solutions. All olefins were conditioned according to reported purification process.[4] The olefins were used to prepare stock solutions (~1.0 M) in toluene, with decahydronaphtalene (0.1 M) as internal standard. The solutions were stored in a glove-box in flasks with Teflon stopcocks.

Catalytic Tests. All tests were conducted inside a glovebox. 0.003 mmol of **1** was weighed out in a conical base vial equipped with a wingshaped magnetic stirring bar. Subsequently, 1mL of the olefin stock solution was added followed by $B(C_6F_5)_3$ (0.009 mmol for 3 equiv.). At t = 0, the vial was placed in an aluminum heating block kept at the target temperature and the reaction mixture was stirred at 500rpm. 10 μl aliquots of the reaction mixture were drawn, diluted in pure toluene and quenched by addition of wet ethyl acetate after exposure to air. The resulting samples were analyzed by GsC/FID (Agilent Technologies 7890 A) equipped with an HP-5 (Agilent Technologies) column. Conversion was determined from metathesis product formation.

Computational Details

DFT Calculations were performed using the ORCA (v6.1) program package.[5,6] Input structures were generated using XTB optimizations, and stationary points were fully optimized using the B3LYP exchange-correlation functional[7,8] with atom-pairwise D3 dispersion and Becke-Johnson damping,[9,10] in conjunction with the triple-zeta def2-TZVP basis set.[11] The RI approximation was employed for the Coulomb term using the matching def2/J auxiliary basis set throughout.[12] Vibrational frequency analyses verified minima (zero imaginary frequencies), and first-order saddle points (one imaginary frequency along the reaction coordinate) and provided zero-point, thermal and entropic contributions to Gibbs free energies at standard conditions in the gas phase. These were converted to 1 M to reflect standard conditions in solution by subtracting 1.89 kcal/mol accordingly. In steps that involve the addition of another equivalent of propene, the Gibbs free energy is calculated by including only two-thirds of the entropy contribution in that step.

Crystallographic data

The crystal structure of **1** showed significant disorder on both O^tBu_{F9} groups, which were accordingly modelled in two distinct configurations. Additionally, an increased electron density at the central carbon of the O^tBu_{F9} group trans to the oxo required the insertion of a TMS group to stabilize the model. However, this TMS group could not be evidenced spectroscopically and should thus be purely regarded as an aid in modelling the structure.

Identification Code	**1**
CCDC deposition number	2117645
Empirical Formula	$C_{22.69}H_{17.82}F_{15.19}MoN_3O_3Si_{0.32}$
Formula Weight	773.90
Temperature (K)	100
Wavelength (Å)	0.71073
Crystal System	triclinic
Space Group	P-1
Unit Cell Dimensions	a = 9.7717(2) Å α = 89.2540(10)°<br>b = 9.82510(10) Å β = 75.6330(10)°<br>c = 15.0404(2) Å γ = 78.1320(4)°
Volume (Å^3)	1367.84(4)
Z	2
Calculated Density (g/cm^3)	1.879
Absorption Coefficient (mm^{-1})	0.630
F(000)	764.0
Crystal size (mm^3)	0.3 × 0.3 × 0.01
Theta range for data collection (°)	4.24 to 58.054
Index ranges	-13 ≤ h ≤ 13, -13 ≤ k ≤ 13, -20 ≤ l ≤ 20
Reflections collected	52515
Independent reflections	6778
Completeness to theta=50.5°	100%
Absorption Correction	Semi-empirical from equivalents
Max. and min. transmission	0.886 and 0.865
Refinement method	Full-matrix least squares on F^2
Data / restraints / parameters	6778 / 607 / 666
Goodness-of-fit on F^2	1.100
Final R indices [I> 2σ(I)]	R1 = 0.0478, wR2 = 0.1041
R indices (all data)	R1 = 0.0586, wR2 = 0.1085
Largest diff. peak and hole (e•Å^{-3})	0.79 and -0.50

Identification Code	**3**
CCDC deposition number	2482767
Empirical Formula	C22.58H28.78Mo2O8Si1.42
Formula Weight	1101.14
Temperature (K)	100.00
Wavelength (Å)	0.71073
Crystal System	monoclinic
Space Group	P21/c
Unit Cell Dimensions	a= 10.8082(4) Å α = 90°<br>b = 17.7991(5) Å β = 110.743(4)°<br>c = 10.1192(4) Å γ = 90
Volume (Å3)	1820.50(12)
Z	2
Calculated Density (mg/m3)	2.009
Absorption Coefficient (mm-1)	0.898
F(000)	1082
Crystal size (mm3)	0.2x0.2x0.1
Theta range for data collection (°)	4.634 to 59.998
Index ranges	-15≤h≤15, -24≤k≤25, -14≤l≤14
Reflections collected	67950
Independent reflections	5288
Completeness to theta=50.5°	99.4
Absorption Correction	Semi empirical from equivalent
Max. and min. transmission	0.7482 and 0.6038
Refinement method	Full matrix least squares of F2
Data / restraints / parameters	5288/484/368
Goodness-of-fit on F2	1.065
Final R indices [I> 2σ(I)]	R1 = 0.0703, wR2 = 0.1867
R indices (all data)	R1 0.0744, wR2 0.1945
Largest diff. peak and hole (e•Å–3)	3.21/-1.18

Catalytic Details

Conversion over time obtained under different conditions explored (see Table S1) are shown in Figures S8-11.

Table S1. Catalytic activity of complex **2** with 300 equivalents of substrate in toluene

Substrate	equivalents $B(C_6F_5)_3$	Conversion after 24 h (30°C)	Conversion after 24 h (70°C)
1-nonene	0	0.0%	0.0%
1-nonene	2	-	0.7%
1-nonene	3	20.9%	34.0%
1-nonene	4	--	58.2%
4-nonene	3	0.3%	18.5%
styrene	3	--	1.4%
allylbenzene	3	--	37.0%
□-methylstyrene	3	--	8.4%

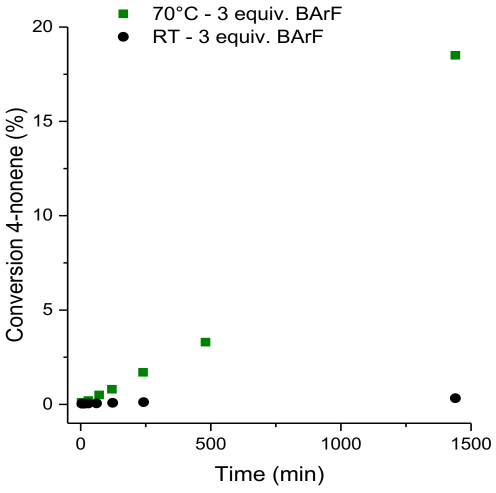

Figure S1. Self-metathesis of *cis*-4-nonene (300 equiv) catalysed by **1** in the presence of $B(C_6F_5)_3$.

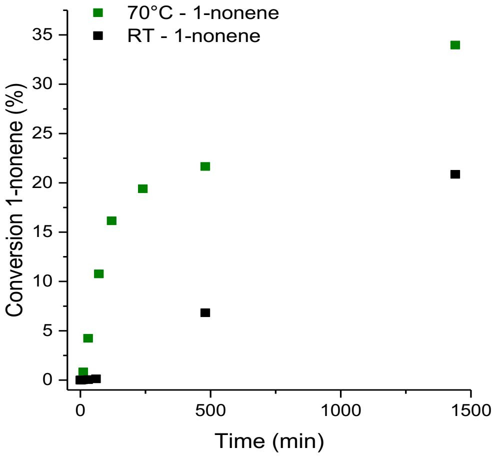

Figure S2. Self-metathesis of 1-nonene (300 equiv) catalysed by **1** in the presence of $B(C_6F_5)_3$.

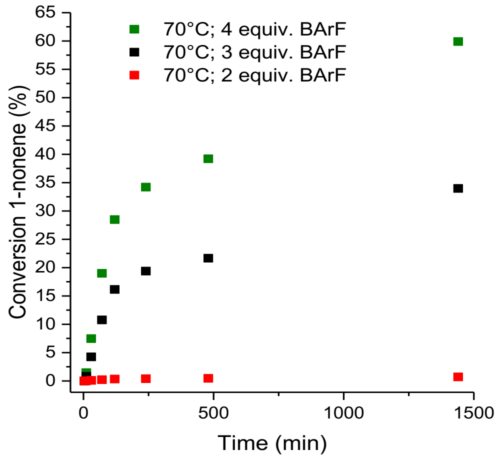

Figure S3. Self-metathesis of 1-nonene (300 equiv) catalysed by **1** in the presence of different amounts of $B(C_6F_5)_3$.

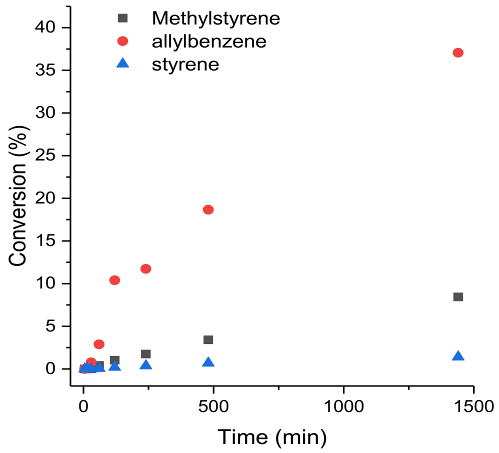

Figure S4. Self-metathesis of styrene, methylstyrene and allylbenzene (300 equiv) catalysed by **1** in the presence of $B(C_6F_5)_3$.

NMR Spectra of compounds

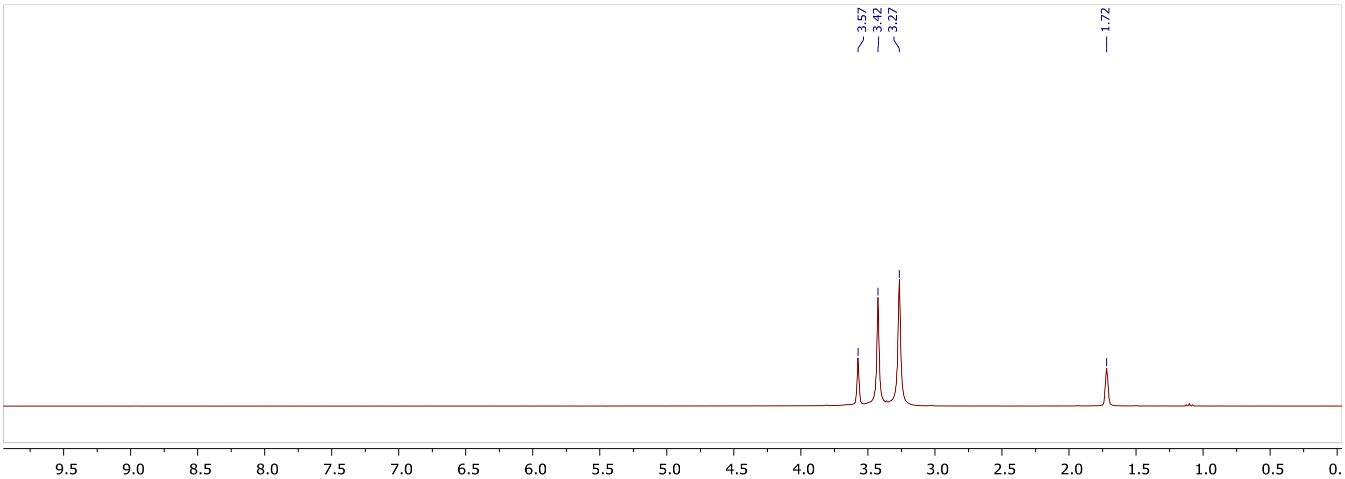

Figure S5. ^{1}H NMR (THF-d8, 200 MHz) spectrum of $MoO_2(O^tBu_{F9})_2(dme)$ **2**

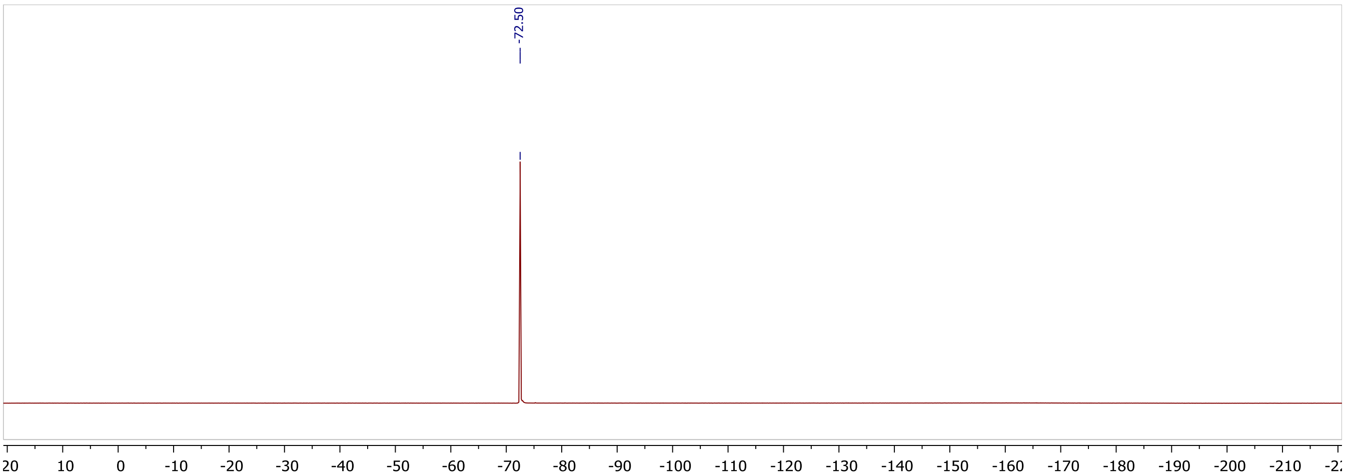

Figure S6. ^{19}F NMR (THF-d8, 188 MHz) spectrum of $MoO_2(O^tBu_{F9})_2(dme)$ **2**

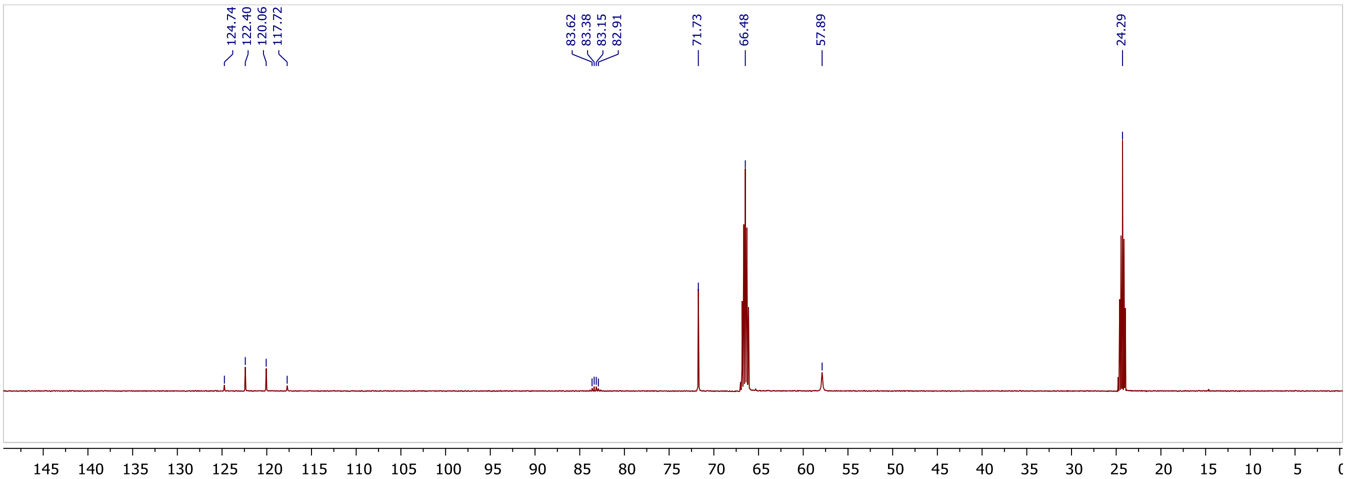

Figure S7. $^{13}C\{^{1}H\}$ NMR (THF-d8, 50 MHz) spectrum of $MoO_2(O^tBu_{F9})_2(dme)$ **2**

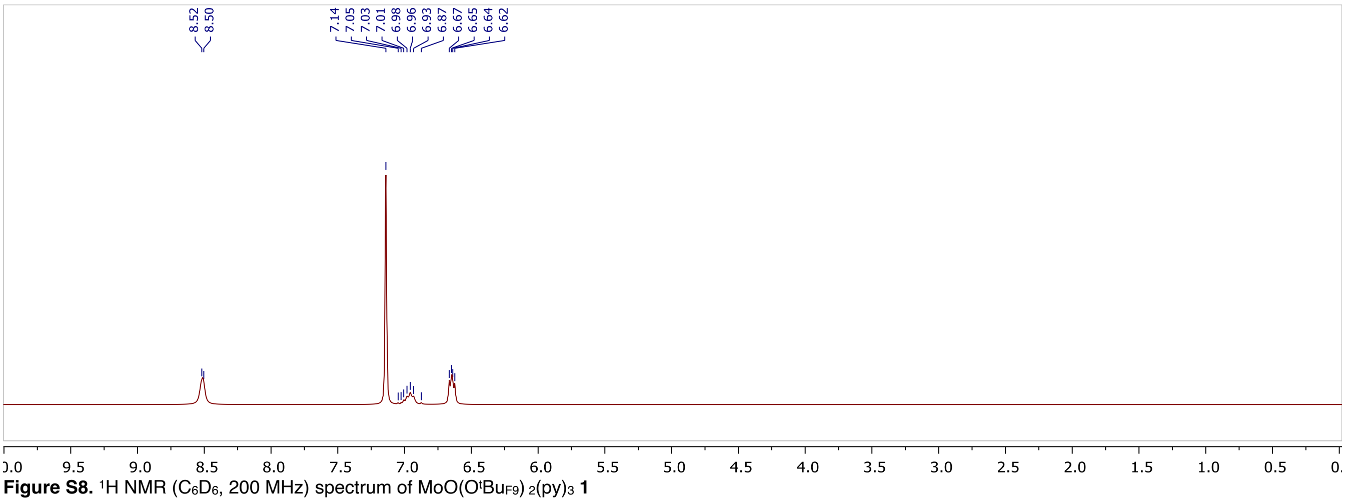

Figure S8. ^{1}H NMR (C_6D_6, 200 MHz) spectrum of $MoO(O^tBu_{F9})_2(py)_3$ **1**

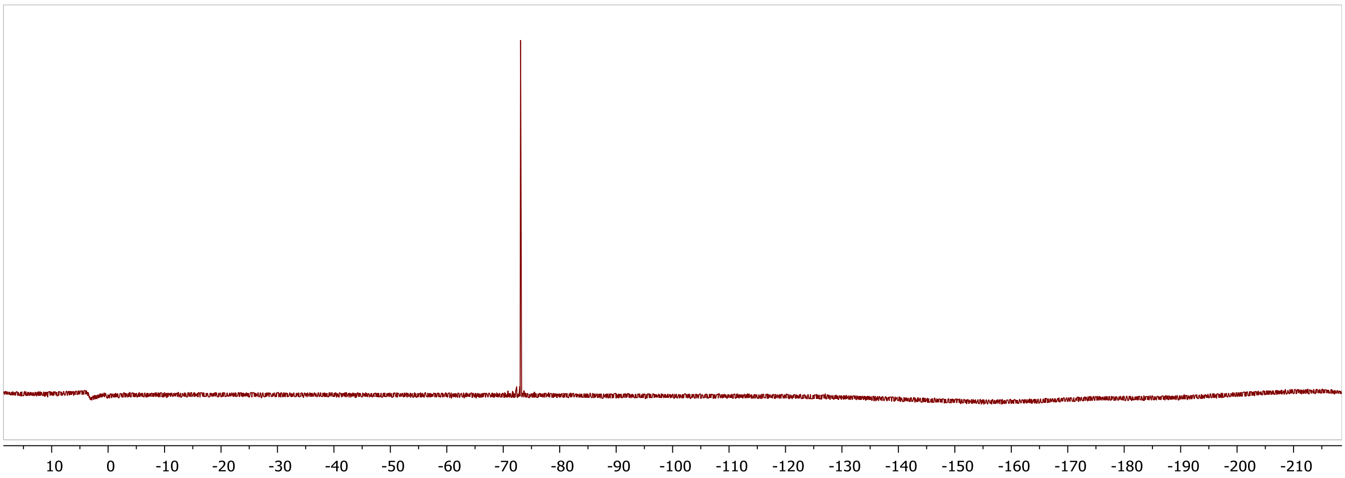

Figure S9. ^{19}F NMR (C_6D_6, 188 MHz) spectrum of $MoO(O^tBu_{F9})_2(py)_3$ **1**

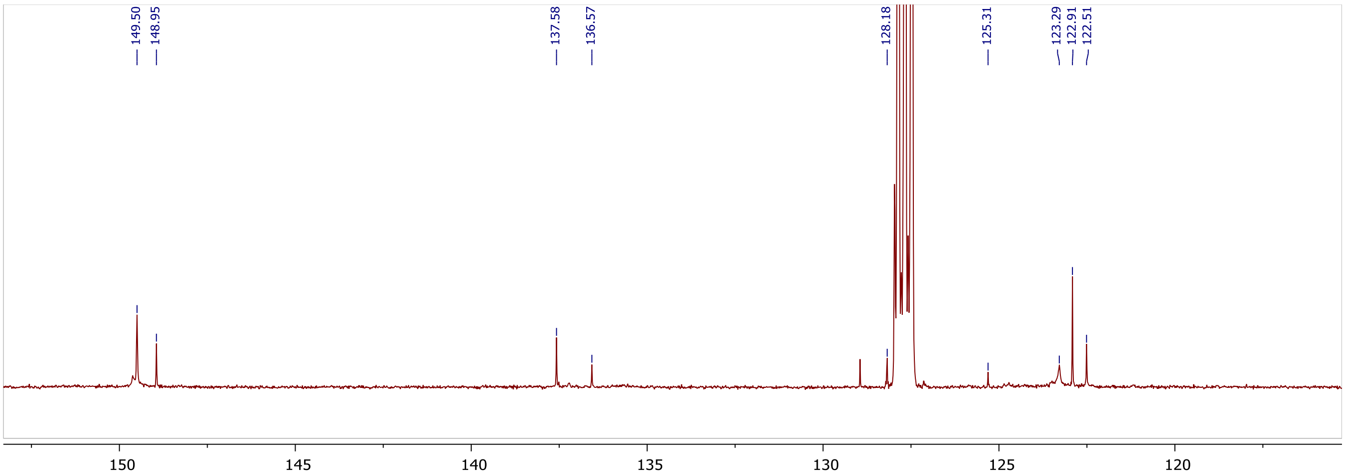

Figure S10. $^{13}C\{^{1}H\}$ NMR (C_6D_6, 50 MHz) spectrum of $MoO(O^tBu_{F9})_2(py)_3$ **1**

Presence of py·$B(C_6F_5)_3$ Adduct

The interaction of complex **1** with $B(C_6F_5)_3$ was investigated to evaluate the formation of a pyridine–borane adduct. To this end, two complementary experiments were conducted. In the first, a catalytic reaction mixture was concentrated under reduced pressure, and the residue was analyzed by ^{19}F NMR spectroscopy (Figure S8). The resulting spectrum exhibited distinct signals attributable to the pyridine- $B(C_6F_5)_3$ adduct at -131, -155 and -163 ppm, thereby confirming its formation under catalytic conditions. In a separate experiment, a stoichiometric 3:1 mixture of $B(C_6F_5)_3$ and complex **1** in C_6D_6 was analyzed by both ^1H and ^{19}F NMR spectroscopy (Figure S9 and S10). The spectra again revealed resonances consistent with the formation of the pyridine-borane adduct. These findings provide spectroscopic evidence for the interaction between the Lewis acidic borane and the pyridyl moiety in complex 1.

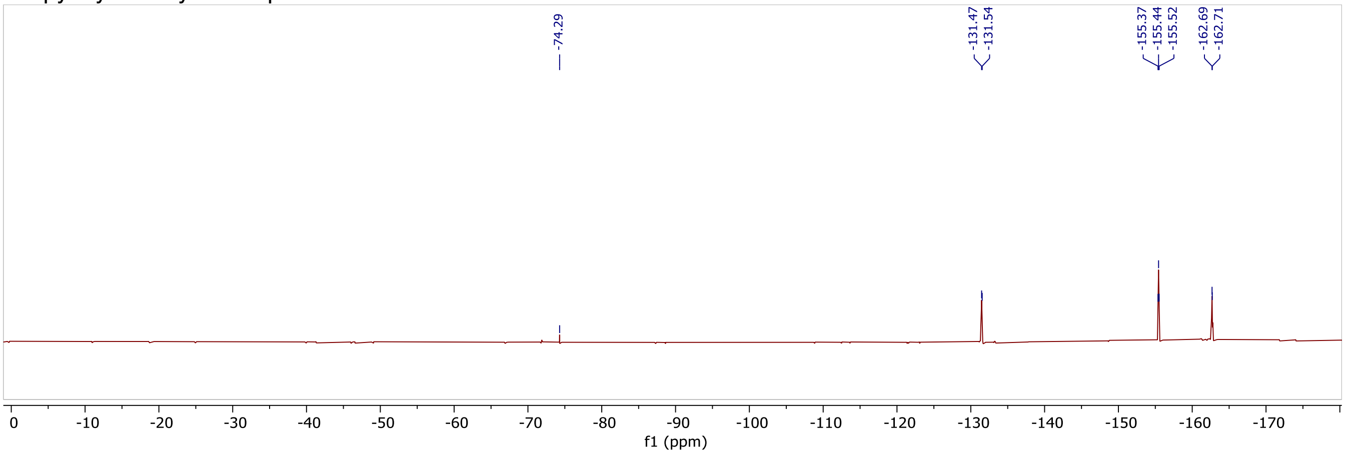

Figure S11. ^{19}F NMR (C_6D_6, 282 MHz) spectrum of catalytic mixture after 24 hours.

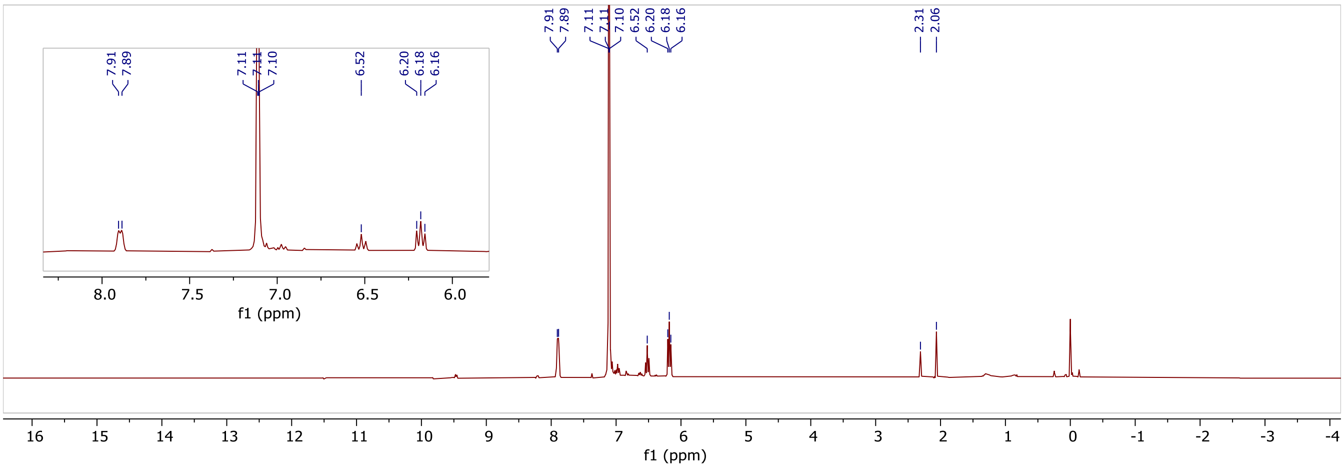

Figure S12. ^1H NMR (C_6D_6, 300 MHz) spectrum of 3:1 mixture of $B(C_6F_5)_3$ and complex **1**.

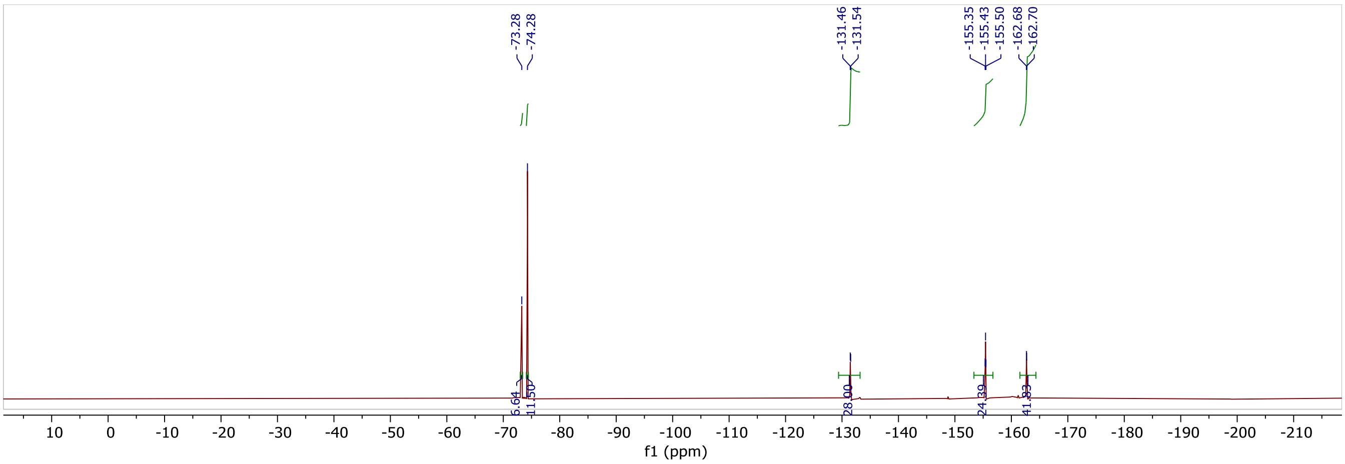

Figure S13. ^{19}F NMR (C_6D_6, 282 MHz) spectrum of 3:1 mixture of $B(C_6F_5)_3$ and complex **1**.

Computational Results

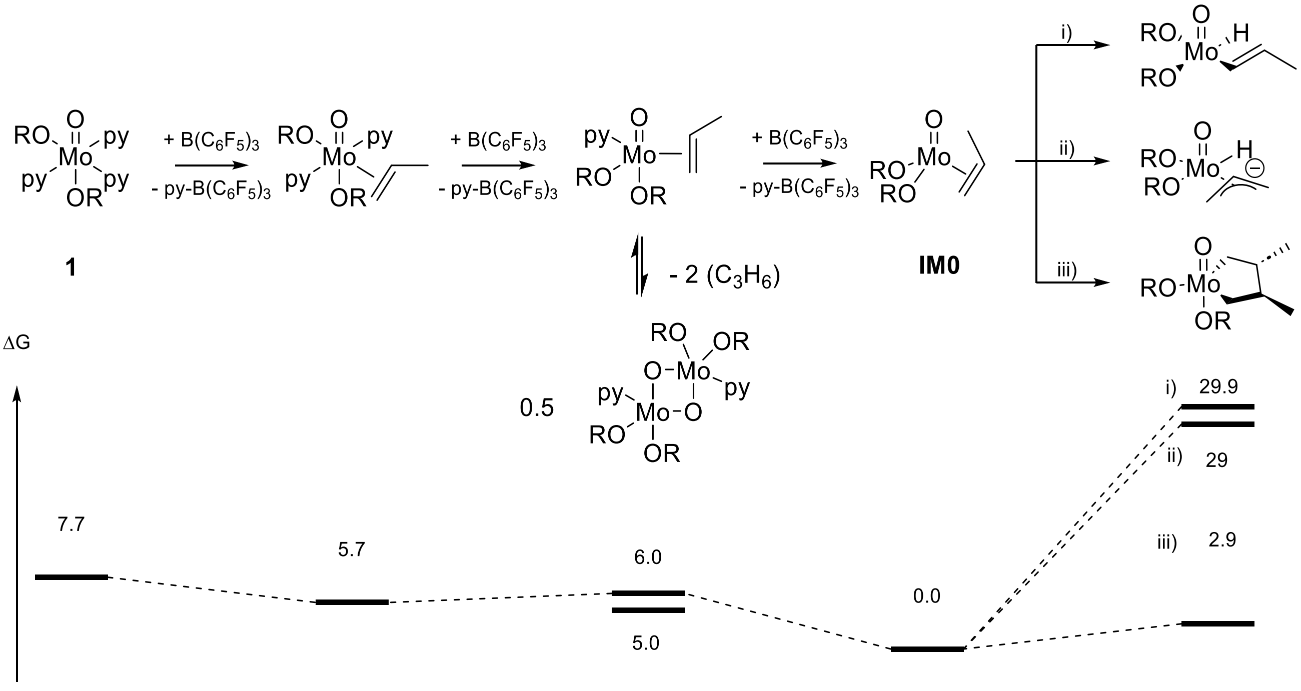

Scheme S1: Gibbs free energies of the of the removal of pyridine by $B(C_6F_5)_3$ from **1**; forming olefin complex **IM0**. All values given in kcal/mol. Energies relative to IM0 as 0.

Influence of substitution pattern on the energies of metallacyclopentane species

Scheme S2: Intermediates and Transition states investigated with regards to the influence of the substitution pattern of the metallacyclopentane. Energies relative to IM1 as 0.

	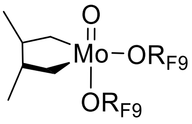 1	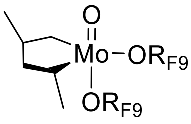 2	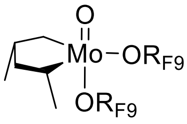 3	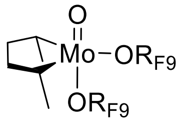 4	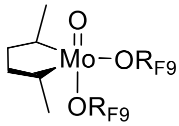 5
**TS1C**	[a]	ΔG‡ = +17.1 kcal/mol	ΔG‡ = +18.4 kcal/mol	[a]	ΔG‡ = +22.8 kcal/mol
**IM2C**	ΔG = -0.9 kcal/mol	ΔG = +0.4 kcal/mol	ΔG = +1.0 kcal/mol	ΔG = +5.8 kcal/mol	ΔG = +3.8 kcal/mol
**TS2C**	ΔG‡ = +44.3 kcal/mol	ΔG‡ = +38.8 kcal/mol	ΔG‡ = +41.0 kcal/mol	ΔG‡ = +39.2 kcal/mol	ΔG‡ = +41.4 kcal/mol
**TS4C**	[b]	ΔG‡ = +21.4 kcal/mol	ΔG‡ = +20.7 kcal/mol		[a]

	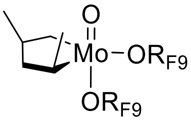 6	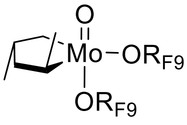 7	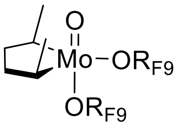 8	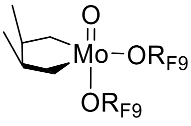 9	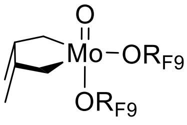 10
**TS1C**	[a]	[a]	[a]	[a]	[a]
**IM2C**	ΔG = +2.4 kcal/mol	ΔG = +1.6 kcal/mol	ΔG = +6.0 kcal/mol	ΔG = +0.1 kcal/mol	ΔG = -0.2 kcal/mol
**TS2C**	ΔG‡ = +46.6 kcal/mol	ΔG‡ = +46.3 kcal/mol			

[a] Structure not obtained; [b] Transition state not applicable

Potential energy surfaces of Pathway C

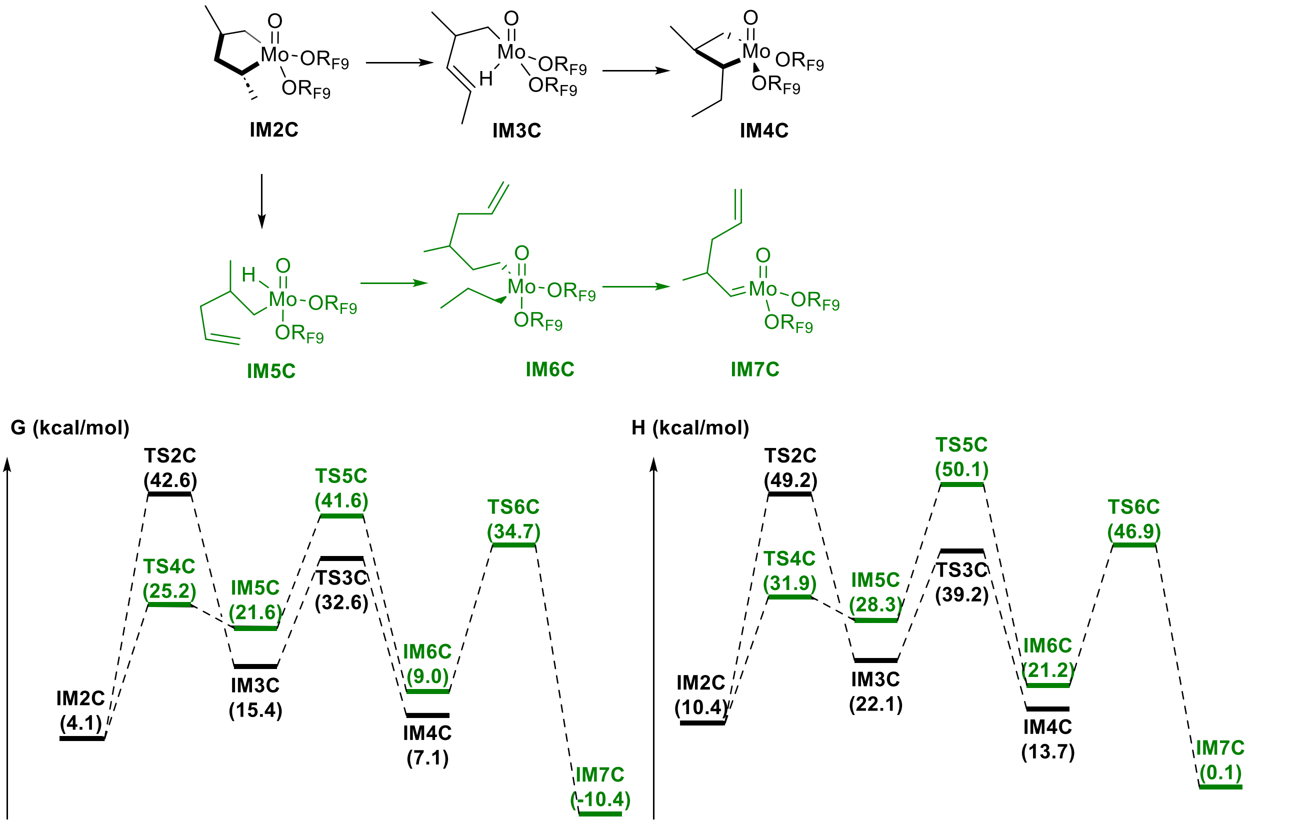

Scheme S3: Minimum-energy reaction pathway for the catalyst activation through molybdenum hydride intermediates from metallacyclopentene intermediate IM2C, calculated at the B3LYP-D3(BJ)/def2-TZVP level of theory. Gibbs free energies, and enthalpies in kcal/mol relative to intermediate IM0.

Pathway D, unassisted

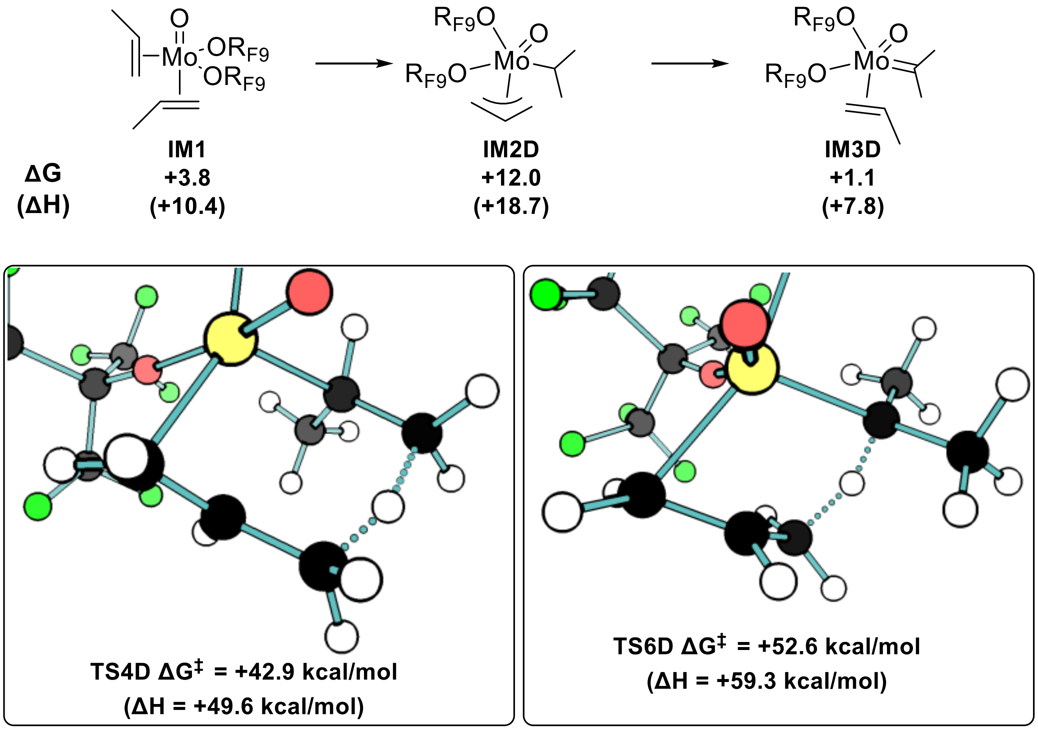

Scheme S4: Minimum-energy reaction pathway for the catalyst activation through un catalyzed proton transfer, calculated at the B3LYP-D3(BJ)/def2-TZVP level of theory. Gibbs free energies, and enthalpies in kcal/mol relative to intermediate IM0. Enthalpies in brackets relative to IM0.

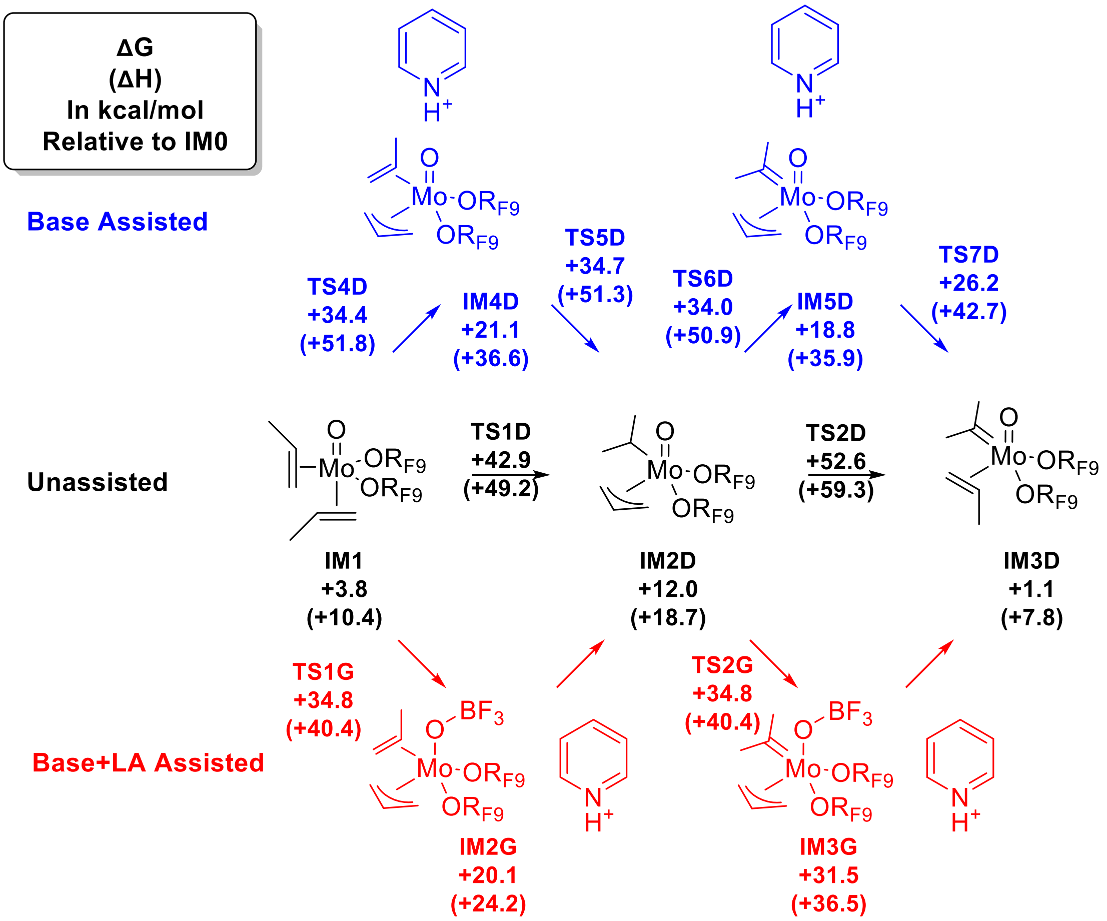

Scheme S5: Minimum-energy reaction pathway for the catalyst activation through pyridine catalyzed proton transfer, calculated at the B3LYP-D3(BJ)/def2-TZVP level of theory. Gibbs free energies, and enthalpies in kcal/mol relative to intermediate IM0. Enthalpies in brackets relative to IM0.

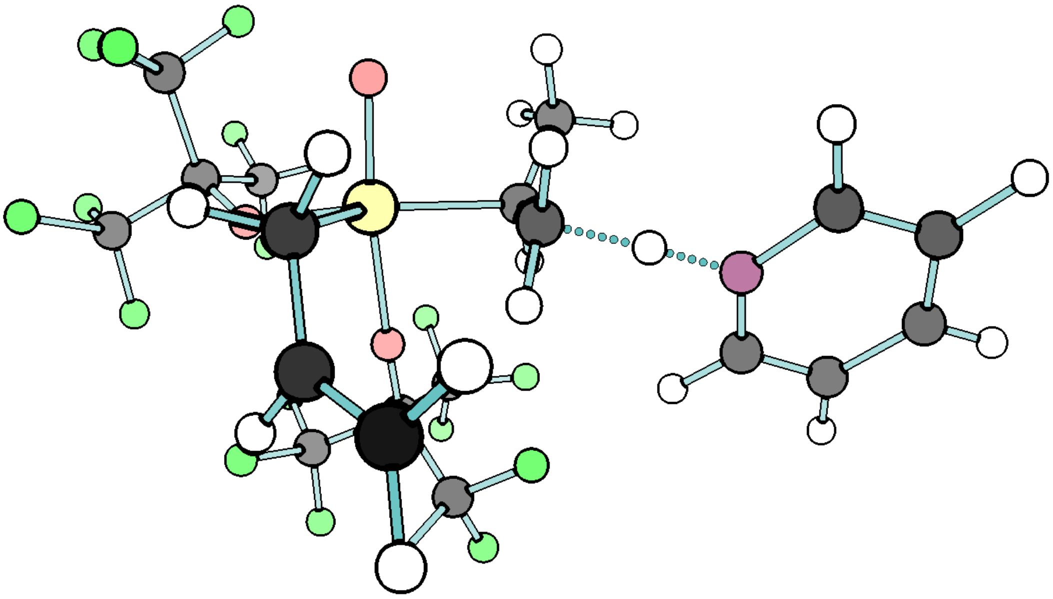

Scheme S6: Structure of transition state TS5D, with dashed bonds corresponding to bonds involved in the imaginary frequency of the saddlepoint.

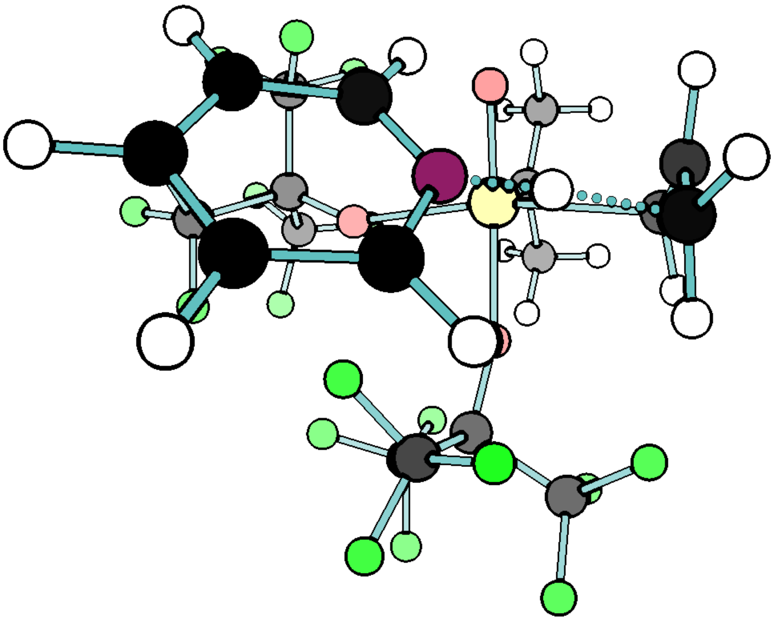

Scheme S7: Structure of transition state TS7D, with dashed bonds corresponding to bonds involved in the imaginary frequency of the saddlepoint.

Coordinates of calculated structures

Propane

C	0.98137794592804	1.24367806253417	-1.72650358934784
H	1.86294782540139	0.61628800606127	-1.88230261298555
H	0.09980385732952	0.61629418762391	-1.88230675182717
C	0.98137646626977	1.85058190126225	-0.32542810701984
H	1.85608796035814	2.49786647815573	-0.20904004499544
H	0.10666403733026	2.49786480175909	-0.20903927169332
H	0.98138190441708	2.01585791785096	-2.49859477730653
C	0.98137874167394	0.79398590205249	0.77688068019722
H	0.09981483142093	0.15155400509998	0.70479663407405
H	1.86294324140563	0.15155532404221	0.70479539767205
H	0.98137865846529	1.24933140155795	1.76937506323238

Propene

C	2.53111971694576	-2.25081839152242	-3.10800744575008
H	2.73706569345164	-3.21187425532257	-2.64288958500535
C	2.36371421459028	-1.18761894694375	-2.33174539463687
H	2.15640325865935	-0.20821232307946	-2.74954767480320
H	2.42732579188113	-1.25550133686134	-1.25311126113531
C	2.46059661484750	-2.24699278929760	-4.60242334035289
H	3.40215227303722	-2.59201313415681	-5.04022056407672
H	1.68152267997197	-2.92692474768432	-4.96003330133092
H	2.24846583961512	-1.24928560113172	-4.98986772890867

Pyridine

C	-1.45631734365717	0.71198374974056	-0.69006818616758
C	-1.45631725567296	1.90547487624731	0.01923439035545
C	-1.45631691966043	1.85146564696556	1.40866566918998
N	-1.45631701940115	0.71198373691602	2.10098880621301
C	-1.45631703403729	-0.42749810435610	1.40866555971685
C	-1.45631620252770	-0.48150748941351	0.01923429049509
H	-1.45631728212525	0.71198352924120	-1.77281635782379
H	-1.45631677108695	2.86081560592122	-0.48904679165358
H	-1.45631504182196	2.76629238364574	1.99239102642690
H	-1.45631779264982	-1.34232483115354	1.99239115244655
H	-1.45631466335930	-1.43684808475445	-0.48904709219890

Mo(py)(propene)(O)(OR)2

Mo	-0.08938323354273	1.07526151065791	-1.00845121966165
F	0.96979396996134	-2.69942921638001	-1.37404714969554
F	3.06729106546880	-3.09555560427360	-1.00778812901801
F	2.47451474707915	-1.48629191858852	-2.33463400723873
F	-1.24850459902200	-2.55125003197770	0.52258150377113
F	-3.03183930925614	-2.40922857521150	1.74081950346430
F	-1.31643579404688	-1.16448837813374	2.18272508931103
F	-3.56352905626697	-2.39013698504017	-1.01020944105198
F	-1.86734527572512	-1.36757557573624	-1.90058745619843
F	-3.79743478065675	-0.40197430799574	-1.82742278978450
F	-4.10050621205195	1.26470175183678	0.26004096086715
F	-3.56381786336114	0.41627849725718	2.16997937384874
F	-4.90462557281850	-0.65872709605648	0.84313662003321
F	3.23121054426400	0.75535931806536	-1.02727548210407
F	4.50090504915779	-0.83346319657256	-0.30424364890960
F	3.52255581791097	0.50425771755000	1.09487745032852
F	3.41215429099197	-2.33986982177149	1.65022604635882
F	1.36805955232307	-2.97523956700273	1.33727966277056
F	1.77546982804135	-1.13013293769160	2.38439335341742
O	-1.60651695359631	0.37318110304510	0.06261819662428
O	0.93359256010138	-0.43014060764143	-0.00310060917598
O	0.78393580988940	1.02133809614592	-2.42316716227732
N	1.01291282364932	2.32627911769080	0.31471960436062
C	-1.07715157590090	3.11226765178770	-1.42173827819951
C	-1.75451675517033	2.13915995110491	-2.15396934408155
C	2.09802579484356	-1.10507587105069	0.00895065628404
C	2.15965557634593	-2.12270770189811	-1.19462415491099
C	2.17196202171093	-1.90769516868959	1.36582084990945
C	3.36487053201283	-0.16854781913064	-0.06441498669789
C	-2.56344250287843	-0.57038863257568	0.23486863943973
C	-2.03019947123021	-1.70376269359085	1.19051618993329
C	-3.81457500027167	0.11478832850404	0.88787893323132
C	-2.96530145993258	-1.20562119670316	-1.14531636220661
C	0.86877911052829	2.07901616114242	1.62949599500886
C	1.56449580795078	2.80052742469589	2.58218637891532
C	2.45573786824145	3.78020789938398	2.17217098911275
C	1.87287127039203	3.27637181518701	-0.08936761036112
C	2.61474056408946	4.01318465918229	0.81125290850714
C	-1.67784073983087	3.77665763622941	-0.20823217146516
H	0.19502647610139	1.27894073564953	1.89983162790916
H	1.41523839060272	2.57280741190528	3.62789527656628
H	3.30656804396254	4.75754416437282	0.44342747690074
H	3.02312268073240	4.34786734070594	2.89749428335326
H	1.96231610048554	3.42145744539648	-1.15536369181705
H	-0.31680954159994	3.69453493641438	-1.93421135875864
H	-2.72375654239676	1.79889905704579	-1.82198159647147
H	-1.52865158554436	1.98578843369224	-3.20049572595210
H	-0.91622180453353	4.15838302422375	0.47290224135554
H	-2.32464721313319	3.09464721128746	0.34215898871321
H	-2.28717345407113	4.62941450355184	-0.52204642425799

Mo(py)2(propene)(O)(OR)2

Mo	0.03412578930441	0.10261416176392	1.47868668981390
F	-1.41286819695532	-0.94297834710680	-2.78368823054982
F	-1.85598559522941	-2.11852530917708	-1.02894215776925
F	-3.46878617741019	-1.41017662921752	-2.28520673539303
F	-1.25314780003652	1.76224320945619	-2.40373612919474
F	-3.31103805582810	1.28731524787842	-2.86997483486914
F	-2.84050474918282	2.56520735814986	-1.18153500612640
F	4.84094309461307	-0.61268193230726	-1.09015893031151
F	4.03396682884047	1.11709277027672	-0.06316058969743
F	3.97438206195612	-0.82507960692106	0.87850692280990
F	2.90403057868061	-2.78918780155741	-0.63991893261901
F	1.12127816030575	-2.28173443130226	-1.74904189681217
F	3.06654416681850	-2.00165003879174	-2.65231082509550
F	1.82409805023552	1.77419231873409	-1.59336779307723
F	3.25085143672408	0.73721340114905	-2.84305024105921
F	1.16395627430760	0.15945714043781	-2.86114470597347
F	-3.54565966993052	1.07595888094632	0.99416009065035
F	-4.74848322213041	0.45163828038343	-0.69145596603944
F	-3.78125673980125	-1.02496816769452	0.56089176630988
O	-1.08506003333686	0.45221949018189	2.67671155906174
O	-1.21960973755229	0.38733941202672	-0.15931537586211
O	1.48786715209303	-0.31999275958695	0.07077770676220
N	-0.60144654168003	-2.04198021253633	1.61125001975482
N	0.28951942663083	2.32322648544552	1.15398731189169
C	1.41948114180698	1.51714420700134	4.05728086684450
C	0.12658665008670	-3.04601148021133	1.10420652120794
C	-0.29084430466157	-4.36354184488689	1.14339010469911
C	-1.50375056787004	-4.66840638965156	1.74380298666609
C	-1.76671491523987	-2.33842001205027	2.20689466112639
C	-2.24668773807718	-3.63299099591998	2.29161974040357
C	-0.84552043451183	4.41806629745022	0.98855538909136
C	-0.83775131143600	3.04995896778140	1.19570556155499
C	0.35024746005065	5.06722423939656	0.71935291159323
C	1.51554984243289	4.31552809016995	0.67545588569730
C	1.44325355716497	2.95275416242701	0.90177808815612
C	2.37638066612560	-1.89978492400794	-1.51128491305297
C	3.84638083187580	-0.19912367889541	-0.29863397496768
C	2.15837498846913	0.57312662887371	-2.07538515062625
C	2.41113120746952	-0.44901740098396	-0.89823183850281
C	-2.43817662058910	1.47378190122421	-1.86716551972150
C	-2.27183678200750	-1.07922117790789	-1.76775279370629
C	-3.62686863257376	0.18719024196183	-0.00676691781481
C	-2.32266127531141	0.23250352979829	-0.89459589415378
C	1.46182463887513	-0.79504017926340	2.99519850009054
C	1.75948537701537	0.56267693013581	2.94481918674399
H	2.25011079272726	1.56266310324074	4.76916850199080
H	1.24623345407537	2.53094966982044	3.69452187672984
H	0.52789139943871	1.19359383730497	4.59346197978567
H	0.89021955795305	-1.19866996217704	3.82141855676296
H	2.07576758273069	-1.50504067926340	2.45912999307413
H	2.59941052342900	0.85338887111338	2.32572804472026
H	1.06742369546837	-2.77518477176050	0.66229195036108
H	0.33489854342235	-5.12960802325815	0.70749853624267
H	-1.85948275258077	-5.68943002483700	1.78781047328164
H	-2.31401041498205	-1.50559078529169	2.61912452887436
H	-3.19542135640515	-3.81399949976531	2.77708214083317
H	-1.74677443569023	2.50943257226433	1.39928900145483
H	0.37378095842770	6.13527355978314	0.54605734821196
H	-1.78326430823046	4.95390498142228	1.03022293126201
H	2.47442887548574	4.76859234800566	0.46625209537927
H	2.32898760419964	2.34283472784738	0.87215492310123

[Mo(O)(py)(OR)2]2

Mo	-0.17399192441239	0.32855419939260	2.05901024864763
F	-1.48365689463979	-3.92302042025112	2.83880692489175
F	-2.09944017292488	-2.25060348387449	4.05541094626089
F	-3.59142718722777	-3.54205379642185	3.15835996984936
F	-1.08990193919270	-3.36602542508430	0.21549455572836
F	-3.16178236704075	-3.95449214439107	0.43146011008577
F	-2.64114741789979	-2.16610058166698	-0.68424870043264
F	4.70125995787025	-0.81852703590081	2.38941981942825
F	3.74004000220484	0.81013355887789	1.33537142888219
F	3.14408687896832	0.34094814543183	3.35861806191789
F	1.01087287470796	-1.35766832114868	3.57898653282113
F	1.24932802970652	-3.13067813856909	2.35829917266354
F	2.86661625686558	-2.48236512180789	3.63364643327159

F	3.72423047555897	-1.33063180520042	-0.35558929465255
F	3.79665573498431	-3.03021022734655	0.99129806195163
F	2.01801872228011	-2.64315188735034	-0.18410212952233
F	-3.57561890922531	-0.10816845657528	0.88362240170919
F	-4.76874410854414	-1.88580903088172	1.21220594426583
F	-4.04504713606828	-0.74235571724771	2.89832696380786
O	-1.31082070799219	-1.22948532138108	1.70964173273190
O	1.40759481167463	-0.41328185694389	1.09465695428214
O	0.42425335997495	1.70497321730683	2.92241133243752
C	1.91056410199341	-2.07096901564246	2.80753820729054
C	3.53330111335923	-0.19097736092062	2.18706581692077
C	3.00441622760546	-2.06188382587371	0.50261412569341
C	2.41797986373658	-1.15581094401340	1.63974110669059
C	-2.32973391814467	-2.90841172744329	0.39419860611824
C	-2.40507509605877	-2.96055286947190	2.95651667369559
C	-3.73032185970883	-1.17370748955481	1.67540305249092
C	-2.40527134675579	-2.02741936494627	1.69519075006836
Mo	-1.35226214604641	2.51090916168495	4.05851854826395
F	-5.17825757985059	4.15300832583102	6.59546398024678
F	-3.47103362626829	5.45885467477441	6.38747292622832
F	-5.28290054311952	5.86955084217361	5.27214161487560
F	-5.25521355113197	2.03228622232134	4.88095692975911
F	-6.24236092936976	3.67773048020529	3.87799840698702
F	-4.72199328829279	2.52374499957783	2.84511071756924
F	2.01180582308247	6.33805143638915	2.79843674985284
F	0.51049330494777	4.99834290159819	1.99088524383060
F	-0.09102960377161	6.71366713302176	3.15356165168565
F	-0.40319030596654	6.27506875666326	5.80246247578626
F	1.15843654390462	5.09812837173303	6.71513659293013
F	1.66612681772261	6.84387055186902	5.52642630911244
F	2.50662098220436	3.56644880323142	3.15189327939393
F	3.25185418857726	4.76524516176574	4.78797324634995
F	2.08095550697310	2.98870178626144	5.19320729029914
F	-2.58293247486894	4.21778757067775	2.58302861959666
F	-4.43911893490221	5.34293074079568	2.59166191276962
F	-2.78671866003561	5.97876857304052	3.82722372311230
O	-2.91319496862408	3.24156636329466	5.06322015459084
O	-0.21195203750979	4.06930265879299	4.40172387420784
O	-1.97241080802473	1.15324297720681	3.18185188767970
C	0.83001835694050	5.80471309233501	5.61670351179961
C	0.83621095989394	5.75462386135297	3.05197958325940
C	2.21071922432942	4.03142507075008	4.36681328381314
C	0.87731072149359	4.87420156942874	4.34940388777722
C	-5.07111222041675	3.04310923883130	4.03540000134855
C	-4.48089717446833	4.89116470319167	5.72468919357300
C	-3.46036873869273	4.92371192777294	3.38667313537942
C	-3.93481346316550	3.99620886578843	4.55896075374511
C	-1.47032705282823	2.56589692893513	-2.06904663330021
C	-1.29660052669974	3.31055204302504	-0.90883721708548
C	-0.92287218329590	2.67235529764659	0.25550861594396
N	-0.70858264171417	1.34232048458019	0.30746031150774
C	-0.83952336194902	0.62111099741161	-0.82671908703104
C	-1.23382462240290	1.19906350997966	-2.01762283581257
H	-1.77241770032713	3.04141117502873	-2.99221931456840
H	-1.45050161975807	4.38006929141050	-0.89375471731553
H	-0.76719567540449	3.21923199338508	1.16981716667949
H	-0.61780764013369	-0.43206391200778	-0.75514330516278
H	-1.33907445589931	0.57416478569929	-2.89312276751974
C	-0.04538923404499	0.13763258031923	8.10734728938218
C	-0.13251228020482	1.52259540878162	8.06772095266903
C	-0.52323376593022	2.14773650371596	6.90019284457388
N	-0.80027179825918	1.45243911797413	5.77582973375606
C	-0.73382042253167	0.10560135349245	5.81766108944548
C	-0.36386938388392	-0.57582937569981	6.95881158222077
H	0.25240906907433	-0.37350594169780	9.01276139026273
H	0.08945671288587	2.12767237906243	8.93524788371985
H	-0.62165773773803	3.21987676575240	6.83973575723119
H	-1.00674310107605	-0.41512019965212	4.91533834647145
H	-0.33003608507686	-1.65582144060355	6.93505544211209

Mo(O)(py)3(OR)2

Mo	-0.18085972639736	0.71773371341391	0.86579177520072
F	-2.17201211422504	0.18895058815018	-3.34761935038211
F	-3.79840416993410	-1.95198486294655	-0.26638782117398
F	0.03974098683936	-2.74639290310347	-1.54725320409098
F	-2.82670146125782	-3.44566334767136	-1.49945776372468
F	4.85025900528325	0.72425406830425	1.03985494447711
F	-3.65667515077898	-1.34470605261381	-3.00947960671340
F	4.42377242264540	0.74964274381202	-1.62259760706636
F	-1.10595969741997	-2.49893941950254	-3.37263276438383
F	-3.56163108548686	0.38303768672805	-1.70697302590321
F	2.35097118205333	1.33072904762130	-1.81619566611661
F	4.73659050893251	-1.67978883605965	-0.30169364194316
F	0.19149879446752	-0.91173524570756	-2.68342743090010
F	-1.93508970302662	-2.83094902636190	0.36852020771544
F	3.53793914421215	-1.92478333375266	1.48837941420338
F	3.06881651818499	0.59269106521921	2.25274273463076
F	2.75256413500986	-2.53637656592474	-0.42413328684380
F	2.96299743220365	-0.66612955468663	-2.36153713521911
F	3.20264617356690	2.09205162906193	0.69903044034156
O	0.44027497958197	1.67890717404081	2.09385017375650
O	1.51870965364877	-0.29227232671457	0.20879671694714
O	-1.29355100884223	-0.36671913225394	-0.52972985296079
N	-2.16998889218255	1.52339516676427	1.47260417800908
N	-0.32239657550509	-0.99004855242596	2.27774826819036
N	0.00768350070075	2.41935462615439	-0.51660277777544
C	-0.65175639783282	-1.86211604390110	-2.27947101493798
C	-2.82186249985407	-0.49993937859510	-2.39029362871234
C	-2.59881616721247	-2.39445085560593	-0.70495802386330
C	3.48814092713170	-1.59731704399710	0.18390620768660
C	3.51007939208798	0.81821673028767	1.01434519445675
C	3.15876954266838	0.31806078802491	-1.47312886575404
C	0.23692001184171	4.62059540529674	-2.22212300732439
C	-3.44588558206260	3.33106422708818	2.39505510845894
C	-4.62462306433687	2.60937178476103	2.25125121310826
C	0.24340056355862	-3.22550416691713	2.89189074634934
C	-0.31554587159863	-3.04251851622942	4.14703019159759
C	-4.54553907087378	1.32996393560739	1.72036701136271
C	0.22625910862168	-2.17838941416542	1.98553206561327
C	2.83759473819490	-0.17269461450767	-0.00798470801608
C	0.67994797252849	4.68554823568623	-0.90773634385183
C	-0.87755649718010	-1.80798729989758	4.45233254582592
C	-0.31580324206169	3.42753980794578	-2.66500679572052
C	-2.25045927703412	2.76215172621160	1.99812409667525
C	0.55235668082231	3.57735755332940	-0.09199026805884
C	-3.31138088641612	0.82416280848991	1.35183602189127
C	-0.86390837136709	-0.81260998642384	3.49403418532853
C	-0.40948297746718	2.35869267194225	-1.79343815549706
C	-1.80811707148939	-1.23020313017441	-1.42095454823273
H	0.32858565693722	5.47211440989633	-2.88322422578153
H	-3.44446595608236	4.33054277416990	2.80809963987335
H	-5.57521654069606	3.03157469242219	2.54916683260726
H	0.69920778750854	-4.16359768925690	2.60785031316598
H	-0.31198650839132	-3.84224521577503	4.87630320775959
H	-5.42583172017609	0.71546397110546	1.59157926734085
H	0.66324714894346	-2.26710944893209	1.00464758746800
H	1.13284129599777	5.58314359442072	-0.50946724548377
H	-1.32019736073607	-1.61346962578700	5.41940065081040
H	-0.66865374658838	3.30934244698527	-3.68012466942162
H	-1.31575575202497	3.29020905051292	2.10723145900273
H	0.90686211269135	3.58303725542169	0.92755599043670
H	-3.21696966940506	-0.16610200539588	0.94614097508828
H	-1.28102787924647	0.16439393726555	3.69058567243376
H	-0.80869568167421	1.41534827914548	-2.11415260195982

B(C_6F_5)$_3$

F	0.39529271948349	-0.92182017532822	5.12106489522273
F	0.66550873700174	1.54523860870756	4.01971341144331
F	-2.91115400980535	-2.00449599927724	1.99968366346586
F	-1.40198636874155	-2.68464898094956	4.10297580441043
F	-0.84749750841034	2.25978316447752	1.93034478021483
F	-3.88747277739466	2.09349271643445	2.88282322647838
F	-4.90044549910245	4.56417423442038	2.71148227437023
F	-4.97623441854972	5.84654830534408	0.31854302720203
F	-3.03606530811254	2.14768573743615	-1.77820286180984
F	-4.03563890579971	4.62616666789135	-1.91665454194875
F	-0.92166412085649	-1.34767914565192	-0.41486472737504
F	-3.90955044896865	-3.22948872802471	-3.47649810176563
F	-1.43912266515189	-3.11497813326437	-2.35676803439384
F	-5.37319533170138	0.24761782293000	-0.71690807357288
F	-5.86916287579735	-1.54529077914846	-2.64107829368193
C	-1.94691444807791	0.15886634178207	1.87142499777904
C	-3.41692200256434	2.00872663973764	0.55926009766087
C	-3.12475289753518	-0.47031116579453	-0.47859274180475
C	-2.03523802784215	-1.10299199277720	2.46384952172604
C	-1.27027543492608	-1.47716956049472	3.55512141812648
C	-0.35343049498498	-0.57649663149783	4.08129625386064
C	-0.22146220327590	0.68633347839586	3.51860720452619
C	-1.01947917769674	1.03355521967998	2.44224916848609
C	-3.89884237066593	2.68674555664543	1.68149040059944
C	-4.43252781982126	3.96239486171442	1.61856647693400
C	-4.47755821892705	4.61929620683989	0.39582157171143
C	-4.00002091019023	3.98999856255116	-0.74639081947550
C	-3.49374865090046	2.70533619223300	-0.64911710514604
C	-4.37342173069359	-0.55865254169355	-1.09851874470488
C	-2.15728285111349	-1.36982186660707	-0.93263566101315
C	-4.65693610232628	-1.48029019306153	-2.09169014432186
C	-3.65896815374488	-2.34703813081088	-2.51769071041213
C	-2.39775613343928	-2.28906764082689	-1.93916094128694
B	-2.82948276136736	0.56590664198770	0.65022818849515

PyB(C_6F_5)$_3$

F	-0.55645117632132	-1.01583952427098	5.77946009476483
F	-0.25442954693506	1.58773726015515	5.02464264702014
F	-2.03598726946668	-2.12392789599599	1.48896487160444
F	-1.45509465269880	-2.84273572501384	3.96944208250180
F	-0.85782588313399	2.34885577428008	2.53815704989505
F	-3.66165865915172	1.96599965352421	2.49639212828099
F	-5.27518279247670	4.02060566235162	2.10561024140926
F	-5.36104502314645	5.29633444286111	-0.29632775595839
F	-2.12444622260050	2.35905445260329	-1.97476269014042
F	-3.75866844820576	4.43832480170268	-2.33286550876716
F	-1.04591313409898	-0.28385365774054	-2.43094718012255
F	-4.94899198948626	-2.78038269780925	-3.04660222311234
F	-2.54103411394323	-1.86296238069003	-3.93668346111625
F	-4.33187157405457	-0.47624517773509	0.98361694246724
F	-5.82507353069560	-2.06249515163933	-0.56573344397320
N	-0.31738237980852	1.01818624656559	-0.10819292842466
C	1.83813212756954	0.10730364434975	-0.54760946764788
C	-1.55728127801699	0.17422616506317	1.90531643472692
C	-2.76380146816385	2.07774049111827	0.29123669165991
C	-2.59669909832507	-0.33259301483575	-0.61534717270370
C	0.17783239741515	2.25285328954357	-0.25379158617014
C	1.50830206812531	2.47320166202184	-0.56071810074255
C	0.50401972015266	-0.03889897438111	-0.24119309266123
C	2.35431415622071	1.38725130186235	-0.71535089281291
C	-1.65155635384247	-1.14572715108231	2.33103895976584
C	-1.33443020883109	-1.55911203842342	3.61992024302085
C	-0.87161876455099	-0.63610108551093	4.54193777702033
C	-0.72103384396663	0.68761395155620	4.15465520987579
C	-1.05252962492198	1.05220599915838	2.86196669350856
C	-3.61656275926846	2.54605854253828	1.28872565893662
C	-4.48467757021427	3.61622414365203	1.10895980793528
C	-4.53541555993136	4.26737818521278	-0.11250533820803
C	-3.71875484638814	3.82994513738009	-1.14381423126294
C	-2.87434814160187	2.75434510574401	-0.92318397803689
C	-3.84589871918315	-0.81365951319387	-0.22412667096501
C	-2.21178952958686	-0.70947196253228	-1.89612061409458
C	-4.64250504093759	-1.62846124921342	-1.00830969561264
C	-4.20074994961928	-1.99547496020904	-2.27178437328985
C	-2.97671268874975	-1.52955441252114	-2.71743910404805
B	-1.84876569882988	0.72272553931742	0.38790658205818
H	2.45829062068200	-0.77111564662554	-0.65241894570185
H	0.05521096367715	-1.00914235108840	-0.09201910891166
H	-0.51150759008368	3.06624995427462	-0.10062066134548
H	1.86364296771592	3.48764047604891	-0.66963427545034
H	3.39911001167911	1.53199563162684	-0.95629601317134

IM0

Mo	0.02403939283581	1.40803717619177	-0.43260715375297
F	-1.13493502340753	-2.24492541594345	0.90644419977456
F	-1.29114662006836	-0.37316558562705	1.96864281976527
F	-2.97254376497736	-1.74332298221695	1.94144339760691
F	-1.77801962967499	-2.14838864012079	-1.71035825635647
F	-3.60890361733626	-2.59869258308547	-0.64573976253004
F	-3.57645241624450	-1.04114657915091	-2.15743967873844
F	4.76147893471040	0.31925008845601	-0.59425617115247
F	3.13754036188801	1.73343812553392	-0.84518354262453
F	3.89414477071846	1.30636082267488	1.12995298471249
F	2.65240661244208	-0.66641990290485	2.45522874204376
F	2.16806399511034	-2.41554977767123	1.28816661012086
F	4.19775823668902	-1.65113690383441	1.29721048059286
F	2.39282661304036	-0.40797588231171	-2.29316128774539
F	3.53786147650912	-2.02065294742203	-1.39139155047590
F	1.37702606104072	-1.98628151695211	-1.23113040070572
F	-3.90701003324465	1.26595972730025	-0.72850995082192
F	-4.84942522580772	-0.36434902607143	0.34277991953689
F	-3.55574044968689	1.01984016699687	1.38852327603246
O	-1.47992127423770	0.21520906674902	-0.64095818038451
O	1.32834509850875	0.17825932783733	0.33784126224244
O	0.17328511893334	2.43591568909643	-1.73165111616698
C	-1.77898853400212	3.84050070902294	0.44260137576823
C	2.90521524091082	-1.30039728216847	1.30843328485038
C	3.62138720746254	0.75694344144080	-0.05880498332063
C	2.47586535951091	-1.22967695729874	-1.23001313046783
C	2.54756921584611	-0.37996648543796	0.08778258918538
C	-2.87292911681962	-1.61259051457166	-1.17254858699950
C	-1.97164122029991	-1.25054476192845	1.20784261157326
C	-3.73095121171005	0.35651749323015	0.23194252786658
C	-2.48187407823299	-0.53778199383698	-0.09217669643126
C	-0.92760649925769	2.73074086936063	1.00717225296952
C	0.48942155467962	2.82647616085776	1.13070025312997
H	-2.67480556615745	3.45992954717702	-0.04703194570389
H	-2.10195879499345	4.49859723188898	1.25526682157906
H	-1.22083384665288	4.43738578164231	-0.27876940312458
H	-1.43126863314562	2.09916636419868	1.73159922150006
H	0.99725930028276	2.32113754078559	1.94376765666018
H	1.00493100483860	3.69354040811328	0.73231952807638

IM1

Mo	-0.60432575840850	2.91864364025535	0.27764078735612
F	3.69742617057609	0.92865888095941	-1.60703350147910
F	3.07159916330253	2.79845632424751	-0.70010808463153
F	3.80560544759527	1.19375133124941	0.54327034574566
F	1.30316939863593	1.96019214575381	-2.55632793655070
F	1.02546726456657	-0.17351114860676	-2.47654449842066
F	-0.44760633313854	1.14734504302934	-1.57503549078999
F	0.43394213951970	-1.10058283313770	0.02150538343866
F	2.53308886467707	-1.30521205607569	-0.49127153409110
F	1.95451739281443	-0.55828815734413	1.45542410845009
F	-1.04008458306577	-0.44510181431261	2.48504419611597
F	-3.03421807331557	-1.13898826959463	2.96717280885397
F	-2.30281435777206	0.73230178483321	3.78189615408469
F	-3.31082644170781	0.34754540272369	-0.87042224390781
F	-2.27579966303436	-1.22506882610063	0.18144279624008
F	-4.38380418863457	-0.89129314460208	0.54530569682543
F	-3.77735948856590	2.63770418817960	2.55454129356512
F	-4.79946645110776	1.95271011719217	0.78097430800632
F	-4.95582677035945	0.82314396606275	2.62331773513297
O	-1.87972974870584	1.59359980159873	1.02542277330859
O	0.98065737507559	1.69946421696903	0.55446463554960
O	0.06832779231385	3.97889972580401	-0.82986493425602
C	3.07848803397339	1.47180259184829	-0.54568620444248
C	0.90034477476141	0.95335457830599	-1.78613676327023
C	1.64386929633891	-0.54021747078164	0.15963360799353
C	-2.29497592775121	-0.05710309397903	2.69263621928455
C	-3.21341939560358	-0.28535339852978	0.30871365548477
C	-4.12213002900840	1.53898840640214	1.86631370535441
C	-2.52832077503738	5.97646181016416	-0.51489973823055
C	0.51017385802599	2.72831337762533	3.23671882438975
C	-2.74586756305939	3.55592301787224	-1.13472709782097
C	1.61256594827064	0.93527722929573	-0.37925436269943
C	-2.83754626175415	0.73615955814644	1.44528991394094
C	-2.93254278985703	4.56120262703556	-0.25858845240209
C	-0.87524348435230	4.41796877346814	1.91250798539877
C	0.32886773047996	3.73939751175250	2.14002481651699
H	0.97217546351173	3.22699066907042	4.09463267571178
H	-0.44074146331150	2.31341882230651	3.56456411367545
H	1.16472286922491	1.91586750486457	2.92825905671842
H	-1.74650816042824	4.17480698633242	2.50869731184549
H	-0.86035243007137	5.40759536792903	1.48228831556902
H	-3.39034629368372	6.52292283795712	-0.91086838707231
H	-2.22659267953982	6.49323111249965	0.39603093283271
H	-1.72168415736654	6.03504133776219	-1.24475976999919
H	-3.23556148589271	2.60441921535097	-1.01041638035750
H	-2.23638647800018	3.73211606344030	-2.07423401894727
H	1.24283618521307	4.19604584807076	1.77636375872711
H	-3.53120345234329	4.37307994870596	0.62543543725188

IM2A

Mo	-0.00195421953814	1.35732024892574	-0.29197032730206
F	-1.93558525036330	-0.99048393829336	-2.29865482198364
F	-0.93648246578766	-2.33915907298755	-0.94260629700440
F	-3.05547154490799	-2.57706494623403	-1.32284618354728
F	-2.98999145980626	1.24691094225200	-1.20761076791206
F	-4.51697481205652	-0.27660587220921	-0.99068558615793
F	-3.96253759099599	0.96224439179232	0.69809847955254
F	4.91527159810458	0.25369190206430	0.33260804151068
F	3.68590868869184	1.62382856105407	-0.81175579888511
F	3.42313592204605	1.46321200686912	1.32719809744862
F	1.43261412367117	-0.23917925877505	2.05012047457678
F	1.58139608204075	-2.18395196083225	1.12642911457287
F	3.32091997624199	-1.30700579018616	2.07631011216304
F	3.75575476065264	-0.81179436598370	-2.07028677848770
F	4.07037459863861	-2.22296749285320	-0.45139174323518
F	2.18154060576747	-2.17727230531909	-1.50692947052130
F	-2.70778486104999	-0.69638961800228	2.38696099979010
F	-4.04513645822965	-1.93933423524657	1.21695854790310
F	-1.97951894354284	-2.53016116672530	1.51291356863645
O	-1.21365642820653	-0.01914311615375	0.31908113185915
O	1.47924742517852	0.15933253599300	-0.60418089634861
O	-0.05538798890437	2.31900392518316	-1.64826195539779
C	-1.27996584814522	4.79826351011679	-0.14063903627808
C	2.24186572174098	-1.04341487503778	1.33601141111083
C	3.68732342893079	0.76771192596528	0.20975507826294
C	3.17028132118358	-1.42092226355381	-1.03278491542704
C	2.60847444197242	-0.35319482793187	-0.02354887818334
C	-3.49513517920721	0.31939814638370	-0.37578247291437
C	-2.07676096194237	-1.68548907782409	-1.15623360474464
C	-2.78897661555714	-1.48626975720081	1.31400354905296
C	-2.36529730328744	-0.69522771672650	0.02697629794104
C	-1.29496547759391	3.87075815530246	1.02581591940899
C	-0.74733990294512	2.65862259457052	1.15726017054280

H	-2.29889220908121	4.95800231259332	-0.50731240614671
H	-0.90667519926627	5.77765080588892	0.17199254809821
H	-0.67078469134786	4.43485936100339	-0.96333150028509
H	1.15140762328433	2.08759110872284	0.66003914081444
H	-0.79062714548233	2.17210777654608	2.12999810509266
H	-1.83621376090040	4.24390144684936	1.89552265242411

IM2B

Mo	0.10152473551566	1.60790618513213	0.15637378813578
F	3.76987609730587	1.31571979803320	0.96805803096011
F	2.49150792454454	0.16667632356676	2.28149951079327
F	4.32341070717683	-0.69830656266732	1.51470262853146
F	-2.88568550359486	-2.83462290129853	-0.56358000860828
F	-1.16540827750508	-1.92631961523777	-1.51450149336540
F	-1.10930524921752	-2.38877313452446	0.59656031791209
F	4.49845138396143	-0.20844867261353	-1.30706048201427
F	2.69756310228333	-0.73214039866494	-2.39316642562395
F	3.58057516388652	-2.59922434077642	-0.21405907801765
F	1.49993391915841	-2.50608344970160	-0.78767393845664
F	2.93472739164302	1.26394359297658	-1.61221122185830
F	2.03712422463013	-2.39690501313067	1.30124366985751
F	-3.65650934995004	0.67607353346322	1.60798757883308
F	-1.95479880139015	-0.47631793604655	2.26902492663849
F	-3.83361336469793	1.17246794494675	-1.04117699741047
F	-3.23402133717697	-0.44777285409675	-2.33073687744101
F	-4.72975651248187	-0.78409634059211	-0.79823741877107
F	-3.77816240116467	-1.48887831455937	1.69015279528647
O	1.18234586065630	-0.00084800268557	-0.02391199120698
O	1.32831019903084	2.60665060897859	0.71491171576371
O	-1.43115254766785	0.38761706979884	-0.21830802126595
C	3.17506016821491	-0.03251757846033	-1.35805706740173
C	2.40144560381508	-2.02767504080401	0.06641091590403
C	-3.58799776034592	-0.15022360630764	-1.07491178940051
C	3.28716423237844	0.09220070717762	1.21033111012641
C	2.47181877862484	-0.45896709601013	-0.02260194334279
C	-1.75226435941731	3.32440884199631	0.37303269062675
C	-0.83035893352866	3.68134664149614	-0.58085224246337
C	-0.42605312114406	2.79398584436677	-1.62291392421950
C	-2.97163054641183	-0.46467467434813	1.40257217593473
C	-2.43233756674574	-0.51153131414805	-0.07191280790045
C	-1.89027577395888	-1.95128468986506	-0.39682874278689
H	-1.17097377120799	2.16098748223508	-2.09305553111413
H	0.39920095005831	3.08937048684511	-2.25738205433505
H	-0.16823396139422	4.50944737618049	-0.35620659274632
H	-1.87313672752318	3.93503953323880	1.25617276436862
H	-2.52498514035189	2.59943788893432	0.17289413754301
H	-0.70987943600787	1.86794167717220	1.59251789253521

IM2C-1

Mo	0.06060888879757	1.07391130481187	-0.86174904773780
F	-3.36136596916807	-1.49570368849936	2.13005917543986
F	-3.24891226650582	0.64267946255036	1.83675236259112
F	-1.43500571870424	-0.49956149718218	2.10542120096814
F	-4.92622868615558	-0.60157364385432	-0.10006710464076
F	-3.85634303366201	-0.64825000355235	-1.98664690491404
F	-3.80515659841503	1.13050272801149	-0.76611141054590
F	-1.42835642878169	-2.78870858520321	0.53486416045548
F	-3.46807874204054	-2.94886649970905	-0.18788017659204
F	-1.86587244287568	-2.43323810422235	-1.54900253931149
F	1.53753513707559	-0.00035669728349	2.00210962484606
F	3.09376883079662	-1.49417631375188	2.24447020249419
F	1.10252839378582	-2.08052696526119	1.62972603406267
F	1.30452082724277	-2.80953096148119	-0.96015963389580
F	3.20409416060291	-3.21529628802550	-0.00140343995754
F	3.13891985955336	-2.18548429639673	-1.91143471876978
F	3.89103997096248	0.79166536579820	0.80657765963002
F	3.94429106945367	0.36689951387684	-1.31114307918593
F	4.83490836753551	-1.02080113600970	0.09426692331406
O	-0.04228772098947	1.69727715180009	-2.39959840374717
O	1.46223223751298	-0.21808322608734	-0.65665547565851
O	-1.43671382193688	-0.08539964205881	-0.51091735514887
C	1.46785236126832	2.53582557136921	-0.13625202030204
C	0.91333993244572	3.94600909031902	-0.11550717978840
C	-0.36275182594428	3.82652520028501	0.72050221070135
C	-2.52333796960746	-0.73234702435737	-0.00284300679154
C	2.42716221321068	-0.93236557076781	-0.00392476417101
C	-1.07270453454677	2.55967506960912	0.25733387886386
C	1.91649170387540	4.96886443433420	0.41245050462442
C	-1.27064345988327	5.05287823572607	0.67099751492723
C	-2.65215922327927	-0.52490059937889	1.55047944464349
C	-3.81206926114001	-0.20669595462424	-0.72612928170378
C	-2.32489500659422	-2.26561150879489	-0.30980432959049
C	2.04186370998747	-1.14181262843955	1.50207329002202
C	2.52403817702803	-2.32387310604652	-0.73135951873585
C	3.80941453329432	-0.18832398200305	-0.10574965276735
H	-0.04827022170146	3.67165462370985	1.75979996728024
H	0.62821163592328	4.22657169939048	-1.13611557608448
H	2.31217504928300	2.40067082359753	-0.81933670331551
H	1.77723670305456	2.23064203148069	0.86544323071630
H	-1.84358908461947	2.77005200150343	-0.49177846217232
H	-1.55242965974564	2.03453038174151	1.07865829764865
H	2.82103242454015	4.98355792031997	-0.19859404201038
H	2.20843120272493	4.72942404422339	1.43872428643575
H	1.49738821340131	5.97683221079382	0.40643480624893
H	-1.58220681847802	5.25805001925260	-0.35673814166294
H	-0.76508024212961	5.94066948316335	1.05526865937886
H	-2.17079688445197	4.89900955532278	1.26933853390903

IM2C-2

Mo	-0.09034116721212	1.21114204466703	-0.56460118614556
F	4.80631142495822	-0.04995180670049	-0.57971908111363
F	3.33472569109510	1.47587845475444	-1.01076246332180
F	3.90733226407272	1.08517059328499	1.03458896692198
F	2.48179790918767	-0.60030421353102	-2.41949358906925
F	3.42794570236161	-2.28227015380317	-1.42480775822216
F	1.27320457012161	-2.05965856164479	-1.39416983387183
F	1.95208194039750	-2.53823679025363	1.16837640493199
F	4.02254651596839	-1.90523450032463	1.26308518776990
F	2.49735127571212	-0.81336248300842	2.34556280878842
F	-1.55450032756242	-2.56294518260489	-1.60115371182629
F	-2.87428122497208	-3.34075801354277	-0.07458966288680
F	-0.84255619457104	-2.76722474707374	0.42870229737557
F	-4.05844800734715	0.58252256509909	-0.63298629176862
F	-3.86258398292082	-1.18384148835077	-1.85641854238200
F	-4.74020752312235	-1.33155416597331	0.12178053368833
F	-1.15325556547448	-0.46323372770838	1.98182760684843
F	-3.19563162246265	0.23830196875095	1.90772510891836
F	-2.79662321291172	-1.86188634378913	2.20645876696627
O	-1.46840508875128	-0.14404029700572	-0.61583916419244
O	1.31914616608351	0.10386060771110	0.18353931529310
O	0.15867322976183	1.92517402857837	-2.04598267494111
C	3.67217208149295	0.50595885966590	-0.14971788598889
C	2.43050493045688	-1.39838848923338	-1.34178935374405
C	2.75712651621235	-1.47255791534187	1.21337767456282
C	-1.90514527892022	-2.45215458930923	-0.32192291332759
C	-3.79071527710148	-0.72740155345041	-0.60308286335553
C	-2.38812734507013	-0.77360551406531	1.55561491976224
C	-2.03117717964884	4.96394053968047	-0.50037992771517
C	0.73496789161292	2.42157321705275	2.16086175425752
C	-1.67251707985613	2.52831264927110	0.13766765594947
C	2.50535192466962	-0.53902246842859	-0.02736570654019
C	-2.36375587024496	-0.97872683938868	-0.00457038450603
C	-1.30833456526872	3.98783297989125	0.42392483934030
C	0.21765935221231	4.10151479780923	0.31213035664252
C	0.81414587442341	2.75865272262525	0.67954154268752
H	1.27659459486557	3.18107116297753	2.73469582200042
H	-0.29222638491153	2.41438738466967	2.52848523206311
H	1.18061886038646	1.45499706976050	2.38769137792890
H	0.61141150720347	4.90091138223505	0.95100566590725
H	0.48658176776950	4.34948966591478	-0.71738523656264
H	-3.11389649930700	4.90628628989441	-0.37340601524712
H	-1.72281242317176	5.99257368850808	-0.30009702003292
H	-1.79958771272439	4.74201431202860	-1.54546695795053
H	-2.13239547848941	2.03928585214007	0.99129944510600
H	-2.35462930728579	2.41985647925059	-0.71063410016164
H	1.84878418792021	2.68535060526292	0.33608581501277
H	-1.59889186143743	4.21353992504813	1.45351322615059

IM2C-3

Mo	-0.06603654533228	1.15126003773787	-0.66833377950429
F	4.81654993555987	-0.12928347616142	-0.67129365883929
F	3.32765966896492	1.34704561012139	-1.20282920198883
F	3.94033896264653	1.13361083198710	0.85771769867989
F	2.44206718032502	-0.83846276948042	-2.41194219034951
F	3.44238072770511	-2.41973421920630	-1.30949997121590
F	1.28594859054415	-2.22719381923783	-1.23835123329684
F	2.01797700606413	-2.48099989458221	1.32735850872341
F	4.08103635031641	-1.81865210153316	1.33675903091121
F	2.55867265970633	-0.65979190665517	2.35154514646207
F	-1.47846167171058	-2.74794790070404	-1.25108927688000
F	-2.84121991264130	-3.29938110077215	0.33447328212636
F	-0.83661796639924	-2.61291578821265	0.80811246082950
F	-4.03609778718335	0.46859464818811	-0.90977180509932
F	-3.76960947283882	-1.46946734713029	-1.81854855307137
F	-4.74325558258099	-1.30757621733985	0.11251112198846
F	-1.26334106788886	-0.13323518107590	1.99617456743416
F	-3.29667294031835	0.54038760297870	1.69875151877410
F	-2.92230119739768	-1.48664937675055	2.34050095975636
O	-1.44905298556725	-0.19636874205031	-0.63635653487586
O	1.34195901219771	0.07467805499696	0.13793316002173
O	0.13583432680450	1.78724121326214	-2.19190476012791
C	-2.12579243999592	4.96321815563266	0.46317640669406
C	3.68701993719599	0.45506256861061	-0.26867439142284

C	2.43222970825821	-1.54701243891050	-1.27174504149682
C	2.80964992598694	-1.40557762821371	1.27255425721941
C	-1.87599708232090	-2.44273573808140	-0.01843726360084
C	-3.76225319043758	-0.81572734777160	-0.65425353559048
C	-2.47641704096813	-0.50836363207411	1.55356440148034
C	0.85896571820182	2.50660785125727	1.96840069246098
C	-1.60136593459208	2.53048962269427	-0.00568415196030
C	2.52798465117448	-0.57994806232251	-0.03646554776443
C	-2.36608135311143	-0.94774769989204	0.04772980503291
C	-1.21576410641366	3.97767036164307	-0.26951664554683
C	0.28016372977451	4.13322164021523	0.09210209366512
C	0.85867529255727	2.77591983863662	0.47077007416980
H	-1.85199380592125	5.99504300261818	0.23099264946928
H	-3.17121109425602	4.82214593728105	0.18089872544324
H	-2.04715028334426	4.83029023577213	1.54560066086724
H	-1.32136557630819	4.15018554783064	-1.34332058364333
H	1.50478235393084	3.23973277100317	2.46287803376302
H	-0.13573276019556	2.61737437084089	2.40308682861321
H	1.23353398347125	1.51622085765065	2.21833085957809
H	0.40887329246344	4.83316296333034	0.92373335218614
H	0.82467668850690	4.54786867254612	-0.75720336997787
H	-1.79225814784957	2.34440847896846	1.05007015853493
H	-2.48167331635600	2.22321516550513	-0.57148057684531
H	1.87271355957292	2.66186634684932	0.07910561821313

IM2C-4

Mo	-0.03487144676064	1.20052073169895	-0.44229140778595
F	-4.72432934982676	-1.29330529606157	0.70695166450525
F	-4.09471522521410	0.77740219854272	0.82146095637287
F	-3.34642428138272	-0.65071647080499	2.25440353418406
F	-3.48801500765171	-2.14308095394622	-1.68314243471254
F	-1.91814858260178	-0.77116244985334	-2.28366734576243
F	-3.86953238766323	-0.01259831407114	-1.75473930958294
F	-1.02563172455753	-1.85642783583571	1.60055700442703
F	-2.60268492413930	-3.08159240282605	0.76011480255093
F	-0.89630251105878	-2.51324512188279	-0.45289157706863
F	2.26817748700276	-1.20797147651397	2.39627185989025
F	3.98354287134515	-2.06132370307267	1.39025977111913
F	1.98290257984956	-2.73850813360637	0.89987668764637
F	1.59290580223947	-1.81940792627346	-1.66026501892390
F	3.71395232380185	-2.12318281416983	-1.34009564119188
F	2.99860900847219	-0.28612535489009	-2.23698182540318
F	3.79001491199719	0.88872255830392	1.59520361270320
F	3.41785068821727	1.62037198199792	-0.40333057275853
F	4.89661440489887	0.07315096888246	-0.08049133819712
O	0.60360496205222	1.39540198472828	-1.97081169201230
O	1.33201877096563	-0.02470435872269	0.25829064335650
O	-1.60185595868596	0.26408820327449	0.14498558790091
C	0.73743826302334	2.42484543943084	1.23129435487433
C	0.33737265774252	3.89227785425298	1.21527960733793
C	-1.06660189316041	3.96599255396473	0.63306864962749
C	-2.46996596731328	-0.78560407054779	0.03275038250257
C	2.57586051312085	-0.55694791564183	0.11602322106932
C	-0.96537399192924	3.17224638471629	-0.64295859649200
C	0.13022412057472	1.67899473173456	2.42881178344116
C	-3.69083790176892	-0.48859863308879	0.97267316621371
C	-2.95498011953552	-0.94320047636479	-1.45158393118460
C	-1.73735754025663	-2.09652157453511	0.49949997551531
C	2.71570804576014	-1.66654849970507	1.22354194542671
C	2.73050588846427	-1.21425469608463	-1.31093504718373
C	3.69934918286584	0.52125337873319	0.30781438419573
C	-2.25349570151518	2.84643409040456	-1.38942490851433
H	1.81510885194962	2.30691574941595	1.23860280284941
H	1.02137986957092	4.45702541358517	0.57652887446367
H	0.40359121638307	4.32584514014302	2.21870689952476
H	0.36355596913714	0.61672728239878	2.43654772824698
H	-0.95329630685927	1.79342192445157	2.48441327729905
H	0.54753237379155	2.10824060080800	3.34545076776222
H	-1.38693649005099	4.99630606255037	0.44278992626447
H	-1.79892099396459	3.52679856507292	1.31539926580199
H	-0.26450936769020	3.67407248502991	-1.31097117676063
H	-3.03326094046884	2.49228899138094	-0.71863899964908
H	-2.12030202491299	2.10739108327400	-2.18533758799223
H	-2.61141012425755	3.75361211972234	-1.88972472049737

IM2C-5

Mo	-0.05192868355021	1.24434121406059	-0.57799268855184
F	-2.75668211309891	-1.78313576606881	2.22567130103124
F	-1.27457808748220	-0.21224897528150	2.01884359618442
F	-0.86822092900323	-2.18882784241884	1.24908353069155
F	-4.77885630014113	-0.98095332534735	0.51295934550793
F	-4.13744453750635	0.59633211983725	-0.82147484047575
F	-3.72046569232509	0.73968245055399	1.29353385184379
F	-1.46543221283511	-2.51724631130030	-1.36783339893743
F	-3.21945579095019	-3.09032381939568	-0.23527927691801
F	-3.40336206607071	-1.76119523115345	-1.94194580294829
F	2.84191521241995	-0.28165399750924	-2.33448535852635
F	4.62454661878459	-0.33030448307914	-1.09816555466145
F	3.31450747787170	1.38818587168090	-1.05236209045805
F	3.72308533569593	0.76752624110579	1.54893657336861
F	4.29979040511362	-1.31993367154409	1.44448402516072
F	2.43964431029763	-0.74995270614090	2.39385583358971
F	1.31410864222851	-2.23411987985444	-1.26793368855976
F	1.84195584362096	-2.78683859597375	0.75234450903974
F	3.36911286699528	-2.70980271691669	-0.77966275781602
O	0.45061214595171	1.88575179607315	-2.02636361010286
O	1.27417911424385	0.00179576839799	0.12816171037453
O	-1.54381120357160	0.00218883989214	-0.61260571548975
C	0.94545796049214	2.61681529267911	0.80480193009912
C	0.57171444042511	4.04776847496227	0.46785637715458
C	-0.95081847140395	4.12760477557299	0.50596350281455
C	-2.41880486414662	-0.83145104754703	0.02917346629600
C	2.49379964789401	-0.59821102538859	0.01034142788604
C	-1.49780913849151	2.82875937692575	-0.08459118273472
C	0.65440002576689	2.23314859353794	2.25050024006176
C	-2.22938963654355	3.01795402247574	-1.41694764784631
C	-1.82804560592284	-1.27849676366336	1.41103378798096
C	-3.79806671210331	-0.11234556881478	0.25579378829604
C	-2.63209614528346	-2.08471274292148	-0.89612166260213
C	3.34576153965724	0.05260124752781	-1.14062954654398
C	3.26078914293238	-0.48124865350881	1.37618323814646
C	2.25488162489649	-2.11971411019501	-0.32977906857091
H	1.99490507932002	2.42797091635198	0.58172907005255
H	-2.14799485315127	2.32353015101132	0.62856495669724
H	0.93454644626986	4.29468248650184	-0.53201434149293
H	1.03940321854700	4.75282835564064	1.16570527931443
H	0.94635488674337	1.21006118835453	2.47341034928151
H	-0.39883397392773	2.34538589987447	2.51075478527104
H	1.22230926386044	2.89074360131964	2.91750213808085
H	-1.31943081778797	4.99312080402680	-0.04889994121313
H	-1.29579343140847	4.24735289067715	1.53424579100901
H	-1.64875716359050	3.65223613346461	-2.08674035662658
H	-3.19314356498271	3.50280984703032	-1.22701692322479
H	-2.45172925475003	2.08808887448652	-1.94049895093338

IM2C-6

Mo	0.01155266295362	1.19489790727935	-0.59283620294749
F	4.82434477487820	-0.63125253461744	-0.44849395078047
F	3.38783628889991	-0.76135074258016	-2.06945473141063
F	3.56697368906849	1.06491645426601	-0.93550118229634
F	1.54901610475435	-2.54283390906825	-1.16005875618311
F	3.42766443264936	-2.97803648839624	-0.17894993638067
F	1.60192556490003	-2.83752350645835	0.98040675443319
F	1.86558127512614	-0.51719312486755	2.43497511825727
F	3.78575427186553	-1.45488947069141	2.06879317768536
F	3.54314068155824	0.66976290816371	1.76450724420661
F	-1.80199543496207	-2.33958091088688	-1.74710422354171
F	-3.12831216006668	-3.07693412113692	-0.20352004085415
F	-1.04953341897261	-2.65623874580692	0.25234087831622
F	-4.06993484765715	0.92794877846244	-0.57743602540425
F	-4.04253520640293	-0.80338455721502	-1.86533027988032
F	-4.84082554742985	-0.96407835520374	0.14597098455761
F	-1.12027487110446	-0.36899981097035	1.85417508681400
F	-3.11147973565474	0.46731889887226	1.89359370386922
F	-2.84226635257414	-1.66169129813942	2.13297888176784
O	-1.53934111983700	0.04099094734638	-0.70723016647664
O	1.31099909388441	-0.15838787823581	-0.14108344968645
O	0.46043125395332	1.93429646541125	-2.01137367733530
C	3.59923692129533	-0.27042324958302	-0.84407310425433
C	2.26716177859173	-2.32242201296499	-0.05986085865570
C	2.93573291619999	-0.51905299086848	1.63495380730792
C	-2.10929708065354	-2.24781785519899	-0.45524613132262
C	-3.88731043681659	-0.39672980882383	-0.60261991437689
C	-2.39502050264450	-0.58946806648325	1.48373159428627
C	-2.04947206998532	3.28159991820802	-1.14004296982474
C	-1.38244921559542	2.75128785216983	0.13261432536596
C	2.49118167107743	-0.77508167632543	0.14702790764926
C	-2.45540656920019	-0.75875572704790	-0.07958973429074
C	-0.82166895701902	3.82604912507888	1.05906392214494
C	0.70032164827317	3.84847490297551	0.93404273990267
C	1.09751293176762	2.38649815814901	0.84880451839981
C	1.20751921043234	4.67125031030907	-0.24879792880996
H	-2.35274534851559	2.49337081985780	-1.82991746120302
H	-2.96081875856437	3.82246059451142	-0.86263313157945
H	-1.39449607924686	3.96681828004225	-1.67570522851800
H	-1.08835654416989	3.58353940658835	2.09053822877168
H	-1.25977304851720	4.80433561338958	0.84050584084737
H	1.13675246991927	4.28002929622447	1.84383027106494
H	-2.10799389963826	2.14732791675894	0.67265836750590
H	2.14690229952668	2.24966162501200	0.59542766322157
H	0.89887990479569	1.86580579979095	1.79114069030673
H	0.82932532305971	4.29745978751052	-1.19995878306991

H	2.29822510435672	4.64267344354654	-0.29794744312330
H	0.90320493144110	5.71499163164573	-0.14772639447616

IM2C-7

Mo	-0.03023421111620	1.17620707331728	-0.81085836476693
F	-2.74712224611042	-1.51897394745280	2.29629672611637
F	-3.03342317084133	0.59432601224197	1.96520194528133
F	-1.04391084606253	-0.24250445381395	1.86929452981877
F	-4.84588029179606	-0.89071624712444	0.37386898269242
F	-4.14170868670488	-0.85162589344049	-1.67837279512546
F	-4.08992083711666	0.94819541250821	-0.48856957355211
F	-1.07757896096451	-2.63146601463224	0.39624263124989
F	-3.17954251146128	-3.04043969292485	0.05002849326897
F	-1.90966043866453	-2.40522131290027	-1.58365523539895
F	1.85426747126503	-0.18839128329605	2.19194885272988
F	3.45821884066502	-1.63323061990610	1.98788558903794
F	1.40625939439606	-2.22065272559973	1.61947780916876
F	1.32608012641911	-2.67963507060437	-1.06559245407369
F	3.36573073038752	-3.01911845798691	-0.42761857126350
F	2.95154263107408	-1.78164196122636	-2.16303516386289
F	3.89235195519188	0.90716538933736	0.77254611523645
F	3.65099553661065	0.73646288630988	-1.36855775876521
F	4.82675969917383	-0.72797514806042	-0.28837903915816
O	0.19109131208883	1.84457251734523	-2.31586361897014
O	1.32314217826011	-0.14220662879887	-0.44885848249266
O	-1.57852927250585	0.02161788021670	-0.68834439436752
C	1.65330641445071	4.82590181792215	1.25922188741243
C	1.25347543899695	2.51347206035071	0.29253833643564
C	0.76577198037897	3.94534177687758	0.38256718639961
C	-0.66294920914200	3.80898460852679	0.88610129216259
C	-2.47122925066450	-0.72943747922537	0.02514187672426
C	2.42911833516349	-0.82507089411051	-0.03500642623374
C	-1.35144205235431	2.74576856226135	0.04675202425974
C	-2.10759915214438	3.31201779060899	-1.15858497139007
C	-2.33385446407094	-0.48016191359087	1.57293151769271
C	-3.92295139315746	-0.37806046745737	-0.44860045704985
C	-2.16009874535219	-2.24224905086132	-0.28624781687802
C	2.29113623874396	-1.23118376336565	1.47312728320335
C	2.52454529668201	-2.11320401566909	-0.93770356306653
C	3.73246229309588	0.03606441250421	-0.23434021827239
H	2.67366390886519	4.86762609160960	0.87313670430833
H	1.26974764618486	5.84774607446599	1.30471206687368
H	1.69603447877863	4.43583045254548	2.27972735067515
H	0.74347260155196	4.36927212359944	-0.62707107424459
H	1.35021366790664	2.07320838006367	1.28911816502081
H	2.21090481771071	2.42133449690132	-0.22130149374134
H	-2.04450075852315	2.17348586242672	0.65664725181436
H	-1.20813594505196	4.75791207586731	0.85198002004209
H	-0.63437562303936	3.49620213121609	1.93468751260090
H	-1.47099242435847	3.96754356003068	-1.75278242516902
H	-2.96223568898134	3.89411356737787	-0.79738479835183
H	-2.50531681385773	2.54147402561543	-1.81868345403181

IM2C-8

Mo	0.03509674540960	1.31921425761311	-0.67637207058969
F	4.80291489836451	-1.01820313507966	0.17596351328781
F	4.06555433750383	0.43245752058797	-1.24745387324073
F	3.95230307070364	0.84974324035464	0.87046365787603
F	3.06672235445170	-1.94715259073490	-1.98350984966027
F	3.03616566691581	-3.08929870476504	-0.13914103844523
F	1.18198101328656	-2.50030837030980	-1.09363474531558
F	1.04734913728067	-1.97218125874703	1.55691988314873
F	3.04233018719892	-1.46549778939929	2.21946668205991
F	1.52485049031865	0.07461083897890	2.04342733873812
F	-1.67582851876315	-2.20401757460636	-1.72580592734813
F	-3.29550763462044	-2.93163093995406	-0.49045404244393
F	-1.29598768128697	-2.75907677154568	0.32703218513872
F	-3.86612291349099	1.14163143532470	-0.62439425149502
F	-3.74638603485217	-0.47686716630901	-2.04550129799471
F	-4.86867393339532	-0.73109200829138	-0.20554195285950
F	-1.44883486858536	-0.61377924539162	2.11568431830468
F	-3.33623096640039	0.41730547138234	1.91202276714119
F	-3.29706990512142	-1.74090212219166	1.97796351362965
O	-1.40292696271889	0.04670334847619	-0.44957416339913
O	1.50448932052167	0.06288982892159	-0.61116376525765
O	-0.15460621102824	2.07574892944416	-2.14291060453532
C	3.84271409823451	-0.11875459331248	-0.05435820959465
C	2.42569284039632	-2.11203441562481	-0.82094851754938
C	2.00838739265256	-1.05118068663482	1.48500451217474
C	-2.18340779549247	-2.18800549972659	-0.49458170434441
C	-3.77008528406324	-0.18689891540515	-0.74100739327701
C	-2.64649145168394	-0.66735322463288	1.52347051856199
C	-1.97057632783357	3.58820812071820	-0.27185625335404
C	-1.18363663931918	2.63225564502339	0.63141935156483
C	2.41066366303722	-0.76490175091506	-0.00686852585714
C	-2.46594818526870	-0.70335381625943	-0.03972303988610
C	-0.36472424057108	3.36545998830216	1.68059174485122
C	0.83426215142779	3.94464761174836	0.95785151690901
C	1.43447678825277	2.79119438420535	0.18053107344016
H	-2.53134183436614	3.06903844960025	-1.04491903380407
H	-2.68923501026351	4.14208583065892	0.34250177799275
H	-1.32141368632618	4.31065964404721	-0.76410306306824
H	-0.02837481025531	2.66660695835477	2.45207695223446
H	-0.96871922499146	4.12888132522351	2.18399025386587
H	1.56869888420438	4.38176225353962	1.64244297068489
H	0.52497924511594	4.74105142962093	0.27776666502230
H	-1.88238238505804	1.95162172199089	1.10624851237450
C	2.37073675119343	3.21382144099225	-0.94818470797521
H	1.95855373888591	2.14858005402079	0.88927557142409
H	1.87473029348413	3.92977991656575	-1.60429044812991
H	2.72253167687924	2.38694776391462	-1.56200075295323
H	3.25818776453637	3.69579317022611	-0.52248604804739

IM2C-9

Mo	-0.00959162388473	1.14304522542121	-0.75466192902065
F	4.93182953484516	-0.51941661094278	0.23635799392955
F	3.95749708067499	0.86490166980699	-1.11591771194470
F	3.78218250190535	1.13762846631428	1.02061586274291
F	3.43591055797911	-1.70534383141821	-1.91366545053380
F	3.52868570249671	-2.85435465363194	-0.07434727440330
F	1.63636066525941	-2.56628349153533	-1.08867293886794
F	1.26821127928611	-2.04229628542960	1.53407804013524
F	3.17211610983088	-1.32014573868662	2.26901185300977
F	1.49001164199441	0.03699926774760	2.06667253929946
F	-1.54647728748693	-2.48536787452533	-1.69792216306924
F	-3.14070639692344	-3.23839995919374	-0.44277285791491
F	-1.15102003132904	-2.94927028443492	0.37289261775747
F	-3.83232628860411	0.82548043967121	-0.75330460189183
F	-3.67510753880210	-0.85744126267375	-2.09432724856450
F	-4.81095550759611	-1.04421447838831	-0.25559318341424
F	-1.42148315747560	-0.78956880971542	2.09395536334378
F	-3.32112546546154	0.20485649380724	1.83184346090686
F	-3.24950537213231	-1.95070390507128	1.97201739244367
O	-1.37101520222720	-0.19028812618430	-0.48475010884201
O	1.52363965801583	0.00051944808669	-0.60300198035717
O	-0.17887338093419	1.81019164479369	-2.26736207585820
C	3.83526084980394	0.21866838613428	0.04431901427830
C	2.79251880380844	-1.98338232891143	-0.77428781031065
C	2.11765917144859	-1.01411805948515	1.51013536081315
C	-2.06180516527326	-2.44864425106445	-0.47013807936414
C	-3.71681495156743	-0.50628263295680	-0.80557663499546
C	-2.61116441518889	-0.88203909610056	1.49012036483668
C	-1.30477174667971	2.43631640884494	0.43203264318628
C	2.53203393366882	-0.66220528274572	0.03882326794260
C	-2.41118663317060	-0.96493468460181	-0.06736822627914
C	-0.69818819683926	3.66864676860708	1.09386173188325
C	0.54680950666509	4.04358759932375	0.27830158837958
C	1.23411240461862	2.70615090251798	0.07263543556948
C	-1.71445010431321	4.78963477535597	1.30106655911573
C	0.25127901278342	4.78093902307377	-1.02588756664688
H	-0.33804726454771	3.35875841945285	2.08105600048823
H	1.19536635890828	4.68888538865957	0.88453123170464
H	-1.81234715781061	1.80280091334749	1.15332075503620
H	-2.03588206752541	2.69805075966325	-0.33983241348269
H	2.06791862716803	2.73269662098626	-0.63667686239457
H	1.59592968705195	2.31416919074973	1.02363711662284
H	-2.14624382142942	5.11345874506608	0.35226869606014
H	-1.25267230068305	5.65775073180553	1.77731339760797
H	-2.53355941502809	4.45403664693757	1.94005098486359
H	-0.47390388480599	4.25146204133872	-1.64451711287915
H	1.16374501428024	4.89060114339523	-1.61549120097531
H	-0.13739372477339	5.78074452678838	-0.82958383994692

IM2C-10

Mo	0.06675000985604	1.05347614940735	-0.96981811120164
F	-3.43634104167619	-1.22316408911449	2.14073616131385
F	-3.25334106407791	0.89222121505648	1.73813583313640
F	-1.47731775246643	-0.29680238826398	2.05311635863108
F	-4.98629183313330	-0.37982067554461	-0.12044095538638
F	-3.93750266188997	-0.57025143949964	-2.00956699978843
F	-3.80530792430017	1.26855372718714	-0.88815821681067
F	-1.56600685980557	-2.66370398903873	0.60125010824046
F	-3.61747344789065	-2.78490459144043	-0.09578020222330
F	-2.01085626182794	-2.40097992822944	-1.49475424179910
F	1.49804962258783	0.06733214409464	1.98297068160910
F	2.99194835046836	-1.47553614695639	2.29828950315690
F	0.97720451413851	-2.00893566259287	1.71181564937966
F	1.15459798782937	-2.87985789282934	-0.83721986757856
F	3.03782096879488	-3.30554374916697	0.14439490478562
F	3.01054708101994	-2.37128911030257	-1.81511677470509

F	3.87711156074558	0.70343938988715	0.75440639482813
F	3.90561371460262	0.18210985325165	-1.34243182861682
F	4.74889790525825	-1.17326072079899	0.12238893485176
O	0.02758171148891	1.58435461439268	-2.54492270771459
O	1.40585566783694	-0.28416936327520	-0.66341542936479
O	-1.48377493859846	-0.02010132562495	-0.58117951307527
C	2.07561655994814	4.93423810671039	0.12206544558432
C	-0.05818433958650	3.85722862904555	2.00720072463002
C	1.52833648323154	2.48631491437331	-0.29786588886937
C	1.02816818663256	3.91719958141755	-0.32755423655211
C	-0.27243980241990	3.89362435213902	0.49265834740002
C	-2.58981510484600	-0.59903627625139	-0.03373058544524
C	2.34520111422832	-0.99834057788365	0.02512629095779
C	-1.03310973071801	2.65796770346975	0.02273881244341
C	-2.69898428187571	-0.30839184516533	1.50793604564527
C	-3.86374075877855	-0.06303224528736	-0.77540497630034
C	-2.45094138435876	-2.15222297927863	-0.26249791613840
C	1.95395337907744	-1.11675749064728	1.53925525942307
C	2.39079714629762	-2.42775377074428	-0.63070265658470
C	3.75468586226217	-0.31261568749518	-0.11289615479663
H	2.92453413918444	4.94106541719311	-0.56423111042294
H	1.65747399485460	5.94363933283243	0.14496858550813
H	2.45742952680661	4.70322844179201	1.11798697391047
H	0.74858660460075	4.15485377946106	-1.35900100741314
H	0.46802697657077	4.74346958160409	2.36248171813631
H	-1.02115329817525	3.81595501092807	2.51956915257328
H	0.51204125621104	2.97861567379476	2.31755219402772
H	-0.85475623997926	4.79263519702608	0.26237848994936
H	1.84441935339282	2.19783432679584	0.70424702224456
H	2.35800163659769	2.29987372990338	-0.98683123292655
H	-1.74096269203636	2.87398121639332	-0.78314120509595
H	-1.59040989608286	2.19325985727484	0.83078222644331

TS1C-2

Mo	0.00760897544529	1.47152808468032	-0.05175211265226
F	1.76661175253893	-2.56294702933319	0.37768283919290
F	2.38985410748790	-2.13024714339022	-1.64467365175150
F	3.87249318065580	-2.39721215120014	-0.08004967196483
F	4.51276885892509	-0.10804333300012	1.35929512508893
F	2.80947433859317	1.17077951241033	1.76721732038229
F	2.71970687112764	-0.92482913006359	2.26503646719476
F	-4.62735380860145	-1.42559366118015	-0.03666666486735
F	-3.97317150863871	0.51695635137457	0.66215812292533
F	-3.84649075319249	0.03490622571863	-1.43816054257587
F	-1.47524953097575	-1.07180099889185	-2.26199046670376
F	-0.73435714301188	-2.60478286710485	-0.93792625585628
F	-2.78289448379996	-2.68144761281939	-1.64106439918606
F	-2.89469558996378	-1.33446978458603	2.29716630810014
F	-2.67499828395169	-3.11669356530186	1.08836416595115
F	-0.92407442639078	-2.00979231404116	1.72640580794210
F	3.71652304988649	1.65456987279988	-0.73801053257771
F	4.50693778976384	-0.20431657467537	-1.50556377296319
F	2.62817938320814	0.56945119046821	-2.25870021730668
O	1.11477084719607	2.69674391949482	-0.35906386046056
O	1.25133676661149	-0.02226191970544	-0.07277087316898
O	-1.41017384774448	0.06355051191361	0.15027048273017
C	-2.07997778895416	4.37840599577726	-1.13366050884539
C	-1.01556581863617	1.32655450056459	2.90175503402350
C	-0.48245120973922	2.27880518370061	1.85515218772639
C	-1.41403568282462	3.11768790257604	1.09342398553347
C	-1.94546345212349	2.90979081865716	-0.79930112702831
C	-1.80416241279859	-1.86380097089924	-1.23105887649735
C	-3.70479525811462	-0.47581421675860	-0.20176860718840
C	-2.18097823158568	-1.89085274493722	1.30251715262853
C	-2.23779017298535	-0.99333634306470	0.00902601844935
C	3.17720208441762	-0.05274236629568	1.35831479413804
C	2.65905936787259	-1.89777759476136	-0.35262430465370
C	3.38023678332452	0.43131568390897	-1.16094707810442
C	2.57463433828634	-0.35531917520400	-0.05800633274396
C	-1.14359408575055	2.12194039984397	-1.72990332438445
H	-1.97788533940317	0.91127037289532	2.61747305062348
H	-0.32845121839275	0.50025490084835	3.08775347691254
H	-1.14129279370759	1.86640533668475	3.84583329580212
H	-1.09938199254962	4.84367662207241	-1.24497159860158
H	-2.60659643004241	4.47249855646105	-2.08645059976579
H	-2.64601710578021	4.92513962372657	-0.37854836217453
H	-2.88806616890429	2.43187498725873	-0.57211406547611
H	0.41703306863234	2.79544560489712	2.17429541099338
H	-1.08592931386994	4.14187852775573	0.99451105620346
H	-2.44628283104119	3.02699954626288	1.40612843507160
H	-0.51370273830237	2.67659278531711	-2.41581692901237
H	-1.62371214281245	1.26278847914503	-2.17686580110222

TS1C-3

Mo	-0.02177918502815	1.54101198785825	0.04746937850072
F	-1.91508534344391	-2.33489143555625	1.09652474089982
F	-3.13736026544147	-0.95704592816446	2.21748146920875
F	-4.01733400136326	-2.15269571437687	0.63165004601309
F	-3.91383674290278	-1.00002840024292	-1.91766672372781
F	-2.02716765765410	-0.00942790965988	-2.31897594334453
F	-2.04287170616230	-2.04423770383573	-1.59755668425469
F	4.50144526911188	-1.62241572105405	0.31004076398877
F	4.03280121615765	0.48380354297736	0.46523722316895
F	3.44876278491881	-0.86352794212007	2.04549363539969
F	0.91258652491780	-1.87502859311984	1.81578990702998
F	0.51239069149301	-2.68021439359273	-0.15211175727851
F	2.33368942490072	-3.21957267592595	0.88539181512255
F	3.28790937446207	-0.35476567620260	-2.02965238596940
F	2.90743309972408	-2.47194300463183	-1.75326206789421
F	1.27531002961498	-1.12110790302977	-2.19955141690512
F	-3.61849711081611	1.66476254317775	-0.89001404907495
F	-4.86625904398396	0.38242753203742	0.32273527192240
F	-3.31178120952699	1.56718142408555	1.25127259420138
O	-1.14056630859884	2.76949389662008	-0.18954933551056
O	-1.29774121547709	0.07892491450790	0.22805563144315
O	1.41531811939663	0.13179413818577	0.15799250906940
C	3.13399929761172	3.21421199976874	-1.19885911345688
C	0.91860511247836	2.15666130670020	-1.78650472280363
C	1.64044214188362	3.13864107154401	-1.00176390781080
C	0.98697217598093	1.42463026924328	2.97482329182757
C	1.70143232929327	2.96838708472440	1.02715167367547
C	1.47143936536057	-2.21708863569313	0.65345287392688
C	3.57161723725770	-0.75254867605073	0.71413789009615
C	2.41932210644916	-1.25118628833932	-1.52066405300059
C	2.18251647445096	-0.96973274189759	0.00711914587994
C	-2.65345174486434	-0.86089552523575	-1.49175338097321
C	-2.92240952978226	-1.47264504382593	0.99675396534256
C	-3.61913701118124	0.84663282219143	0.16860098741596
C	-2.57873502390284	-0.32186712283233	-0.01900947924680
C	0.65579587091207	2.41047165030961	1.87587416511338
H	3.32276996640964	3.59856627492315	-2.20495924481766
H	3.61623534138277	3.88444029193545	-0.48628053974590
H	3.59513867434976	2.23153260519012	-1.12721958915522
H	1.30670270149836	1.97397336018889	3.86644899441540
H	0.11412543343410	0.83170615936132	3.25386215091521
H	1.78338834957338	0.74402790124890	2.69198857902950
H	2.64772564740748	2.44945911656230	1.08717057926322
H	1.79661963405013	4.04510454223822	1.04951560848313
H	0.13327295053293	2.52053059240226	-2.43761201347348
H	1.50617875810171	1.35632913111192	-2.21465464257109
H	1.15185923001296	4.10512551710398	-0.99134541706268
H	-0.10754223299962	3.12357573918941	2.17028157672471

TS1C-5

Mo	0.10512008771249	1.58693132703428	-0.00711411144736
F	2.60070149185062	0.57180164678992	-2.23864220609390
F	4.40490273732374	-0.42854013287532	-1.57738311309731
F	3.82694969929290	1.45223470852679	-0.69053339961669
F	-0.92680053980992	-1.66519853487165	1.77539938784128
F	-2.69375726404273	-2.80829581835749	1.26147967320725
F	-2.88422056611289	-0.96873207840421	2.37524133515778
F	-3.01257951888662	-2.53190099358822	-1.43444878848773
F	-0.92701440468356	-2.50842479515779	-0.84339912255252
F	-1.66161807232345	-1.05561251674125	-2.25160385450486
F	-4.00571268979394	0.17307755465126	-1.37469962204354
F	-3.94625973436195	0.86565982985688	0.66945224187597
F	-4.69466486838258	-1.11776894796701	0.22221963196952
F	2.60498366805559	-1.14455389500688	2.20800541740561
F	2.86844015286914	0.95427817370832	1.79869603136248
F	4.45252408829763	-0.44470438160577	1.31478716827444
F	3.64481622141458	-2.59446774940210	-0.23179334000652
F	2.14363793717947	-2.15703224295846	-1.74008055497487
F	1.54837308393477	-2.63624900549191	0.28130685002871
O	-1.44622771044656	0.29956836680022	0.04967197707318
O	1.19522970960806	-0.04013812868827	-0.08920824804512
O	1.38848187134835	2.66142328175725	-0.11812655873530
C	-0.79783619883826	2.62601543783947	-1.69411862222403
C	2.49090591628990	-0.46873285535861	-0.09002628476804
C	3.35630548929807	0.29536691605468	-1.16729108010524
C	2.46268892653654	-2.00013098453511	-0.44717863495989
C	3.12536525434122	-0.27553261423270	1.33238334151532
C	-2.30087542956220	-0.75090185207633	0.04318654304859
C	-2.19773081904847	-1.57154758957917	1.38842758520079
C	-3.76951960956394	-0.21422680933152	-0.10956767110508
C	-1.97215897340797	-1.73633558832961	-1.14543855977935
C	-1.64853166592516	3.20994499323756	-0.67579982275810
C	-1.38484947542376	1.81009986458061	-2.81786702261484
C	-1.15414424113639	3.29380859379277	1.26693073636073
C	-0.32378141231377	2.33293150176559	1.97132367391527
C	-0.95174775373815	1.39654763571908	2.97252721468526
H	-0.01261909152952	3.28940284704324	-2.04007539354378
H	-2.20513764580564	3.26880375207467	1.52341241741767

H	-0.74474337230672	4.29187859510469	1.20832434257644
H	0.63321850558641	2.72369553750653	2.29858886520254
H	-1.68847896797784	4.28919463118468	-0.64924740653219
H	-2.62191372783807	2.74650321228458	-0.58161508740069
H	-2.25331186872811	1.24129325559247	-2.50757443435030
H	-0.65255878398689	1.12262737287588	-3.24371130733545
H	-1.69358155502619	2.48947765024172	-3.61963706806937
H	-1.00538853325890	1.90165219516038	3.94266120857966
H	-0.36508418618129	0.48882194085919	3.11110507930183
H	-1.96013616049794	1.11622669251661	2.68573059315179

TS2C-1

Mo	0.11691796589199	1.34546989703805	-0.44887056604355
F	-2.27941018472076	-2.60584062560129	0.81096669887961
F	-2.18244741323533	-1.06474139069650	2.32096857255605
F	-4.08831485810457	-1.58939702186643	1.43933275747339
F	-3.37784147728639	1.26951448507958	1.62700711405483
F	-3.03127115135627	1.95828259027236	-0.38903470218995
F	-4.72193607954875	0.67542603524868	0.03342418985523
F	-3.03128419340387	0.02682212541084	-2.25430580596752
F	-1.88495031700907	-1.73624949730637	-1.76570952348604
F	-4.00341409183787	-1.66833378975187	-1.31890585350175
F	0.95747183697155	-2.21010967294498	1.77696546577573
F	2.39429612490915	-3.39758419439248	0.67471256536515
F	0.51999615067721	-2.82108849570533	-0.24866419055207
F	1.20369148216551	-1.03072872813753	-2.15687151561978
F	2.88896699377389	-2.36106100677477	-1.86538439740441
F	3.18885329452369	-0.21486937530367	-1.94056569118745
F	3.93847905930056	0.41980945962337	0.54947230474800
F	3.40744137022594	-1.01745530255202	2.06991208178428
F	4.48090707929709	-1.66061145376906	0.29882069936444
O	-1.20260408423212	-0.03189099839228	0.12931553224026
O	-0.46517577303859	1.49190607838819	-2.01004141342436
O	1.31716718889119	-0.04091279756635	0.35578648196757
C	2.97214065496687	3.60697853056910	-0.30682896562732
C	1.92675629810731	2.42908140618994	2.38021377088533
C	-0.40719483602029	2.48329501596251	1.29127608710523
C	-2.52151423925823	-0.34120823762855	0.06724907784801
C	1.52094358233553	3.28885429013064	-0.05638927414449
C	0.99679507666241	3.13209149510207	1.39728397377800
C	0.50163157413506	3.67390361392331	-0.92315776763873
C	-3.44082852819795	0.90224606918620	0.33666909859315
C	-2.78160394616778	-1.42331703917812	1.18140569497698
C	-2.87232671219080	-0.94712182825822	-1.34528333345656
C	2.13810434008961	-1.07385348496468	0.03895023603103
C	1.49016827572741	-2.40906241150284	0.57212964136326
C	2.36361821211173	-1.18773342234877	-1.50944153977077
C	3.52221566076124	-0.84424003441858	0.74709668715031
H	3.18134649296419	3.66414915032175	-1.37421462010292
H	3.64004308313894	2.88048582300334	0.14831140720658
H	3.17870673138016	4.58694850486111	0.13401467334172
H	-0.44646108838872	4.03870518506140	-0.56403861097594
H	0.71818784813011	3.85476688016726	-1.96735531531627
H	2.91300948344374	2.89315299812558	2.40117696054756
H	1.50381431424457	2.50334545702400	3.38238864133471
H	2.04533153301054	1.37461028912234	2.14411156462651
H	0.88509394856089	4.16995840512450	1.73198720638362
H	-1.22695566775561	3.18976194442867	1.23403942422193
H	-0.60649576270103	1.74863372452571	2.06927344039265
H	1.76992474805589	1.79511135517016	-0.70175896344127

TS2C-2

Mo	0.07731420475730	1.26748151222941	-0.27955838833969
F	-2.22996892273125	-2.82721901430214	0.35381290013137
F	-2.16596988169720	-1.58770777930558	2.12128245407886
F	-4.06359335080120	-1.99952442312306	1.16699713082679
F	-3.37362736783994	0.81644427653372	1.88234736961169
F	-3.08400699882223	1.85942719030698	0.01588368760295
F	-4.75484040204805	0.50052329671044	0.24119272558347
F	-3.14129099948038	0.29716498723274	-2.16313539479668
F	-1.92059896575550	-1.48029266590388	-2.03806149631186
F	-4.02626679401775	-1.57669947806499	-1.53611914528861
F	1.28812073561119	-2.48550307381309	1.64098430673992
F	2.86738888619121	-3.33761601173560	0.42957361908784
F	0.91660108815355	-2.94328687570676	-0.43692677652350
F	1.38255888799282	-0.90071359589521	-2.18647110891182
F	3.21864695771722	-2.03096681648455	-1.96421302166375
F	3.24687674213164	0.13185123772109	-1.84282004891510
F	3.77584460955818	0.69444941672174	0.73784847551428
F	3.48801447032764	-0.96135662699275	2.09091313469509
F	4.67554585289570	-1.22734918281367	0.29669198992252
O	-1.23166617429879	-0.16360139387268	0.13399357481083
O	-0.53830683601646	1.64813991163966	-1.78900216991463
O	1.28026987738522	-0.17854954586748	0.39365575218286
C	-0.34932438119009	2.25367977119118	1.56383895478653
C	-2.54624199413976	-0.47664313267569	0.03824393654262
C	1.56240426870495	3.04707516119671	0.21391091871623
C	1.12484925772264	2.70498114534372	1.65486925906766
C	0.61503405600612	3.73505000597047	-0.54003818411062
C	-3.46754436624920	0.68589823617857	0.54957262137207
C	-2.76498465091883	-1.74922552994294	0.93813428255645
C	-2.92095035864213	-0.82078166689179	-1.45448878586659
C	2.27179811299147	-1.02553256432603	0.02538662555516
C	1.83025361073071	-2.48385316022303	0.42444711344235
C	2.54099005154551	-0.97134998030411	-1.52125455586071
C	3.58401835477007	-0.63882361215507	0.79645238372424
H	-0.62577710768309	1.49456159766330	2.29194093876350
H	-1.06364277284846	3.07051103969311	1.58624432822716
H	1.73388613137817	1.87292238357344	2.00613348736876
C	1.35911509407243	3.90147667854946	2.58742828825349
C	0.88457104548736	4.25505184231583	-1.90690127216739
H	-0.30538693226115	4.04539842617198	-0.06959832439158
H	1.72886126929577	1.75537332110440	-0.57698444859306
H	2.62514014996189	3.17510107502120	0.02094190316351
H	1.06728543085213	3.63734155292518	3.60433351327210
H	0.76536039909702	4.75997767459008	2.27160212946404
H	2.41259863832906	4.18900420405938	2.59666752240254
H	1.69900985520411	3.71670633383984	-2.39256269667129
H	1.16763992006120	5.31203690373468	-1.84164317238165
H	-0.00200870149084	4.18996694818176	-2.53654633675837

TS2C-3

Mo	-0.05850503492202	1.33178591512398	0.21370225303125
F	-4.71809676760074	-1.05804584995810	-0.04143560129435
F	-3.69138543345509	-0.74198393653112	-1.92377717917688
F	-3.80908709821084	0.85795412052558	-0.47937611955190
F	-3.01556528799294	0.20192135811617	2.00319666939333
F	-3.12710850911590	-1.96325698983388	2.03864155939070
F	-1.20663426511476	-0.96618202283538	2.14193824940745
F	-0.98012386282050	-2.91671279662816	0.27835737384949
F	-3.01232440799720	-3.20233025661033	-0.42550282964547
F	-1.51296744313490	-2.35579491053523	-1.73812117718700
F	3.88588810953904	-1.82153093421142	1.44624414841140
F	1.75318846293825	-1.78843859437199	1.82308071773455
F	2.94547291981649	-0.03789824404682	2.24280263593913
F	4.75532925909604	0.36101049705296	-0.04423712795998
F	3.13191127747694	1.76162968221618	0.24926761737556
F	3.48224719814416	0.91971806751476	-1.70712487146415
F	4.00950070298489	-1.96376544656672	-1.31344175100177
F	2.16330778153938	-1.37346717709889	-2.27758797841232
F	2.12211327893316	-2.81715569313340	-0.67226476716838
O	-1.29605630955244	-0.11599809280275	-0.41476011031501
O	0.47643404013701	1.65147772098579	1.76551188951554
O	1.20372872240695	-0.14987122782465	-0.17036454346002
C	-0.81879065235261	4.29098425405562	1.84515274495010
C	-3.65956101347919	-0.47418801993752	-0.61219852273668
C	-2.41736289388430	-0.93020365812255	1.58017959026764
C	-1.94625093881953	-2.38724201029393	-0.47771049444164
C	-2.29229494764068	-0.93114741593787	0.01488368679765
C	2.78192597726576	-1.06115245494011	1.39027977642230
C	2.71130209892797	-1.69649282453165	-1.10171277343595
C	3.49588955358163	0.63810697814531	-0.39388858250518
C	-0.45286323365253	3.80409460153671	0.48718078026039
C	-0.81220159199755	2.95211741137481	-1.80678921593228
C	-1.36733456633060	3.23256155723911	-0.38984940175491
C	2.49968963572215	-0.53007499828314	-0.06568375221331
C	0.57469137054832	2.29950254809413	-1.58963255323754
C	-1.73935616116963	2.15119123885404	-2.71646863782001
H	-1.68467377167228	3.76162237048598	2.24415768788092
H	0.00982389419297	4.18048958878746	2.54294887039620
H	-1.06933791487318	5.35658759109828	1.79016333796597
H	-2.43412781688051	3.40467102486608	-0.27277285355454
H	-1.70144247362183	1.88152466359608	0.35902638826938
H	0.52658168032670	4.06894573924270	0.11586422637407
H	0.80758335846847	1.51473229133900	-2.30687256890305
H	1.39869548140631	3.00257235752775	-1.53407625490247
H	-0.68666223052616	3.94484881862460	-2.25073442322868
H	-1.80521659820928	1.10761594836443	-2.41738675119496
H	-1.35640921907907	2.18350453992639	-3.73685223548683
H	-2.74656435934629	2.57076267034161	-2.71995712564774

TS2C-4

Mo	0.08741225974617	1.36266437538938	-0.10563483713041
F	-1.98925530130155	-2.88394950074058	0.13905185609251
F	-2.19606680232552	-1.79143694018928	1.98983446142084
F	-3.95170833817434	-2.24374642241656	0.80810141457599
F	-3.66285436009414	0.47778696019299	1.77536066539172
F	-3.14335030803585	1.74777349674659	0.11018665438690
F	-4.72564711550075	0.28151032522320	-0.09986592573814
F	-2.89973894143629	0.34263234386202	-2.23256874696300

F	-1.50573740335499	-1.30338378469955	-2.09556368533585
F	-3.62748420113796	-1.65538466054366	-1.83633324785393
F	1.31780663217596	-2.46686830899047	1.48421532383412
F	2.93347394651364	-3.21371663452069	0.25113998283412
F	1.01545557678987	-2.72649205498865	-0.63902190955265
F	1.53255889209957	-0.53864534478681	-2.16638395192707
F	3.36802789255505	-1.67879917802878	-2.00141318715028
F	3.38028869756682	0.46318048345322	-1.67467681369946
F	3.85893606093188	0.76007267015028	0.95479924961067
F	3.51545271991123	-1.00882636525845	2.14298780322969
F	4.75477802198031	-1.12356333619378	0.36752907025832
O	-1.18422571797074	-0.12971118232106	0.24543321276252
O	-0.59572523720344	1.80131315102806	-1.57020327260820
O	1.37685987647858	-0.04314533234216	0.47324791110481
C	-0.27563430692377	2.24656819632837	1.82041606575638
C	-2.44474823704630	-0.53985356135327	-0.03455655651925
C	1.52792361961847	3.18665435458132	0.35591845125925
C	1.19154891550864	2.75294353035317	1.78963589692366
C	0.50496400409482	3.87403237838959	-0.29641462505195
C	-3.52228345089212	0.50133758753816	0.43956702518866
C	-2.65776112644694	-1.89310991079703	0.74032696791371
C	-2.62759646602407	-0.79786793648078	-1.58180793581903
C	2.36234844473096	-0.87004506758046	0.04701751226695
C	1.90084555880699	-2.35585869013445	0.29141729298572
C	2.67329556298207	-0.66783890455952	-1.47867896242124
C	3.65464951612312	-0.56948905156260	0.88717780404155
C	0.65856038637688	4.45923308588543	-1.65496096422528
C	-0.55963061986814	1.25079721717184	2.92817924459390
H	-0.38520178523303	4.13583547044865	0.25418423701724
H	1.69445394058504	1.95563223050382	-0.48075771616204
H	2.57037120126109	3.37563730928367	0.10763618483226
H	0.90652530488625	5.52333001111973	-1.56709027394301
H	-0.26758280116339	4.38251558717113	-2.22351518542225
H	1.45462775172098	3.97157842044705	-2.21843933104874
H	1.33197576532351	3.61199888517412	2.45249685672295
H	1.87291927886860	1.97261218327119	2.12140909153852
H	-0.98478927834397	3.06973282000622	1.83727765182705
H	0.06919218997386	0.36398260619259	2.85018557758894
H	-1.59640990561835	0.92640399337169	2.91314276689817
H	-0.36582031351471	1.71597249620508	3.90201089571465

TS2C-5

Mo	-0.04736932730635	1.33569022102607	0.15983427876139
F	-4.79323117556889	-0.91171758730262	-0.42322805769332
F	-3.53471649618982	-0.79168160778248	-2.18460173109256
F	-3.78300142271344	0.93963404974946	-0.92046143069832
F	-3.35236975245361	0.49955302232403	1.69236294492555
F	-3.45831320750729	-1.65078903992358	1.93006826512984
F	-1.56536420870089	-0.61997179344872	2.15380320822272
F	-1.16735652973397	-2.77791054920544	0.53731258562574
F	-3.11007724856490	-3.11304544053104	-0.37027539628371
F	-1.44928559231414	-2.42314864849448	-1.57470947251255
F	3.54154530044576	-1.93540501473650	1.77534490901568
F	1.43124695829752	-1.51783224760326	2.03532999725319
F	2.88322795565953	0.06253294191447	2.28363552021652
F	4.75465617125496	0.00202482238939	0.10764217144835
F	3.26322713680789	1.57321837577071	0.07446482970188
F	3.65415133798379	0.42589814766855	-1.71119929420820
F	3.83931618549421	-2.40352099652423	-0.90566168637404
F	2.12230510814915	-1.77745742557784	-2.06429892979490
F	1.83820410293933	-2.96808699978482	-0.28544385845628
O	-1.34489265905078	-0.04978212267444	-0.46574422633391
O	0.61744267494613	1.71227908487154	1.64918589845999
O	1.19497196290963	-0.17005511440479	-0.22670916050390
C	1.46164231106539	3.08016950050716	-2.26517659273665
C	-0.60246467725743	4.38569871530516	1.73974412998481
C	-3.65740641362841	-0.40247080615545	-0.91086229431475
C	-2.70428633475397	-0.65906883652379	1.45391416137406
C	-2.02662549347458	-2.31939130441112	-0.37881967574802
C	-2.39288081059458	-0.81680280176882	-0.07790546834337
C	2.57757619174029	-1.02683205484868	1.56324671220856
C	2.56740985301911	-1.98271003936969	-0.82158267990403
C	3.55432437739827	0.34024355818309	-0.37337597076647
C	-0.42066474282615	3.82065660217296	0.37481102442579
C	-1.08540343441901	2.76922879627783	-1.76381641139075
C	-1.44103122288490	3.18407991098340	-0.32346114014879
C	2.42494307715941	-0.66916360846865	0.03432174351538
C	0.35102928273886	2.18034190992411	-1.77400865354497
H	0.37829172432452	1.23636558682895	-2.32311623162993
H	2.42157311655350	2.57136912554962	-2.21309114302712
H	1.28663321979604	3.35029987032341	-3.31230914573898
H	1.54635811308917	4.01509055189894	-1.70931504894887
H	-1.81427238198133	2.05471959628698	-2.13612944652225
H	-1.14624336916997	3.66530113175039	-2.38803732333586
H	-1.41426196439204	3.89376808273244	2.27656875145596
H	0.30908619359829	4.29489122864349	2.32883108845553

H	-0.84393737627769	5.45187752158054	1.66171383086363
H	-2.48471492427181	3.37885698637895	-0.08932769502221
H	-1.65364725721915	1.91584553674946	0.51105780458880
H	0.49365566788437	4.06520916174926	-0.14052569055860

TS2C-6

Mo	-0.01599692786286	1.24403003604513	0.43673088456634
F	-4.75981455250269	-1.03943345107541	-0.48845094455881
F	-3.46086708956136	-0.74038773811043	-2.19849158483737
F	-3.83766753632055	0.89069275390851	-0.83616725203035
F	-3.43922485043092	0.31092128336072	1.76069326038002
F	-3.46586259902979	-1.85464429345709	1.84891550744410
F	-1.62345553227348	-0.76949376929646	2.20189452679344
F	-1.05749514923827	-2.76671685944975	0.46028034747535
F	-2.94460930857185	-3.14402875301220	-0.54203690740828
F	-1.28713638781793	-2.27201895540576	-1.62998669415183
F	3.85528056839560	-2.01182623738736	1.38527196705281
F	1.77374144554160	-1.80843843249695	1.95337414576591
F	3.11675207275851	-0.12930252012538	2.15856565811224
F	4.69155683409060	0.09845812783621	-0.28861308537640
F	3.14900890659535	1.56836815634594	0.09118361649526
F	3.28779333324433	0.63804725801886	-1.85091996953901
F	3.77911904179065	-2.25537216059762	-1.35360181152777
F	1.87988479231666	-1.65554049959330	-2.19909338123608
F	1.92553376059309	-3.01058467143872	-0.51790742734317
O	-1.38709749058283	0.03366936711799	-0.35655971932458
O	0.65564097494165	1.46145114479468	1.95766484171219
O	1.12890678569081	-0.28676823119247	-0.10261058337568
C	0.94934908903251	4.46862163137340	0.70733552375906
C	-3.64081421391967	-0.43886770756117	-0.90798114776262
C	-2.74196430159501	-0.80332283244609	1.46454831663723
C	-1.91045131831306	-2.29105564278486	-0.45379270326804
C	-2.37923619931702	-0.83926450538176	-0.06197893973612
C	2.80253933053745	-1.18013032584860	1.38532798098900
C	2.51022800982922	-1.93402533964524	-1.05540963566119
C	3.41484508092802	0.40647738605953	-0.53532610010008
C	-0.31284713597718	3.72859805191967	0.99510795721606
C	-0.96714466758460	2.99745351019831	-1.33171563330647
C	-1.31176383439586	3.22140206489534	0.15179074232870
C	2.41606319926289	-0.71039573085990	-0.06888412070710
C	0.41908863375092	2.30807442595683	-1.35754979905068
C	-1.07132696517127	4.29391602061428	-2.14751226786401
H	1.26493814332875	2.98035406962790	-1.41350272243467
H	0.50766425768954	1.54436982971731	-2.12862894603845
H	1.80419562065395	3.98256449247334	1.18340554594475
H	1.16374045757819	4.61139332362613	-0.34360475907540
H	0.85682477329003	5.45626178169175	1.17240012322798
H	-2.35770084708047	3.40226770796241	0.39620736146185
H	-1.59375945403694	1.89021637740514	0.81726023231923
H	-0.58953282637353	3.76812121810983	2.04448923680950
H	-1.70482796653277	2.29989366525628	-1.72803758389673
H	-0.35559343508086	5.04399603931724	-1.81352743330688
H	-2.07435206269236	4.71869208006362	-2.06925623279995
H	-0.87415245957717	4.07930685347013	-3.19830038777329

TS2C-7

Mo	-0.01531958451489	1.31010546955010	0.36186817759213
F	-4.80328475144868	-0.83750740662505	0.07976913256677
F	-3.85341101418240	-0.35168069748436	-1.80711788944098
F	-3.83820564059024	1.08222042907695	-0.19397143502474
F	-2.96357448357762	0.13025371600569	2.15787492642788
F	-3.14833117266659	-2.02210032295109	1.96780613065483
F	-1.19190637570430	-1.09805359012955	2.07992270665066
F	-1.12779782522127	-2.84488928275748	0.00661720518006
F	-3.19931944113616	-2.98412897076951	-0.62209585861247
F	-1.73065920935662	-2.04606127508161	-1.90735719548498
F	3.79721755491684	-2.04911521323723	1.34228512258326
F	1.68684214044620	-1.90890038868254	1.81047527395301
F	2.98790774342080	-0.22862653188511	2.19465118625905
F	4.71243078222121	0.11619856160717	-0.16005642375055
F	3.17062234358763	1.58541589757112	0.22298300455322
F	3.39678687519129	0.75970545787194	-1.75815113133555
F	3.81352917141025	-2.14975594147843	-1.42040113162231
F	1.95608138137786	-1.47261744394830	-2.30172989154107
F	1.92005839638345	-2.93207035806054	-0.71036764597629
O	-1.37542877237498	0.03154572439585	-0.35894182013887
O	0.59317595002167	1.51845863284323	1.90912311898063
O	1.13835008301505	-0.22526543287539	-0.15158311311788
C	-1.81096870559362	2.61015325887927	-2.42735813066992
C	1.01991587110588	4.50358514283457	0.61466060573071
C	-3.75017976161240	-0.23140705204043	-0.47741204701132
C	-2.42648321024978	-0.96629656948916	1.58751625098373
C	-2.10673707021389	-2.20341614887266	-0.63772484857528
C	-2.37494288432918	-0.80117493567151	0.02701887867528
C	2.73323939363991	-1.23189731909992	1.34022241825972
C	2.53692929061236	-1.83155765853140	-1.15307593657912

C	3.45379166398870	0.45513829215905	-0.45206352119978
C	-0.26256772376998	3.78918223115565	0.88229095067952
C	-0.75493788540587	3.18011301884443	-1.48601089053467
C	-1.22549535749040	3.33288274084247	-0.02462751076847
C	2.41727065084008	-0.66965186814078	-0.09727281520899
C	0.54437804056112	2.33758133989421	-1.42295355757184
H	-2.74472227509246	3.17136935276368	-2.36202859419935
H	-1.45225702527673	2.66983786732623	-3.45527059345465
H	-2.02243360151059	1.56585432773317	-2.20698285465274
H	1.46383728371567	2.90837016006219	-1.44065883854468
H	0.58848038496093	1.55315665242398	-2.17849340141553
H	1.87849902900901	3.96248688102684	1.01854523186313
H	1.19725562271026	4.70838691697951	-0.43649196931648
H	0.97834128732413	5.45817532308253	1.14870139851711
H	-0.51668703193376	4.19669047539595	-1.81923101003346
H	-2.28104548420094	3.52045540380378	0.15965438967561
H	-1.60174485662291	1.96753605626347	0.62456566721993
H	-0.57949979638399	3.83331507741897	1.91887827877574

IM3C

Mo	-0.04381938198563	0.83897813531136	-1.05456209762488
F	1.23138505274035	-2.04924255366916	1.96047430130645
F	3.30736081469388	-1.42521938379111	1.95884472163433
F	1.72409996719311	0.04614954837370	1.82221507461451
F	2.99313328519734	-3.51638889358770	0.14905359559907
F	0.90372864434925	-3.32753598749776	-0.38685037185650
F	2.42871052937156	-2.90855292077175	-1.85598000146214
F	3.62166515777312	0.58482971957488	-0.07104167977994
F	4.48652810282495	-1.33469494504515	-0.57615265432118
F	3.22314162181685	-0.29900826718198	-1.99969716653990
F	-4.96300925880601	0.51204979365850	0.90072156778961
F	-3.99962442663202	1.70433484359725	-0.63103052563212
F	-3.22831885484619	1.71622806648797	1.38584412813354
F	-4.40064048241007	-2.07585330137152	0.11672755829754
F	-2.78099127780745	-2.18544639906590	-1.31886267074224
F	-4.40447142586383	-0.79802545579167	-1.63746874254956
F	-1.66971705423468	-2.05605134517592	1.13659322220817
F	-3.09484207746811	-0.97996196979653	2.36316552741318
F	-1.21024156159044	-0.05585590510817	1.80773595215209
O	0.34466562823769	1.37589580645911	-2.57847651157380
O	-1.84044689795814	0.20028940834716	-0.75435715490148
O	0.98257761854596	-0.74261140471186	-0.67839382421459
C	2.10094973387203	-1.17907637998767	1.43621692306634
C	2.11462242848892	-2.79811641179386	-0.55982215149750
C	3.39467621206439	-0.57448872502862	-0.70634829335939
C	2.11552596297832	-1.28450298308256	-0.12954307499147
C	2.80757849280917	3.56831853927593	0.97058360141175
C	-0.50380378208129	2.71382191787201	-0.14056120711426
C	-2.82901234709172	-0.25135396306099	0.07625050393875
C	-2.20415162074706	-0.85848330522443	1.37851719772489
C	-3.62574189290818	-1.35746884425866	-0.70458608053691
C	-3.78515363572776	0.94014320934026	0.44096661899978
C	0.47809552486049	3.87894647902321	0.01288962014896
C	1.48409877532516	3.56171590846281	1.08548159800624
C	3.75695057986299	3.21936957092623	2.07164173143463
C	1.07794228850253	4.32389723298236	-1.31627478587975
H	1.19567789544348	1.48455925994993	-0.15922285609456
H	-0.89787887084237	2.42073450928911	0.83589049513295
H	-1.35309616233796	2.98878001778188	-0.78367040755810
H	3.25812732395288	3.83379316211006	0.01922678667140
H	1.05145601423213	3.28360569889960	2.04517893610421
H	3.22727999048943	2.95146650833624	2.98686012097244
H	4.42636748972620	4.05561905125912	2.29417346701095
H	4.39166962132517	2.37621753146481	1.78540776998333
H	-0.13158539690850	4.71303884145046	0.39154755768528
H	1.72518333994979	3.55674038052180	-1.74148599216540
H	1.66513269324878	5.23422207318351	-1.18888180474007
H	0.28934561837144	4.52861413106370	-2.04232852230467

TS4C-2

Mo	-0.08032310798925	1.24231255222619	0.51691393373630
F	3.44102139836161	-0.35172960664981	1.95945185727048
F	1.59008659164274	-1.45943996358617	2.05218087434101
F	3.40392648779641	-2.44555220101060	1.39600896093522
F	0.94140052270928	-2.78579506537764	-0.19861940053220
F	1.22022805967753	-1.69758942735205	-2.04421938456303
F	2.81361448826400	-2.94972733009376	-1.27946384308437
F	3.47217565304944	-0.21051114421831	-2.15073153373887
F	3.95052330017775	0.91896892728013	-0.37702013878103
F	4.73763329465659	-1.08309537752790	-0.62057674195967
F	-1.72941023869994	-2.23523575248554	-1.62180422815322
F	-3.69896792648159	-2.61885631052518	-0.81602208006614
F	-1.93765327479907	-2.93529139405743	0.41165251856060
F	-2.09569103375641	-0.91919935005069	2.32013832504825
F	-4.06348704232850	-1.52484557489895	1.64082006954817
F	-3.56366474915464	0.57275268689127	1.78162381336460
F	-3.27560062224428	1.49918043261948	-0.72363206606508
F	-3.17558856223931	-0.03881854889344	-2.23242293096149
F	-4.75456060337342	-0.08439187155197	-0.74528812455754
O	1.43279706694765	0.10188247100373	-0.12509453186791
O	-1.17512960479071	2.08893202303484	1.46514664940468
O	-1.24837401916819	-0.18703629157639	-0.07732173193984
C	-0.06575901707963	2.36473965894103	-1.32145677345090
C	2.37317631949380	-0.86684865508084	-0.12558854782324
C	1.96842653953715	2.64084237029572	1.67730991550934
C	1.83024684174580	-2.10614715579305	-0.93124505234350
C	2.71363230608799	-1.30387854851061	1.34369295036668
C	3.66467540776183	-0.31473771182531	-0.83018184283435
C	-2.52803307505625	-0.65861590155221	-0.01323363882142
C	-2.47934263878874	-2.14663361332091	-0.52444582228068
C	-3.08189884597377	-0.63280080419111	1.46247095985616
C	-3.46155564341941	0.19003404356385	-0.94417437856751
C	1.35330870068608	3.51606874292755	0.83790403347389
C	1.74397480669159	3.84307770240361	-0.56500421637437
C	0.49904394197941	3.76795978136518	-1.45879918010842
C	-0.54527019960401	4.83608395140847	-1.13146193560461
H	2.83489932001497	2.07670327664240	1.36336712600684
H	0.90978949606660	1.17512411029606	1.87048509206173
H	1.76875049534545	2.67669041386325	2.74000687595731
H	0.57149621033061	4.13400300154256	1.26324663112511
H	2.50962153642345	3.15138436194312	-0.92086257125569
H	2.17113443411012	4.85176384645647	-0.59302505673232
H	-1.11408744254369	2.30020338993588	-1.61808024735152
H	0.50619053595326	1.63674765757640	-1.90288425491841
H	0.81447569407364	3.91863105153683	-2.49802424267021
H	-1.00369267534373	4.67374971145671	-0.15477762102891
H	-1.34775127038777	4.81546824266087	-1.87022284720077
H	-0.10452785636245	5.83510319225812	-1.14359562092906

TS4C-3

Mo	0.06613353090555	1.23178715036619	-0.67855412060383
F	-3.30681762510331	-0.78321134334385	-2.03835433789441
F	-1.37675922255044	-1.74751233343278	-1.96503482456100
F	-3.12990748636674	-2.78988909700863	-1.23484275949122
F	-0.69292117925999	-2.75321263051769	0.43040587854831
F	-1.09817178065672	-1.48675838968690	2.13348022515023
F	-2.57143373298648	-2.94056070907108	1.49566459352743
F	-3.45971928956286	-0.16452314178416	2.02481257331381
F	-3.99146222164590	0.68625297237944	0.11542991837240
F	-4.61357316200962	-1.32853428500622	0.60386353821680
F	1.89295758978611	-1.96702771856928	1.72589291538492
F	3.90219617775638	-2.26499361923151	0.98460877408849
F	2.19519586306520	-2.78624327309973	-0.25045494793372
F	2.27845215453122	-0.90932403849913	-2.28971191027458
F	4.26802627824184	-1.29956968071765	-1.52186210612436
F	3.60187862577558	0.73426751942205	-1.82453372991517
F	3.18103745880034	1.79786241181533	0.61206333900377
F	3.15749694885448	0.35696648668171	2.21603282597275
F	4.77452368174255	0.33764718953853	0.77025360455729
O	-1.40080815568688	0.05908979493186	0.01922612843739
O	1.11232136292942	2.13873795949040	-1.62594796077682
O	1.30722654953876	-0.06997788632367	0.02439644141028
C	-0.12834736343201	2.49659174576367	1.04986048622147
C	-2.26411625111823	-0.97356687082450	0.11395064037675
C	-2.07742026026243	2.40003258732067	-2.01461398823347
C	-1.64898744104136	-2.06701760065082	1.06579759583407
C	-2.52829728177872	-1.59903051958251	-1.30227479770658
C	-3.61348741037387	-0.45095848992991	0.72538656965076
C	2.61982292115023	-0.45157314913607	0.02777241932552
C	2.65998677369940	-1.90109272342199	0.63907392341022
C	3.21219117718126	-0.48276152598210	-1.43207961399688
C	3.46008172346564	0.52420629468959	0.92254376824368
C	-1.60143426058713	3.36802975314832	-1.19701416288900
C	-2.08218434246667	3.71152483819410	0.17255241457508
C	-0.86134253564826	3.82520636665882	1.08733252137775
C	-1.22347429462781	4.23560964332622	2.51476209238506
H	-2.89427937802689	1.75891525025352	-1.71572920610569
H	-0.82387918356325	0.94810704822217	-2.06450467019442
H	-1.79416128123851	2.37930273500320	-3.05804224410272
H	-0.84585892423948	4.03649631749892	-1.59952044243749
H	-2.77623574891737	2.95574035590635	0.54638640042309
H	-2.61936472505918	4.66571909973732	0.13679246176865
H	0.91871396041297	2.58491725184560	1.34780420359254
H	-0.61411129477213	1.76014625318363	1.69751569076307
H	-0.20159216087290	4.58924618266373	0.66268506834201
H	-1.90665396804477	3.51040371152528	2.96355612748816
H	-1.70918823349177	5.21373478207609	2.52888242197863
H	-0.33405258244543	4.29310732417739	3.14459026150095

TS3C

Mo	-0.01718525332373	1.43783251458226	-0.38699604627801
F	4.83791969993950	0.17509804018772	0.05462041137082

F	3.63297454769227	0.56096467627126	1.81778805237200
F	3.38688147004564	1.77685946805593	0.05524244426215
F	3.07402592740059	0.35721592237048	-2.23120973569570
F	3.71080124102435	-1.65752353953293	-1.76515483231011
F	1.61503308460740	-1.22936385533891	-2.11525134553956
F	1.95838491085768	-2.78546779911373	0.16421780445385
F	3.90407269183730	-2.20018949150515	0.91036568782551
F	2.11864706805932	-1.65342011953041	1.99952868611922
F	-4.34897431036208	-1.65055966060460	1.12395908309817
F	-3.87249620728202	0.45796171270593	1.24823136379831
F	-2.78784642278248	-0.95575465982866	2.45940264041935
F	-2.17889410612083	-3.27829147135003	0.69947370132790
F	-0.57683334481938	-2.01038992113381	1.40182553527850
F	-0.70001399411824	-2.52500528437790	-0.69737191985900
F	-3.45604928709182	-2.31566407208257	-1.47873593817105
F	-2.14030115292017	-0.78407795609707	-2.26318343331669
F	-4.03077899706244	-0.23492483863849	-1.37278406715902
O	1.25258566144716	0.03233672728467	0.17230361859481
O	1.07728826576345	2.27203108826060	-1.34137285756654
O	-1.47140576988239	0.16591659454058	0.13330859961645
C	-1.13425062757276	3.54706033219851	-0.00964709503157
C	-1.33259713582146	3.19952354562145	1.44344513623053
C	1.15585747326128	3.12627581772321	2.06140231388487
C	3.61767460972928	0.53041472288423	0.47403792112420
C	2.74061256159673	-0.75696925962017	-1.56728815795830
C	2.62958525644589	-1.80381113028189	0.77677514647882
C	2.50764963462108	-0.45881839821271	-0.03753195800439
C	-3.34520875430911	-0.77757915849484	1.25623468006300
C	-2.99062718206072	-1.08075418954441	-1.27318622723180
C	-1.41590339517119	-2.21907447206455	0.39103004941103
C	-3.31500437358771	2.75168522492161	-1.08402873115005
C	-1.88525089866892	3.19370268637852	-1.10842961611462
C	-2.27186725498239	-0.92785298638638	0.11882561361761
C	-0.07093111819387	2.36521791288411	1.61809098731449
H	-0.27828805441571	4.18326048181991	-0.19506198007782
H	-2.23085921082124	2.60696111625350	1.60521893012614
H	-1.37910464700415	4.08174525483917	2.08875838228404
H	-3.93474934980960	3.65124477947110	-1.15131103444777
H	-3.55718717746509	2.12052200099924	-1.93497357439645
H	-3.57892052958247	2.22758614613610	-0.17244630651144
H	0.98350934368901	3.52602332131273	3.06755110843039
H	2.03768267642709	2.49292600000330	2.10263643049845
H	1.37700834977076	3.97107279237622	1.40687155980378
H	-0.21115482465790	1.45082461575081	2.19762756835145
H	-1.53943459418121	3.59601885999909	-2.05460464289126
H	-1.14526650014467	1.66709990790678	-1.64233395644473

TS5C

Mo	0.04544309530266	0.58002547594405	-1.34240223172811
F	2.67745536340299	-2.17447392096764	-0.12056860199593
F	2.19723825933848	-1.81426593258499	1.95455355628210
F	4.24848344880612	-1.64705228652816	1.27407700108287
F	3.64180063837457	0.60102158265626	2.78970062191223
F	1.49203531267695	0.73872351127761	2.56932768352074
F	2.75873434961319	2.24636403950147	1.68624278325122
F	4.15893962462397	-0.21720902400023	-1.27737866913458
F	4.99948043886504	0.87062153281456	0.39690373645551
F	3.51377529213815	1.80226505675534	-0.87206204895975
F	-0.54193233170505	-2.85256740758906	1.43942916888147
F	-1.06671144924350	-3.37609140917892	-0.58689881368277
F	-2.49809289786367	-3.68614545380284	1.00371458889368
F	-4.45375844237946	-1.84918221227253	0.30659956184724
F	-3.74070653797696	-0.24371584058186	-0.96134036733742
F	-3.34702709624053	-2.29341505094301	-1.50568341552975
F	-1.12583352454563	-0.29723949320414	2.21723294526983
F	-3.08641536292335	0.37253540484665	1.61148005746196
F	-2.85982339280893	-1.53412208606900	2.60926820449722
O	-0.80499150189908	1.16400857868309	-2.66266983118978
O	1.51782461144469	0.20194683560686	-0.01953501367647
O	-1.17326686241433	-0.71503430503206	-0.41854140800053
C	-4.85505603538607	4.35462053604422	1.16648278022611
C	-3.81530510708644	4.40032080831779	0.34385320972685
C	-2.46368406238611	3.82629547619026	0.63554837023395
C	0.42770921419875	2.88890061236323	-0.65335855513845
C	-2.10263743688942	2.67795735302637	-0.32536804261065
C	2.65475309534098	0.92701495575527	1.94885822990102
C	2.70472095158688	0.10144807907012	0.60951688248380
C	3.87517132743866	0.64881086133196	-0.28722711657544
C	2.97078760502258	-1.41343989632595	0.94419050966897
C	0.46111974767302	-1.51029978481664	-2.50218700004730
C	1.46531618732028	-0.80837957207258	-3.14441990137621
C	1.32911880023770	-0.29760285146548	-4.55142966886387
C	-2.07046170774676	-1.38949502126953	0.31179311209435
C	-3.43526316770486	-1.44868309896443	-0.46629047225687
C	-2.29499049116977	-0.71006009562402	1.71786200840926
C	-1.54656829128808	-2.85830116535438	0.55236321771090

C	-0.71518948763486	2.14105372956821	-0.06473173699957
H	-5.81488260849713	4.77458985917059	0.89374302466864
H	-4.78263529226564	3.89305490814652	2.14524648050978
H	-3.93316458596956	4.86527630484283	-0.63249577898275
H	-1.70469888424255	4.61327635365154	0.54998651971238
H	-2.42892897128262	3.46117414870555	1.66521293741551
H	-2.16704597922453	3.02457354687634	-1.36010179046407
H	-2.84073372338962	1.88780684116166	-0.21578673401294
H	0.12418608106681	3.72069464065441	-1.28564603970428
H	1.21877391337101	3.14970278149977	0.04296109105292
H	1.05306816336950	2.26005568961906	-1.41859416406153
H	-0.55118884101506	1.82359542743877	0.96075066565684
H	-0.49503773724123	-1.66475662460037	-2.98396934629974
H	0.69747727174273	-2.14695549251185	-1.66496287182217
H	2.47607644417824	-0.94269191001751	-2.77522085535210
H	1.44357105090420	0.61196139584246	-2.39060521184065
H	1.61719853318805	-1.10103729790360	-5.23481564531924
H	1.98207648098144	0.55318727204416	-4.74266990642950
H	0.30119650821325	-0.01233636833641	-4.76977770943480

TS6C

Mo	-0.34103247915475	-0.98694624825044	0.01702169769864
F	2.39117858220766	-2.32337609923783	1.37100148674548
F	4.41332950088047	-2.11784894913358	0.62253877964075
F	2.83817632984179	-2.80226305478670	-0.68876733890993
F	2.31346457846905	-0.75780981927553	-2.40911197071070
F	4.41127126343900	-0.74460700183643	-1.85732438596997
F	3.26479205992853	1.09466233800978	-1.84393486781745
F	3.10300785156644	1.74392856127195	0.77275523797563
F	4.88539643594051	0.53484115257379	0.58776410186536
F	3.42811639022379	0.10720468467357	2.14145427804116
F	-0.41067166302450	1.74587006582313	-2.01154593711267
F	0.94016811048627	2.84287901155119	-0.73177336416155
F	-0.82133805285785	3.81937693252904	-1.52088268214199
F	0.47646490558353	2.48762023052627	1.92023811440705
F	-0.58829942038406	4.26185856533776	1.27822363623058
F	-1.58632320800193	2.76312230738762	2.49131532029866
F	-3.03822642112315	3.70805100883315	0.15010392352917
F	-3.42638570327326	1.68021570179617	0.80463999206712
F	-3.03996756983349	2.08187467633501	-1.28200952938142
O	0.06064338506609	-2.35169451178551	-0.87670465081640
O	-0.95335510313297	0.84875113857504	0.41897318065694
O	1.46426092574508	-0.17989836861128	0.05131749409085
C	-5.13057753266601	-1.85626612840157	0.95489681066430
C	-4.97813762502095	-2.40140028490719	-0.24590006156096
C	-2.16494415557575	-3.74927580715557	2.89130947141461
C	-0.99729298535754	-3.30164301237288	2.00924067348390
C	-1.03652923446712	-1.86835865823226	1.58810256747762
C	-2.35944799473632	-1.04204813268686	-1.11638094636754
C	-2.89575604923670	-2.33217388890330	-1.72025398339395
C	-3.75627125916544	-3.13097190285723	-0.71691787268426
C	2.78509631635765	-0.46703086407884	-0.08848318653931
C	-1.16340593954770	2.17102084112228	0.21284388048028
C	-0.34405147050554	2.66776749522654	-1.03358310701659
C	-0.69954132180555	2.94565812433735	1.49858829441712
C	-2.69567372408565	2.42939161230933	-0.03022408979000
C	3.12344835249653	-1.95525927053296	0.30582964060855
C	3.21193906353140	-0.21554749498119	-1.57825523979262
C	3.57418755135780	0.50313203036049	0.86590422734913
H	-6.03089765076381	-1.32042458426821	1.22638044080017
H	-4.36250876469227	-1.92519151664296	1.71682889365476
H	-5.78369785082031	-2.29869721522526	-0.96698270943163
H	-4.05265516788046	-4.06739441606001	-1.19861434372293
H	-3.14050776367826	-3.40927287254788	0.14085587403636
C	-3.66708426229721	-2.01554207482339	-3.00731320828354
H	-2.04695158193901	-2.96571293527036	-1.98712384916344
H	-3.11168364364318	-3.72601135544940	2.35196813565393
H	-2.00412985905614	-4.77137136108990	3.23634237259609
H	-2.26035750316031	-3.10868724634943	3.77020500265872
H	-0.88351696326416	-3.94244386599964	1.13369085495403
H	-0.06187308296283	-3.40528782552888	2.57352429881703
H	-1.41163618134157	-1.16448873754827	2.33563603642532
H	-1.98933778242624	-1.59220536190068	0.37515092546384
H	-3.14233974857545	-0.44354652006864	-0.65930329455265
H	-1.89636772519961	-0.42613789288155	-1.89069362732490
H	-3.00899286568793	-1.55856266784279	-3.74795357631996
H	-4.48975604280813	-1.32236746927022	-2.82237629269819
H	-4.08260914996834	-2.92674100478470	-3.44354152853863

IM4C

Mo	0.12211644346703	1.09376825910039	-0.48155006469537
F	1.05783059524778	-2.14542023616983	2.04571199032224
F	2.84794608585470	-3.16916585505529	1.37784612557384
F	2.99622010009599	-1.30069149432652	2.46971096897012
F	2.65338509872152	-2.89688602313926	-1.42427006345416
F	0.68246852810480	-2.84548201574635	-0.52924414961155

F	1.25045261185661	-1.33272367956545	-1.96199721942693
F	3.97445743835256	0.39222255564108	0.66625930423271
F	4.58873598399701	-1.55107335916459	-0.05845812626855
F	3.64607905940712	-0.13150493103288	-1.40403964773311
F	-5.00743246249396	0.06755007004534	0.21263705730934
F	-3.72917444006705	1.43810818705056	-0.87302813329738
F	-3.66447052103651	1.37418775036444	1.28805744810258
F	-4.02907024278890	-2.39962053835295	-0.34797463174209
F	-2.06383687426198	-2.34312531766036	-1.25150914938425
F	-3.62352568970680	-1.05684060323472	-2.00557865633814
F	-1.72360184249206	-2.21911686890746	1.42727470552984
F	-3.53325149087893	-1.30323372996396	2.18656606359380
F	-1.65183249093257	-0.22463842155236	2.25100940172166
O	0.25469748788476	1.73283949453005	-2.01452199767027
O	-1.49779036075414	0.05204809168818	-0.40704327752381
O	1.36063108769686	-0.11455960110359	0.39243706067335
C	2.28292163934060	-1.96838351327205	1.55453535775464
C	1.70151966179591	-2.09361814395741	-0.94774948486358
C	3.64473543394246	-0.61246290435479	-0.14996118676820
C	2.22063889615669	-1.16219659366695	0.20742483592575
C	2.47431929974162	3.11753473489687	-0.29283942003687
C	-0.99051660709009	2.62468685898368	0.59202383263134
C	-2.65803300351132	-0.46152621557946	0.10104880532973
C	-2.39727293872054	-1.07058928431751	1.52228022635851
C	-3.10813628634364	-1.59714955204722	-0.89448920106920
C	-3.79273292805281	0.62406578188757	0.18325740323678
C	0.07932198664149	3.69673088840153	0.66868681031745
C	1.23581921022324	2.71692011619499	0.48644617949210
C	3.40733917811325	3.99304029968484	0.55228980777914
C	-0.06973639230994	4.75031133830972	-0.42165665123925
H	-1.23185108178258	2.17232483140170	1.55302570380213
H	-1.90187276028201	2.86284397042619	0.04793905429500
H	2.89759641711577	4.89464899514030	0.89738234570100
H	4.27983791541267	4.30016380529021	-0.02768040388070
H	3.75984594740414	3.44970930403963	1.43110397970979
H	0.10733462320282	4.20419983332113	1.63915341003785
H	-0.12532489704823	4.29729395511807	-1.41307410718425
H	0.76372223299461	5.45432113499305	-0.41753868984196
H	-0.98698608318335	5.32000419717279	-0.26138542913542
H	2.17792484560854	3.64762728828541	-1.19860669671142
H	3.02762387053668	2.23925455268485	-0.62621024103221
H	1.50140771482015	2.26339258751827	1.44509875050792

IM5C

Mo	0.07129342838151	0.77671514639242	-0.89256628736488
F	1.02119338309183	-2.41051546501926	1.91830514666280
F	3.06792195779648	-1.78731796710705	2.26394132385578
F	1.49111784576648	-0.31136151272040	2.08996746910391
F	3.06844886049921	-3.63425671538231	0.19264656906870
F	1.06027476588505	-3.41936849605799	-0.58658887179926
F	2.74557220163932	-2.79307235950253	-1.78203422297040
F	3.58988429508742	0.46110871560141	0.56800835461861
F	4.56383577096124	-1.35891471427811	-0.08097391977988
F	3.45383059963706	-0.15212059169953	-1.49830447078839
F	-4.97589778008219	0.43999079213851	0.63537538244765
F	-3.86375506772138	1.67733025180653	-0.75328029031272
F	-3.24841125243754	1.56014125633502	1.31285482933392
F	-4.42442958203124	-2.11757087885975	-0.26392430360894
F	-2.68861352910530	-2.20203555559848	-1.55916386278533
F	-4.23828695559202	-0.74634280048542	-1.93586667077329
F	-1.78275346756913	-2.24118610169654	0.98077717340118
F	-3.28136473200673	-1.19626156669685	2.14534121516076
F	-1.33715127519304	-0.29415358862809	1.80248443884879
O	0.58177973686476	1.41806556798723	-2.33787885114433
O	-1.73797695975463	0.12208052508257	-0.78024314537066
O	1.08000598695145	-0.82360944374714	-0.55345536367338
C	1.93721656979781	-1.48414927968769	1.61913378973233
C	2.26471197309908	-2.85058528186630	-0.53442495400274
C	3.47542768668478	-0.60085254683056	-0.24168171007002
C	2.15726311805624	-1.40152230119119	0.06897535051086
C	-0.45307718420347	5.06840225574344	0.47295857597092
C	-0.46161962647687	2.61654556213994	0.04811665982399
C	-2.80554064481631	-0.34808525064912	-0.06452374852918
C	-2.30807367549349	-1.04467068754816	1.24715516568457
C	-3.56074152702905	-1.38191321696336	-0.97334950039100
C	-3.75470879585969	0.85229853533140	0.29027182387227
C	0.37932981227303	3.88583137076225	-0.04104546444992
C	1.73161348110597	3.80107466397250	0.71315885325043
C	3.93678012366203	4.45203781833982	-0.27538691028280
C	2.90449199768531	3.62188731566589	-0.20160886515842
H	1.22115592397579	1.34931861988094	0.15023902009026
H	0.09458689591145	6.00374647213399	0.34303123952651
H	-1.39970682513427	5.15347445137894	-0.06408794620315
H	-0.67695025784659	4.95231069760008	1.53657152828374
H	0.59218660385503	4.06623517858666	-1.09818166656189
H	-0.71166218083755	2.37797133809397	1.08588011295313

H	-1.40081190005574	2.75763671456540	-0.50714405083456
H	1.69289829312225	2.97698983710491	1.43407526546319
H	1.87910594158833	4.71084088223797	1.29873355836034
H	2.87766872788734	2.75689635738605	-0.85658273432179
H	4.00342462896531	5.33248466651181	0.35474712210228
H	4.75426260937463	4.28104132943614	-0.96418215695001

IM6C

Mo	0.38067013058114	0.51307322855325	-0.67989430924411
F	3.51318297271022	-0.76542773287828	-1.45883385221098
F	3.68873025885300	-2.31880114722336	0.02805703204828
F	5.19478393282687	-0.76319341460050	-0.08795697610895
F	4.42446140806461	-0.98896573027261	2.58406594482516
F	2.44887524873690	-1.84408399102334	2.36866991195663
F	2.66693650937121	0.16318437264065	3.13328298374491
F	3.86819753381710	1.72971387755164	-0.46331327214599
F	4.62610926752439	1.50982281826390	1.54925908315596
F	2.58296314568745	2.15723224473445	1.22052159945037
F	-0.52869329679667	-1.99451671706234	2.75229124141815
F	-0.46881159123399	-2.95209957621710	0.81748035649247
F	-2.16733272821318	-3.26875207593934	2.12635929975180
F	-4.20375457207911	-1.96903764296083	0.73542025126671
F	-3.45136361984625	-0.57340408260637	-0.74038996680569
F	-2.68869012819332	-2.59191947070176	-0.68425872200871
F	-1.60370616105279	0.48599269397971	2.79961491897801
F	-3.41632643425140	0.70631244947326	1.64802422124180
F	-3.24518024227857	-0.89427789017950	3.10165187425210
O	1.30953822644642	1.41492236974103	-1.75192319764619
O	1.71475356496887	-0.23518476836265	0.46128904723687
O	-1.02984755016961	-0.41061985481159	0.37526129773244
C	-5.22369089601882	3.39183627543103	-0.33681084323324
C	-4.05923419086574	3.85766206199132	-0.77140462486881
C	-2.74332433842037	3.58577588502154	-0.10829044669416
H	0.12225752112342	3.10004856432394	-0.11098557931417
C	-1.85327575206568	2.58260259956504	-0.88976180283196
C	3.15520740472566	-0.72223646000010	2.25469907232553
C	3.04053830278935	-0.14606875854818	0.79530356161750
C	3.55065548720493	1.34292447027738	0.77495128071752
C	3.88666025210577	-1.01602281381755	-0.19777064522694
C	-0.31493080527497	-0.72232639621564	-2.26444903150922
C	0.52465071691201	-0.87965473373153	-3.52180163061283
C	0.43273191010056	0.28177228870087	-4.50552678119518
C	-1.96425214024327	-1.10621993741081	1.05497845047874
C	-3.10673474197872	-1.57376311691646	0.08268319360032
C	-2.57375460154855	-0.19564303137973	2.18175944509401
C	-1.27554269525951	-2.36216746676093	1.70595603763170
C	-0.60670166843562	2.29239666514801	-0.05173816156093
H	-6.15015831586863	3.60499953515618	-0.85488722858534
H	-5.29164954712824	2.78150102864614	0.55710999684816
H	-4.03926335653489	4.46734186617100	-1.66972168261168
H	-2.18088810162835	4.51897230353415	0.00666684236464
H	-2.92020108119703	3.18820511821459	0.89202094807607
H	-2.44642167626613	1.66908832811226	-0.98655709765270
H	-0.85713266414510	2.09422683566234	0.99007349127886
H	-1.29826992083553	-0.27250845607525	-2.46293505323418
H	-0.48281178260331	-1.67724714014783	-1.76585199391093
H	0.18780884876598	-1.79727086058646	-4.01871960128379
H	1.56821559056831	-1.06025313447031	-3.25068457609547
H	-0.60509534272900	0.46229593736673	-4.79729237747749
H	0.99912165384452	0.06247645810083	-5.41206897669474
H	0.83289381668112	1.19960049401305	-4.07752781922530
C	-1.50952428498884	3.10164463477640	-2.28732556141749
H	-0.95706233600723	4.04244263800919	-2.22248738003677
H	-0.89012995095278	2.40184508806949	-2.84626206632037
H	-2.41331482029853	3.27756253967078	-2.87030015782039

IM7C

Mo	-0.09841651277755	0.62742044913707	-0.68711384361342
F	3.43251456031005	-1.11077243903232	2.28374313890111
F	1.36933692899644	-0.47011743395988	2.09156526623317
F	2.97091377905092	0.84707313226360	1.48763563046481
F	3.48254538391341	0.76234721948293	-1.16100347115687
F	4.84538889379325	-0.58118405297257	-0.13934134474946
F	3.80258903072345	-1.22990936199926	-1.92700984472527
F	2.09166463955427	-3.11884777302428	-1.02448983618311
F	3.71620881236018	-3.07704581329930	0.40567780164660
F	1.65482134707242	-3.01527809065202	1.08704152295981
F	-4.15267187546620	-2.25354290847889	1.37687000062864
F	-3.89672900707676	-0.22859753379921	2.11222265360763
F	-2.28469528757586	-1.65171884922411	2.29844991968753
F	-3.34046512641990	-2.66811266164965	-1.29694364423446
F	-1.64901497601883	-2.94605696323749	0.02754940228656
F	-1.45396478527516	-1.67669800874640	-1.70813051724333
F	-4.99434320397303	-0.54163903769805	-0.64626788700408
F	-3.41309505971035	0.18382431857932	-1.94604977180854
F	-3.89894634987313	1.25732909419335	-0.13805110155032

O	1.31522597129562	-0.67522037161695	-0.55136183843264
O	-0.24322344584240	1.24508978960846	-2.22951286781160
O	-1.61690546103526	-0.01383567728351	0.34911273054303
C	0.71573583121876	3.57289457945311	-0.12285862873362
C	2.58003426873528	-0.43632358626788	1.51197589004942
C	3.68704273691401	-0.51277198105434	-0.80646129195981
C	2.49745699509510	-2.59782808656479	0.13178780543946
C	2.48649690289593	-1.02738742497464	0.05984721291120
C	-3.27735764978970	-1.24762490805919	1.50595792563522
C	-3.79090007026914	0.03622854852371	-0.66565467318987
C	-2.30269650164154	-2.06105976071648	-0.72605529338468
C	-2.71431839811783	-0.79872755088043	0.11239978465169
C	0.40070460575793	2.16513175321631	0.25696668943564
C	-0.34999876430133	4.51728240927345	0.49206746595915
C	2.13527559778731	3.94622162577557	0.32343192024902
C	-0.21837805552791	5.92499778436960	0.00048530779383
C	0.03850413908762	6.97866720175058	0.76425258852995
H	0.65378016563898	3.65531775941758	-1.21135510385023
H	-1.33959626137616	4.13226329623580	0.22743808645748
H	-0.27249165406452	4.48898674573829	1.58265564352603
H	2.87416825518851	3.29312657819726	-0.13945846109158
H	2.23549318840419	3.86142066439097	1.40789721619599
H	2.35415356820105	4.97685002421187	0.04350924098829
H	-0.34463268905847	6.06386124353434	-1.07072168219972
H	0.17348421112699	6.88249955368194	1.83598930330716
H	0.11601916703309	7.97547379635454	0.34914469084057
H	0.44576215503628	1.93516270780195	1.33044626399362

TS1D

Mo	0.10191642092103	0.74535619005258	-0.55196205806397
F	4.60059991612422	-0.99673310636244	-2.25141435444254
F	3.70005641258499	0.78437889990795	-1.40189603492386
F	4.51254782252647	-0.73149016100460	-0.10012571746528
F	2.14381589241303	-0.24298768276245	-3.36993981562619
F	2.23840493280513	-2.39827975358456	-3.31537407592955
F	0.48525131193824	-1.36494172845254	-2.55752264509292
F	1.48996023839120	-3.40350828984651	-0.84440828732380
F	3.63514203805155	-3.34086790460323	-1.16267493485162
F	2.79198835692686	-2.65316986687238	0.70736725768857
F	0.02765502169235	-2.70322773806412	1.61363408085245
F	-1.88801548275111	-3.53860753889075	2.18105106498132
F	-1.20008950838152	-1.67866606041406	3.06175078319572
F	-2.48101597814623	-1.80681297848498	-1.53456949096721
F	-1.26287824577524	-3.36767126521891	-0.67782550258927
F	-3.36492762512362	-3.24098832122776	-0.17176183801813
F	-2.90632957230978	0.16326724642025	2.08535520461359
F	-3.96494301240056	-0.43768415871444	0.30292750236570
F	-3.96060098058602	-1.72587148447630	2.04615485172029
O	-1.03253657060878	-0.61102427997747	0.36902327752370
O	1.63615118969120	-0.53768187075295	-0.33867937505892
O	0.82616482078190	1.75243621905072	-1.68684871355060
C	3.84256753744846	-0.53719859387164	-1.24517467798257
C	1.83697417932011	-1.31998379139139	-2.64078310379122
C	2.59903063792247	-2.68922084450096	-0.61097818136864
C	-1.22799833911404	-2.40117669162290	1.92654199853209
C	-2.26211543455122	-2.52257610692826	-0.42135950886201
C	-3.21124349850487	-0.89443148853755	1.31848366767763
C	-1.30663169385142	3.64759149793344	-0.35579962074697
C	0.14767622764590	1.60685724962062	2.49682260900476
C	-1.81757810115932	1.42051092783960	-1.43220086105207
C	2.43617750039457	-1.23455142571361	-1.18500380541840
C	-1.89795457902542	-1.56686536968078	0.77308449698583
C	-1.75987039130032	2.28127707745641	-0.30951512375110
C	0.99518711406604	3.35495535385620	0.89833187226998
C	0.78782611860176	1.97029833647563	1.17089097199274
H	0.72806357927116	2.02060657075912	3.32717024659023
H	-0.86467619472046	2.00678867752692	2.56733660964864
H	0.08497309849186	0.53066053276451	2.63535442529338
H	0.89476104277294	4.08352156012477	1.70047349581212
H	1.68215669944109	3.62526799447714	0.10425746901160
H	-1.05300198610784	4.03210383768262	-1.34033862897516
H	-1.86674010008662	4.35809241657843	0.24888961285367
H	-0.21378073802367	3.71415399023779	0.22714094954696
H	-2.52748427773065	0.60739197444156	-1.40394387894392
H	-1.61663113949970	1.83087497502538	-2.41316821272028
H	1.73443062352015	1.43938650675071	0.97049238079569
H	-2.27243528398631	1.97044046697515	0.59073361855957

IM2D

Mo	-1.75058386147139	2.91178781012986	-1.27674969326529
F	2.71127599211220	1.91114529315292	-3.44185905428949
F	1.72324456672315	3.03139110239953	-1.87339425961644
F	2.23727337916209	0.96132402686162	-1.55028826396922
F	0.43871178322817	3.49585985693297	-4.20902323987572
F	0.66406948172307	1.70452316260907	-5.39740205161056
F	-1.26171287635519	2.20608779218191	-4.53974992103137
F	-0.67170896064348	-0.44847826156979	-4.08545839754075
F	1.49771366209099	-0.48445710096494	-4.04734094484599
F	0.37298479777966	-0.86051159119776	-2.24006868292971
F	-1.19213800504342	-0.68000319654273	0.16373114879031
F	-2.83724503593531	-1.91967750323900	0.83140750592546
F	-2.32931375963638	-0.10269682730630	1.90351498932143
F	-4.24193751197786	0.18474357747395	-2.45950668798252
F	-2.62672369837172	-1.18490017587442	-2.04304957225567
F	-4.61784675041734	-1.60698838276679	-1.30215858030458
F	-4.48581194446982	1.41472299291713	1.50658191491932
F	-5.63363327277685	1.00030934223018	-0.27412650798651
F	-5.23234830098713	-0.58479289817914	1.15268585031854
O	-2.79193444523288	1.34861799131947	-0.56739071756224
O	-0.56069387979496	1.57707678475482	-2.04896508850310
O	-0.90983318267469	4.26657534252092	-1.79821115915182
C	1.79862600312454	1.83423474209727	-2.46586152028521
C	0.06226526269949	2.21766016769751	-4.32001383086726
C	0.40582024482442	-0.13330524174679	-3.35430962319572
C	-2.41995742714334	-0.65677616979873	0.68261806953649
C	-3.72736683629919	-0.63303061863595	-1.52869960940106
C	-4.70982270549758	0.48979894917552	0.55954605473759
C	-3.48313205409628	3.46391372598126	-2.45675963544132
C	-1.01140598200005	4.83588686818104	1.11831037927728
C	-4.06511527321874	4.24440050118273	-0.20357435375052
C	0.39184161088238	1.40356997690675	-3.01175819782105
C	-3.38565515852078	0.18486683089591	-0.22748092590307
C	-3.69723262786534	4.50728287216471	-1.47626743333881
C	-0.03296339083316	2.51499494920568	1.02192696242174
C	-1.27406370128397	3.37627062387478	0.80624133475204
H	-0.73309881243849	4.93926256633365	2.17322128239342
H	-0.18974753732151	5.22409382274174	0.51596093597553
H	-1.88089035566167	5.46645570234142	0.94331122643838
H	0.23852629047931	2.55952732400982	2.08299258840202
H	-0.18405589804379	1.46597266243972	0.77590231464706
H	-3.20923667764394	3.77462278908295	-3.45730215531846
H	0.82029693657201	2.88889323577490	0.45544442230326
H	-4.14961956072183	2.61013459085272	-2.42332118942457
H	-4.06242734040028	5.02097347942850	0.54764396037988
H	-4.44735521272128	3.28015795563849	0.09038157663589
H	-2.11062047058220	2.98656643326203	1.38302061139839
H	-3.34574240831939	5.50338212306864	-1.72007251410585

TS2D

Mo	-2.12233131945825	2.93228723458593	-1.51871627609153
F	2.52518303393662	2.46746384494307	-3.50740586716725
F	1.10869264545396	3.73722177737964	-2.47451338755220
F	2.23853082433807	2.27475609222959	-1.36591457479331
F	-0.04674830823095	2.74827073754484	-4.71517974954563
F	0.81521686791704	0.79634414596380	-5.06601311223058
F	-1.23174857863111	0.97458781300662	-4.37725835237554
F	0.20089676063536	-0.93814440307736	-2.90029655030764
F	2.23865651596890	-0.20088359744745	-2.97273044585253
F	1.13822484196622	-0.16944582901871	-1.11203038409991
F	-1.42827521919886	-0.81353733220557	-0.12398273209820
F	-3.11570250124789	-2.16044579656393	0.03429286779251
F	-2.82823638160608	-0.58297583085654	1.50068356037049
F	-4.05326736557771	0.57190845875479	-2.95234255003928
F	-2.57607111732806	-0.95722455607277	-2.57203908890127
F	-4.66932442808833	-1.37486134288272	-2.21719295695892
F	-4.93021737415002	1.08484966379874	1.04679209578671
F	-5.85250103830539	0.81806913958655	-0.88738953585097
F	-5.50704653666384	-0.89560824113296	0.39092064946754
O	-2.93905897708600	1.31353958274779	-0.67023758103638
O	-0.57417013013342	1.65698022720953	-1.90516533998102
O	-1.76584710940338	3.97010560413355	-2.77691439108518
C	1.60565441790023	2.49357347785699	-2.53658539295448
C	-0.00226455093306	1.48924079633736	-4.25912066085731
C	1.02632147813112	0.00603053519117	-2.43329760349925
C	-2.71612894820051	-0.89273971931411	0.19391892707904
C	-3.71792943756359	-0.43568590792160	-2.12965617837265
C	-4.99023403216652	0.26432493993351	-0.01782786237757
C	-4.20318563981589	3.66987873149957	-1.45635741430467
C	-0.32715415960388	4.89633908618254	0.30126957173103
C	-3.27981198609047	4.10369878514363	0.86146170727369
C	0.45570368345671	1.44107755524205	-2.74811555235914
C	-3.56300252612878	0.11320714331288	-0.66536381809285
C	-3.45012761295967	4.45425809938209	-0.52346834315070
C	-0.27009384852818	2.47166471454859	1.05404982068422
C	-1.00231765969250	3.55608137963877	0.30909566547945
H	0.04006667286708	5.18320823900074	1.29119033449236
H	0.53640643902613	4.81148996985611	-0.36402489030662
H	-0.95667660252604	5.68750997859934	-0.10280316322182
H	0.00862123858113	2.79716056398240	2.06119863122471
H	-0.83487992520974	1.54426548191009	1.10687951741420
H	-4.51537146580106	4.16278142062079	-2.36728904887454
H	0.65067807565580	2.25529399270687	0.50785537929599

H	-4.88130249971523	2.91358501265736	-1.09459746692169
H	-3.24599052252839	4.89391289392016	1.60953604785506
H	-3.71792823092728	3.17115508831232	1.20448132228678
H	-2.00384614693130	3.74648792491077	1.05710273495475
H	-3.17038531540265	5.46041242386289	-0.80261956192776

IM3D

Mo	-2.48080339469087	1.12857557688640	-1.93832230778764
F	1.97612417151380	0.59801953035846	0.48665047627024
F	0.34372822535440	1.93965592576066	0.03036613841872
F	0.10416762789766	0.44588595624600	1.56624925485023
F	1.09045723510571	1.03974955461085	-2.38855002180153
F	2.15831747294104	-0.77247956780895	-1.86506047982441
F	0.33186896431707	-0.88474252906212	-3.01612270980874
F	-0.08314503113721	-2.68907531206601	-1.09703660610706
F	1.34429181663798	-2.06481600291613	0.41138996083195
F	-0.77860993605738	-1.97164603868056	0.81501399103060
F	-2.74440836971375	-0.04683263454469	1.84210755187349
F	-4.64780107999251	-0.70258442371652	2.63939186554958
F	-4.27042008565552	1.40895397720486	2.29547040625332
F	-4.74547243297287	-1.52249850991810	-1.43083483870955
F	-3.58040928540574	-2.10437858772021	0.28964650513303
F	-5.74196317047604	-2.06473426570919	0.41956158037818
F	-5.95032938100020	2.03771632861220	0.25446801220694
F	-6.67623851561384	0.32703106103524	-0.84417154908121
F	-6.80727454355726	0.35635830338360	1.31780549302481
O	-3.77448013768798	0.75744009973125	-0.47154126515801
O	-1.23455615195611	-0.06544944461193	-0.95781642412810
O	-1.83616684813984	0.92514830576421	-3.47408547754899
C	0.64498790939576	0.67055361108516	0.36514321769711
C	0.93586817651721	-0.24540482913049	-2.01640270947995
C	0.14541381399042	-1.79308158340633	-0.12766745410295
C	-4.05498029539530	0.17032567852902	1.81481132869519
C	-4.67220930397995	-1.44213562233404	-0.08971763936536
C	-6.03972972455643	0.70045439361082	0.28181499150086
C	-3.95676466929963	3.71100629125612	-3.87883223109132
C	-2.29620546766506	3.58477261634510	-0.28730410948068
C	-4.58258975429560	1.36814493924900	-3.20165507902776
C	0.07243772149186	-0.32908483070517	-0.70414505549506
C	-4.60157793383964	0.06556222986031	0.34433381969138
C	-4.40170943043291	2.68133210400527	-2.88845440797831
C	-0.97988280926294	3.72054935822214	-2.43788443748598
C	-1.90301857888404	2.92572389451367	-1.57757005916755
H	-2.82393401669035	4.51907207644647	-0.51105323380065
H	-2.92697364579762	2.97063830740271	0.34849888269586
H	-1.39393953226204	3.85468986740855	0.26850708989666
H	0.01283067632446	3.69373395485593	-1.97532189109693
H	-0.88848516811636	3.32185601813795	-3.44429421631373
H	-4.84323261001258	4.12149562772571	-4.37275756543905
H	-1.28173742127921	4.77200066743914	-2.47403150059241
H	-3.31722391432352	3.27622076808656	-4.64673660653891
H	-5.19345846065608	0.72698193994444	-2.58772253233783
H	-4.32766954963431	1.00603355343444	-4.18917053209532
H	-3.43579503670265	4.54399744104749	-3.41116857175744
H	-4.80904501534384	3.04384420913062	-1.95060400539569

IM4D

Mo	1.18998247905565	1.48437753586438	0.59760458469192
F	0.98023626417062	-1.24270578405540	-3.25268290901628
F	1.30538959197974	-0.73987178616486	-1.17011864183899
F	-0.63830987238478	-1.41648578778033	-1.83313075558957
F	2.34325148891217	1.11436747517949	-2.98946054605699
F	0.96200388545013	1.23417754514027	-4.63838931189419
F	1.04950556169156	2.83891913333838	-3.18582509213313
F	-1.53828203422242	2.01865493398197	-3.85429695681645
F	-1.35685906234545	-0.10688888629560	-4.25055231212087
F	-2.39492122402410	0.58944919448560	-2.48649152335061
F	-0.31681771364086	-2.17747839454452	0.86420621450476
F	-2.11802900965918	-2.59424869487022	1.99213239412203
F	-0.26254446562490	-2.19118108258519	3.02270490011169
F	-2.74009078490894	1.23638845799724	0.78678383562679
F	-2.58429878911337	-0.75327819332545	-0.03724384877442
F	-3.74335039243104	-0.41392085410028	1.75762435830604
F	-0.46562735522485	0.34142940626816	4.10130836696026
F	-2.27610667754949	1.32452660641478	3.45299837328375
F	-2.34532553824079	-0.73461926713278	4.11342097037250
O	-0.29928101048991	0.41046389483993	1.44246485672042
O	-0.21789397044613	1.48085638244632	-1.27042146020573
O	2.40739789737363	1.98936283594014	-0.43689439938163
C	0.41447577296000	-0.67407317024797	-2.18020120754279
C	1.11775080446739	1.50153652668965	-3.32888066681029
C	-1.33890180425105	0.80926409686179	-3.28022719461479
C	-1.00312330625841	-1.84719304237845	1.95884097828579
C	-2.62363893364079	-0.06830848420491	1.10626952367501
C	-1.60904549192642	0.16040165161344	3.43608898369015
C	-0.47920984623425	4.04034684260787	3.28960189982626
C	2.44280490379838	-0.74542575647047	2.57572918456971
C	0.59680855257044	3.54123672585227	1.09341619242376
C	-0.00377850168597	0.81755774544179	-2.44000777997078
C	-1.32075827669353	-0.29968947137492	1.95529707878263
C	-0.53845060431843	3.60448969250584	2.03084020764603
C	2.28906958911467	1.81925240675513	2.50154370332050
C	2.72786475169086	0.59836368601971	1.96505990049312
H	3.32772408726347	-1.08222456108510	3.12623104312264
H	1.61457342444474	-0.70263092500908	3.27571608822563
H	2.22455567734075	-1.50771697300809	1.82540317279606
H	1.58804278411490	1.82053420268647	3.32857225831046
H	2.89306741855172	2.71193957942278	2.41418125539966
H	0.44449732177383	4.41930635776510	3.71234997652533
H	-1.34505062455947	4.01966167362737	3.93815063954374
H	0.33461878276421	3.94765761168742	0.11707858857631
H	1.45340993106115	4.09701266744138	1.46701000943245
H	3.62981569603455	0.62786033716876	1.36281271890812
H	-1.49008875225760	3.22701409384904	1.66883887487591
H	-1.01188765757993	2.68543199275034	-1.37365030678074
N	-1.63313540951671	3.60667284175616	-1.44372673079742
C	-2.94009830321078	3.54281226105343	-1.17476717685151
C	-1.04200087629160	4.74844109503699	-1.81222688073339
C	-3.72572122608760	4.67656158750810	-1.25462179275933
C	-1.77450135796535	5.91406962524401	-1.91282357924361
C	-3.13404205515928	5.87766470665418	-1.62513091722381
H	-3.32127013050766	2.57303431856341	-0.89853617687039
H	0.01552643680921	4.69026667611548	-2.01956369553704
H	-4.77995263905566	4.61350648539782	-1.02823247890376
H	-1.28516382443595	6.83053859078772	-2.20815223670218
H	-3.72828357035797	6.77943111308746	-1.69164848511775

TS5D

Mo	0.61372698372319	-0.42150071920165	-1.03355359611302
F	3.69516966055342	-4.25756230491984	-0.97664141089589
F	2.82118992742832	-2.54203186620241	0.02395901474898
F	1.78436357105324	-4.43582224314808	0.02567952663228
F	3.75703217392595	-1.86501936316262	-2.45108837681659
F	3.47173617659120	-3.58178206847298	-3.73020707401998
F	2.20557783881253	-1.83976140946472	-3.95959432386246
F	0.70399315886409	-4.16012124083001	-3.93338209078345
F	1.88451338096563	-5.51599231994469	-2.71347479356800
F	-0.01428670951066	-4.77506003430703	-1.99434267228160
F	0.38146912985990	-2.37343545179391	2.14176349280070
F	-1.44453707387773	-2.95028632427849	3.15335449047467
F	-0.87615555079247	-0.86263389072748	3.03761987631239
F	-2.35607804008175	-3.21078658595791	-0.85605454276611
F	-1.02579866198687	-4.13951971683466	0.57456888543642
F	-3.10368540721974	-3.80177682808146	1.08883527929547
F	-2.65872022521141	0.28243836889650	1.35140369569291
F	-3.79754835600454	-1.13676732940659	0.19066715407645
F	-3.61850776891967	-1.39007825989617	2.33447138551393
O	-0.86994176901660	-1.22996923449298	0.09626756780656
O	0.81303536529285	-2.29735569553579	-1.72204028729882
O	2.04183833578521	0.18418957718534	-1.66908901753985
C	2.54024580987909	-3.62674596141196	-0.72453821793290
C	2.82659063689726	-2.61019814412396	-3.06204416343400
C	1.08047494207178	-4.44031301745416	-2.67506887069655
C	-0.89024946713888	-2.03482963515950	2.35309205089446
C	-2.04401234907228	-3.28650174985750	0.43742553748219
C	-2.95877773348524	-1.02791022973516	1.22415926897431
C	-2.84826543381240	1.27529143318452	-2.26734415366082
C	-0.03850766147215	1.65671435217940	-0.12927085695288
C	-0.62975392048490	0.22685910901469	-2.70472201882521
C	1.78135611185047	-3.18901508923430	-2.02843828289682
C	-1.64713751846749	-1.86324864953051	0.98413933388785
C	-2.06915549758903	0.20003291130474	-2.39643391944528
C	2.26635256126205	1.38961072488544	0.95614514286868
C	0.88723992162729	0.85134214877576	0.67036201465605
H	0.35582134535726	2.32140793703448	-0.88991828733680
H	-1.08650673391387	1.39725088878589	-0.21855498572540
H	2.23643295697845	2.11618253467575	1.77743631415175
H	2.94244529489275	0.58892187747070	1.25945217285691
H	-2.45943480024718	2.27712824670661	-2.41645921073661
H	-3.89936582276907	1.18625508057543	-2.02558917553825
H	2.69407615916588	1.87188414026140	0.07805130897980
H	-0.35162933537833	-0.53538005209489	-3.43575254458906
H	-0.28941904450298	1.20462323789139	-3.03737764527807
H	0.43702356008837	0.37679000980985	1.53423539030551
H	-2.50774687602058	-0.78078378340459	-2.24593670649940
H	-0.30502713935274	2.56323628459596	0.93453786479119
N	-0.56655598634414	3.32573730787997	1.91558271072544
C	-0.09866152071344	4.57380321501720	1.93561725753608
C	-1.30010845322920	2.84267416246571	2.91998311907462
C	-0.36118068448684	5.41640387704374	3.00113564559269
C	-1.60090252883151	3.62830355676208	4.01946309022915
C	-1.12438437119351	4.93326056734465	4.05835897307798

H	0.49153002334161	4.87735994990005	1.08024016081503
H	-1.63681104623686	1.81965664576640	2.81992521742275
H	0.02548035918988	6.42559612851145	3.00146326640387
H	-2.19475885228401	3.22146147371491	4.82551690016303
H	-1.34443231380954	5.56886081502604	4.90594489581370

TS6D

Mo	0.50242860837970	0.29447243738010	-0.68990579545376
F	4.98135686834640	0.13684062424412	-3.01710542768945
F	3.76893413251913	1.05431913220056	-1.47373967598287
F	4.71473983767019	-0.84595947776156	-1.10307962019627
F	2.45076715621925	1.16776280285754	-3.85392220672336
F	3.02451917161481	-0.58000788544333	-4.98540747788710
F	1.03811214183592	-0.44434500447571	-4.13277652870210
F	2.19499797168920	-2.94137675120034	-3.58122761944977
F	4.31523508602902	-2.47681394527716	-3.59411132982866
F	3.34171351301781	-3.04422118930719	-1.75204042495160
F	0.88355652790477	-4.11291175366362	-0.93033127641532
F	-0.21296168918179	-4.96429076773620	0.73054511991786
F	1.23169825999673	-3.39013084650842	1.07667267122156
F	-2.80318904383223	-2.36622108824905	-1.28741069624008
F	-0.95086327089136	-2.66052082383861	-2.35770616833548
F	-1.91987671436415	-4.34070668247292	-1.39050253485646
F	-1.09682468913696	-2.76797727461799	2.42166040073779
F	-2.56282556338687	-1.64713849733040	1.30637445470158
F	-2.61309326447103	-3.80849907104196	1.27083179959867
O	-0.24078704609603	-1.49357789126916	-0.03818498528759
O	1.83093447076621	-0.87073091892394	-1.63387494041276
O	0.85342992266877	1.69320140152907	-1.54936029298172
C	4.09579146628844	-0.11386445328298	-2.04172183025604
C	2.33009267417238	-0.16440273525486	-3.91619741153097
C	3.18087811910047	-2.35899672616452	-2.88435741878469
C	0.29178434738184	-3.82278860009700	0.22964573276308
C	-1.63628149637359	-3.03594073371175	-1.27642523074951
C	-1.79450776136531	-2.75277880519885	1.26977083474287
C	-1.62632307101074	0.68665800615955	-1.12208649777674
C	1.79642030478023	2.28772146472237	1.23563150863186
C	-3.02385115150365	2.74264605193122	-0.84136006518323
C	2.81274527926188	-0.85482333665681	-2.57668090041713
C	-0.81763390964130	-2.71068878462239	0.03298929945129
C	-2.02787462999883	2.07430443829511	-1.42717930623522
C	1.38891944532083	0.00576629703731	2.20255437510605
C	1.03275880944473	0.98056006882542	1.08513143886142
H	1.61644900294063	2.72906509208782	2.22445049764639
H	2.87371878943827	2.10461141898318	1.17353190266252
H	1.53961530021580	3.01594189148270	0.46831929417286
H	1.17538104456288	0.46198124067443	3.17782937236737
H	0.86566556305864	-0.94368230731825	2.14093345540915
H	-1.66441690359702	0.07216098849933	-2.03215183616085
H	2.46334801182015	-0.20306609550365	2.18913243785584
H	-2.26459299143340	0.22203271271409	-0.37335732670013
H	-3.27399135562036	3.75508990007422	-1.13416561708286
H	-3.64536311201182	2.28614675766581	-0.07790749567730
H	-1.45900581230798	2.57271044998473	-2.20686050818582
H	-0.38738469119655	1.39845768749266	1.44493031580692
N	-1.30053936637925	1.90522198046616	2.16758406224746
C	-1.51496442099780	3.22183685812095	2.10784735913211
C	-2.02806869397885	1.12517308178322	2.97134515388827
C	-2.48888669968469	3.82289474857939	2.88690334610667
C	-3.02315959733537	1.65684632679663	3.77331231427291
C	-3.25330830993153	3.02748421071978	3.73174255305086
H	-0.89347265640016	3.77833511815442	1.42072400031102
H	-1.80143942601864	0.06833516367387	2.95211658391899
H	-2.64567360896144	4.89054688494590	2.82357761443528
H	-3.60187378364679	1.00669829830263	4.41442106185760
H	-4.02295709568958	3.47063891054431	4.35061056525882

TS7D

Mo	-1.41247572744504	2.79818263470985	-1.48890391782513
F	3.12250364928895	0.63469628091861	-1.84476601472205
F	2.11015273614031	2.19103909505350	-0.74034118796327
F	1.11887237267238	0.30179215579378	-1.08998243273629
F	3.07051115752787	3.42235005345185	-2.88153769932209
F	3.31535614832045	1.82421190968637	-4.32348665974984
F	1.86609518900104	3.39003710063338	-4.66800821821044
F	0.58859036698096	1.02418650895291	-5.12442127754246
F	1.75251213199772	-0.38482827927790	-3.96000698115277
F	-0.29298652098133	0.04761414623112	-3.41487179137203
F	-0.66173766455919	3.58877057841470	-5.66337533173198
F	-2.53328556653952	4.15527410925334	-6.58707735966144
F	-2.43076677518795	2.39560916184385	-5.32901207669457
F	-1.90810629944588	6.69576290473677	-3.31132903434314
F	-0.18947304801049	5.45760695202746	-3.72392044683483
F	-1.29087763665131	6.35260394628106	-5.35915598751895
F	-4.61477943318222	3.90359775314702	-4.58634431035260
F	-4.25597278381830	5.42394930892654	-3.09679992452911
F	-4.04257264149235	5.90879144412102	-5.19265642132443
O	-2.17009834214538	3.75324084068474	-3.08286653891906
O	0.26399308486711	2.62704297558855	-2.70603526336568
O	-0.53768892117699	2.88966017894837	-0.04935418867047
C	1.92901935236608	1.21765271346898	-1.64445145124804
C	2.40270151433491	2.61156045568218	-3.73232271780967
C	0.83836186409518	0.59639962213650	-3.88040309332827
C	-1.98020428179042	3.64610306978193	-5.47293760259789
C	-1.41162739687202	5.76863434192649	-4.16115808995676
C	-3.83601872459114	4.95038057777027	-4.27855500460535
C	-3.26915265881775	3.74252274831636	-0.40784496972709
C	-2.00753263127175	0.10248258309542	-0.24608009254320
C	-2.48108337831363	6.11613844203197	-0.02209263145337
C	1.29111298655530	1.79556773003123	-2.96331015754123
C	-2.33036829232366	4.49790610083145	-4.18944643403191
C	-2.70725323490238	4.80218065565953	0.34706231106182
C	-3.14702975686036	0.64005319821004	-2.44066313691025
C	-2.19614637457472	1.07886038419547	-1.36144754100645
H	-2.97971142492723	-0.13881743626020	0.20091089064235
H	-1.60121889116738	-0.83413333768040	-0.64325618138795
H	-1.34035432373807	0.47107625063666	0.52797146758439
H	-4.13701029620183	0.48107010897425	-1.99747310363617
H	-3.24772293130306	1.35191562343126	-3.25546142987899
H	-3.93442988743895	4.02124396098155	-1.21526765556916
H	-2.81847957771874	-0.31631445252352	-2.85809520765752
H	-3.64248976841396	2.92680101923534	0.20146332895212
H	-2.31429156587436	6.82992999313832	0.78061141730705
H	-2.98063541930263	6.50592335840284	-0.90382018431817
H	-2.32425921083476	4.51497215026591	1.32486905886388
H	-1.05127355811492	6.15208973325077	-0.41647501401881
N	0.09703352263969	6.37526961267027	-0.75987533911876
C	0.33921315055361	7.53092711298787	-1.38320590537287
C	1.06434053892044	5.48096223551925	-0.54105062346068
C	1.60750106648851	7.84027758812485	-1.83167206317266
C	2.35704274528820	5.72760192329791	-0.96899200660143
C	2.63016321452609	6.91959816069076	-1.62475617744567
H	-0.51119125722724	8.18351735895937	-1.52519246852582
H	0.77370385015728	4.56348310366366	-0.04641484851614
H	1.78651862499140	8.77575406821569	-2.34204431594277
H	3.12105108236342	4.98286067007793	-0.80681175191132
H	3.63046286614124	7.12822959126881	-1.98046268567060

TS4D

Mo	1.33844735844886	-0.23739964016513	-0.34616641018825
F	4.84512667997298	-3.42719781102579	-2.16325095980798
F	4.31851099253275	-1.41259896490608	-1.59263052671091
F	4.66335661467334	-2.89769291141231	-0.06733256859884
F	2.59619986957470	-2.11643378016558	-3.51093948496956
F	2.48315024524934	-4.27600004752538	-3.42135334044434
F	0.77793490468909	-3.07093955572805	-2.84931512719675
F	1.23320236969357	-5.01548460110296	-0.91336942384260
F	3.35666310314504	-5.39941681376454	-1.10017060430099
F	2.58086136375818	-4.44719344169543	0.67705060920288
F	0.16451974763800	-3.47809315510868	1.73089070941026
F	-1.95325806562287	-3.76534080774308	2.08312906146705
F	-0.83557951684859	-2.30916226417739	3.24280541871886
F	-1.62054096393988	-1.53432017562670	-1.39583178769462
F	-1.10568350261290	-3.49979280772210	-0.66124748861415
F	-3.08819229223439	-2.70960685767732	-0.31769057414522
F	-1.92364141650706	0.02270612594336	2.46857941552620
F	-2.91144546870003	-0.00983886134862	0.54834068152843
F	-3.43503736774203	-1.46574382555215	2.06702844937730
O	-0.16186815262246	-0.99756897717138	0.75576928990419
O	1.83706885988538	-2.23828128207292	-0.57769350730116
O	1.96429411060505	0.22905028807762	-1.81937363657090
C	4.13071230782031	-2.70130199108856	-1.28765573791227
C	2.11278461920804	-3.14391934339968	-2.79696456764010
C	2.44889652598157	-4.51434724334845	-0.65213928268078
C	-0.96777537870493	-2.85356642609178	2.01894747019111
C	-1.78207615952017	-2.39437168876262	-0.37460033963132
C	-2.40942898438288	-0.80041822327618	1.52996040349487
C	0.03886467713835	3.54692446012728	0.74935218210836
C	2.95864837088289	-1.02468030820078	2.16094377220784
C	-0.22380311616196	1.50608930694221	-0.67212578293204
C	2.60267649595289	-3.08068072716811	-1.29391943175663
C	-1.27324864033939	-1.73019868572753	0.96035326264463
C	-0.34289746113551	2.23612860922299	0.52544651584744
C	2.32278190352153	1.16165424813128	1.01037115091414
C	3.03478985559523	-0.06913086887278	0.99241028730671
H	3.74658779365010	-0.78379143498297	2.88216879411080
H	2.00058712288528	-0.94448955411716	2.67738556728111
H	3.08803190378884	-2.05841218742450	1.84936612017390
H	1.72208261488675	1.40804721492882	1.87835151814776
H	2.68522307310391	2.02539531280182	0.46666445596814
H	0.65937075258220	4.04679375133107	0.00909978641965
H	0.13544207890066	3.90570998124959	1.76900240715886

H	-1.03814589828488	0.83110510692133	-0.91335639944557
H	0.15852160252274	2.05320480211364	-1.52805752448908
H	3.94380854928129	-0.12791713875429	0.40680479620495
H	-0.82451346902375	1.72852463144383	1.35587553264388
H	-1.38153432247155	4.04219291962745	0.29546857589656
N	-2.46212996571265	4.36303095074054	-0.10030740434321
C	-3.40026764390605	3.41412864381551	-0.10372915177454
C	-2.70799563354299	5.59924168027667	-0.53586132559008
C	-4.67581881623111	3.68939199074350	-0.56235524723152
C	-3.96211404930393	5.94336575243976	-1.00439321215382
C	-4.95823959809466	4.97216515948653	-1.01677836632205
H	-3.09875736730694	2.44184639203600	0.26268608528237
H	-1.87920574675926	6.29423088901765	-0.49890620322869
H	-5.42582621085713	2.91161716510420	-0.56390850938111
H	-4.15220175584612	6.94855634389579	-1.35223910546951
H	-5.94791950315273	5.21423067648860	-1.38058928676959

IM5D

Mo	-1.72978584208198	3.09873698901556	-1.52649871030710
F	3.23976200869844	3.11588012033376	-2.68375431539603
F	1.36823261400744	4.10184575352477	-3.13298927753451
F	1.89670545226434	3.67197246155941	-1.08194595375764
F	1.72175181662755	1.96242403084602	-4.78705730152183
F	2.92012891442186	0.56803681519470	-3.64135123848909
F	0.85946967472262	0.10442051081496	-4.10610415091261
F	1.40097905893537	-0.38420891012065	-1.45844346347519
F	2.91433533843857	1.05338747349598	-0.88854557697924
F	0.89701972037674	1.23846384016454	-0.12581871818672
F	-1.98025990275359	-0.41653059697693	-3.58853300735446
F	-2.95342059556574	-2.01109257854101	-2.48730136072921
F	-1.21258781541745	-1.03514331105202	-1.66303251595213
F	-5.35942085822376	1.30349817479983	-2.05666021122845
F	-3.79598632817371	1.64421033546352	-3.51204987663603
F	-4.80204801044346	-0.27218555206685	-3.42296433035863
F	-3.04910078517831	-1.08923871107024	0.32829415497935
F	-4.43126857359395	0.56709219293632	0.39181205814205
F	-4.93204848863815	-1.20895388129506	-0.74342650710838
O	-2.45767820183845	1.27744170638786	-1.22407183232409
O	-0.14507747303171	1.95503237455274	-2.50913808805534
O	-1.61498146912436	4.30675703503843	-2.74249793835203
C	1.94537301970979	3.18691852516238	-2.33121217743676
C	1.69040006726583	1.08835540071808	-3.74978749316906
C	1.61370706660711	0.91406011675143	-1.20792555453927
C	-2.32629558392917	-0.83246715621549	-2.35021138789514
C	-4.32588541993774	0.72584369664859	-2.66986003868903
C	-3.92936647097868	-0.38669288250660	-0.39651888743659
C	-3.74286379923404	3.76667623971075	-0.89124348331231
C	-0.40920716293640	5.22131236293852	0.18372497657659
C	-3.97329634068542	5.73704623828536	0.64597481239109
C	1.18491728841890	1.80631034314000	-2.43847779326887
C	-3.22574944907492	0.25335497134206	-1.64870095714341
C	-3.80192671538504	5.20457674436495	-0.56562706530437
C	-1.06656955585603	3.03631309192008	1.29265175540849
C	-0.96829872976995	3.84510615490156	0.03038834621264
H	-1.09042092129828	5.80566235657266	0.81145222990516
H	0.55993381993653	5.17972354976800	0.69013591537477
H	-0.29099479224846	5.73733777539307	-0.76580201514780
H	-1.71849945786281	3.56471203680686	1.99736358668632
H	-1.46688035455905	2.03466400196575	1.14247081793391
H	-4.38853847854274	3.53956103322458	-1.74275409782321
H	-0.07943163251866	2.94363092102208	1.75517203032967
H	-4.05417193645722	3.15280487957920	-0.04523352863951
H	-3.97492902516664	6.80887180627612	0.79977001457484
H	-4.12540321159440	5.11519547349394	1.52209221353140
H	-3.65731174991240	5.88203178420008	-1.40483395821060
H	-1.14988193552417	3.90939165172935	-4.17438950177510
N	-0.89293406727376	3.54186547664197	-5.13951459499335
C	-1.20212478463864	2.27425455359116	-5.42958943708469
C	-0.25005443563728	4.34221386551938	-5.99608524477631
C	-0.87565833539495	1.75415427495970	-6.66626953621484
C	0.10634344457181	3.87307754107786	-7.24266846611433
C	-0.21551119592872	2.56232927693556	-7.58185739032743
H	-1.67490288071858	1.70262575106738	-4.64822283580368
H	-0.03249407443695	5.34020719434949	-5.64538889644326
H	-1.12023502295423	0.72647982835525	-6.88974752525625
H	0.63038862028865	4.52044912193838	-7.93013495874420
H	0.05853540422883	2.17113785536402	-8.55273880283739

BF3

F	-1.59278308157906	0.28492246088611	1.60208584071191
F	-2.56487862428374	1.91290214459094	0.33742306147176
F	-2.41698052793036	-0.21629402289881	-0.46273252733700
B	-2.19190476620685	0.66056841742176	0.49233662515333

IM2G

Mo	0.20543037339500	1.27398910749458	0.43360200866899
F	2.23777537576818	-0.15105886055110	-4.13807666072901
F	1.53310273232680	1.58325448223983	-3.05591620296561
F	2.92280600565531	0.22486655473793	-2.11423042937591
F	-0.74023287216991	0.54839568083716	-3.96526860730114
F	-0.17197364751742	-1.49812142708968	-4.40086246938745
F	-1.61884706975231	-1.07328352779554	-2.84771900634432
F	0.10632905846207	-2.76023062753333	-1.66428764452831
F	1.88173168865829	-2.54565958954608	-2.88727744882552
F	1.90165513458131	-1.90853014796782	-0.82107983384255
F	0.02627803308103	-2.52557550082226	1.31945899612599
F	-1.49979079362948	-3.25126556352089	2.67199212998629
F	0.00220645217548	-1.84652742503754	3.36730249075903
F	-3.35149687044064	-0.38841929098808	0.30535842802563
F	-2.30821743221652	-2.25300910381887	-0.00431019260170
F	-3.78617701891184	-2.09163239128206	1.57215156757189
F	-1.43016088263455	0.38471387114982	3.87576005100373
F	-3.34169091445227	0.48258651105242	2.87721997053231
F	-2.81370660476336	-1.27180425618172	4.03172899087733
O	-0.90809946798146	-0.04058102669570	1.29472608302846
O	0.13326804991906	0.12649714554836	-1.26676577611841
O	1.04869622757749	2.61746696402173	-0.36319016736291
C	1.85710693217941	0.29421282380282	-2.92899165956327
C	-0.48205655006160	-0.64866262606407	-3.40790140155556
C	1.14848455299025	-1.95872277688501	-1.92704690471633
C	-0.77473192179976	-2.18174972597267	2.32361931699534
C	-2.80608100622837	-1.44115082593163	0.92581660450200
C	-2.33022969609741	-0.34107883011136	3.20353599445496
C	-0.91988812272983	2.82873267380292	1.59427027738025
C	2.27626375648605	2.59696095859447	2.51865267705749
C	-2.07908590069363	2.72189173192747	-0.62760168505762
C	0.65390727485271	-0.51953962932630	-2.32452381055752
C	-1.67872199542022	-0.96124330334492	1.91263890138592
C	-2.06660841806315	2.62058188583923	0.72208950772188
C	1.99675198005260	0.39689785162168	1.22518805520441
C	1.51794136649059	1.33719857105764	2.18966385041854
H	3.00386841531748	2.38859772118058	3.30997446145842
H	2.82346304937964	2.97511468656824	1.65619131869406
H	1.61982878982433	3.39089413424648	2.87758869605780
H	2.85365024217348	0.64609151704258	0.60931688070243
H	1.85213181068848	-0.65567896516908	1.36798480496298
H	-0.31904964082995	3.71485390161006	1.42778834851649
H	-1.10681570793815	2.65621457008649	2.64721952012173
H	-2.92263149262874	2.35133653701864	-1.19403036940838
H	-1.26716702184853	3.14742369076703	-1.19963132794126
H	0.95787244708104	0.92931572088692	3.02455424908306
H	-2.92652500161934	2.13358417555395	1.16842157660516
B	1.61764727668002	3.91305217495673	-0.79420972199720
F	1.37231777362945	4.86738781737077	0.21953822454836
F	0.85544967616657	4.35377090092766	-1.93374858782843
F	2.93828289419111	3.79482863439038	-1.08683050185154
H	-0.23274879502898	5.40078556235223	-1.33726325055991
N	-1.11522054142480	5.85501696225393	-1.01970541281494
C	-2.12936063703743	5.92372433032805	-1.89238607900177
C	-1.22514988359774	6.24139984163449	0.25660240997735
C	-3.34677080632730	6.42746121610361	-1.48970865004190
C	-2.42657746627461	6.74697323933757	0.71183873652171
C	-3.49651461264027	6.84017156102511	-0.16911863317384
H	-1.92791077196830	5.55506683630891	-2.88734550788129
H	-0.34125970906664	6.10921240196792	0.86105678907244
H	-4.16388325068576	6.48098269259724	-2.19365752589339
H	-2.51831799204530	7.05072429585600	1.74403411526494
H	-4.44773433425729	7.22653260853518	0.17214313893967

IM3G

Mo	-1.51637840773190	2.64903524039056	-1.21341690446470
F	3.27239358130683	2.41052510783976	-2.63970581516698
F	1.70164520392329	3.42318668537995	-1.54991834138416
F	2.40657494004453	1.50514501039788	-0.86785163466587
F	0.92970602494505	3.53756239245323	-4.11587189223118
F	1.94847302416328	1.86922190046901	-5.05126743371785
F	-0.20886010376483	1.85590795780096	-4.84664766163586
F	0.64478998446347	-0.56906868970104	-3.94793545800838
F	2.69963546477240	-0.15818039187281	-3.39008859016549
F	1.24386622953406	-0.66598190628032	-1.87457206423319
F	-1.42962222476526	-1.62514173056750	-1.92455176678697
F	-2.40227662628978	-2.59102303874054	-0.24490422254578
F	-0.83388252365527	-1.13790111680022	0.09711870856969
F	-5.03727593682056	0.28189544283730	-1.66044170660887
F	-3.33707483675845	0.05890618609710	-2.97739419485831
F	-4.25158691506076	-1.68956881924441	-2.08076068142525
F	-2.93941948259968	-0.58070153222761	1.73584934608840
F	-4.35746503830114	0.83372446970202	0.93666728072753
F	-4.68326015515292	-1.29155896575332	0.66679298848870
O	-2.26529990150429	0.91485767184529	-0.70253665593578
O	-0.18067912554786	1.55842643195268	-2.22368874877620
O	-1.16528025337349	4.18274336379946	-1.95413362472956

C	2.12965151703110	2.22777165640755	-1.96861612189613
C	0.93222969364522	2.20747666077701	-4.24385042861752
C	1.42047141668882	0.00221212823548	-3.01762423119505
C	-1.87447079248438	-1.43667310469310	-0.68238069824988
C	-3.90827813937040	-0.41526524356143	-1.86914700828970
C	-3.74638284780719	-0.34244411303830	0.69090727618261
C	-3.68707445630640	3.11988724306408	-1.44948476929135
C	-0.14644541333569	4.41134379993081	0.85309600171210
C	-5.21889435649924	5.06974066692437	-1.17258813875180
C	1.03098535675625	1.51429534378953	-2.83373623415056
C	-2.92563374576495	-0.26310838373717	-0.64837508038801
C	-4.19083071723738	4.46744900652352	-1.77373063320520
C	-0.79685265192983	2.13637360534619	1.65613959461559
C	-0.90273102289286	3.14474659662083	0.53304783761744
H	-0.44310562711904	4.78540540824711	1.84050334378675
H	0.92097924005964	4.18058320850203	0.91946657275088
H	-0.27182404211195	5.20020962795479	0.11973772384064
H	-1.17212091211922	2.58129204110973	2.58618270734043
H	-1.31789066632983	1.20631951435760	1.47408120627803
H	-3.74839052001068	2.50630689535713	-2.35998447534923
H	0.25916169441050	1.90550887084393	1.83059839168928
H	-4.29497176474687	2.63102093431290	-0.69217755061756
H	-5.55613928101514	6.05050034303473	-1.48275769629160
H	-5.77941961454278	4.58691797904351	-0.37744343532965
H	-3.69707738064225	4.98709727493493	-2.58635635637231
B	-1.06272254467522	5.68201061361979	-2.44987816081416
F	0.26701115415384	5.96403081822458	-2.52965336665581
F	-1.68909570435694	6.41092475897399	-1.42581700241330
F	-1.74808315539298	5.75318656242794	-3.62988546272001
H	-2.72015718607365	3.97192752971709	0.80393520698327
N	-3.34927334230682	4.58097521630893	1.38121666480676
C	-3.38506486463466	5.88632973482226	1.08942722024900
C	-4.13082073096018	4.00742717391312	2.30469237132221
C	-4.23207931700304	6.71069075197872	1.80884657626349
C	-5.00292122253870	4.78439935192016	3.03417029363848
C	-5.04514712524151	6.15614891972327	2.78616229036564
H	-2.73435373042874	6.21981073240372	0.29174148631804
H	-4.03203363115341	2.93774761144731	2.41822371858706
H	-4.25870630070706	7.76652975827748	1.58378065523633
H	-5.63454457002828	4.32660103256281	3.78099013757948
H	-5.71988135180387	6.78555500961242	3.35128420780177

TS1G

Mo	0.82582056840029	-0.64371262244830	0.69025425580709
F	2.39639331473827	-1.06392285704940	-4.18397482392873
F	1.98044548358638	0.26483608335291	-2.52306702162248
F	3.72619992727542	-1.00278989597408	-2.47321074938473
F	-0.27809142754030	-1.05026616942658	-3.27281289939357
F	0.29302016022814	-3.01888339153395	-3.94398877882107
F	-0.68337986641649	-2.77267527088950	-2.02776841894129
F	1.56558534635142	-4.45466302398521	-1.87624412273489
F	2.83671246746031	-3.69599156516254	-3.46546281591576
F	3.42437645431553	-3.46597042029359	-1.40036657750639
F	0.10715252380822	-4.34824382624956	1.89743605275212
F	-1.62550369265943	-4.74962995439327	3.13394519701934
F	-0.03062806644991	-3.48288941701549	3.87109764809354
F	-2.94453218910332	-1.99064538949171	0.37757859302888
F	-2.13752184061565	-3.99245803773433	0.38630995373386
F	-3.65921061482958	-3.43223822327037	1.82529712820666
F	-1.10579704542710	-0.97134387034635	3.94132496698185
F	-2.94407211113381	-0.78592594774800	2.82502739615626
F	-2.69325599000079	-2.41738022700524	4.22817046430658
O	-0.51433266307357	-1.80703971424434	1.48060963637586
O	1.51204483152052	-1.81344406748285	-0.79358578948987
O	1.67515127090247	0.86813909008444	0.63859788072874
C	2.44234062308722	-0.94876266817757	-2.84662513100430
C	0.21362975399472	-2.23328076921542	-2.86105011670061
C	2.37856042837611	-3.44633043773481	-2.23017742350486
C	-0.73931488914761	-3.81413183019178	2.77790887770513
C	-2.57209291837893	-3.00287241219507	1.17008546559110
C	-2.06184415580977	-1.67028002969834	3.31804615663792
C	-1.41808626287710	2.25445493205147	0.91598217911021
C	2.80047774228792	0.07927308748653	3.32007320451889
C	-0.80900810138282	0.36148126393563	-0.64881287960446
C	1.60429343184418	-2.06653805520903	-2.12518344830145
C	-1.43505982332564	-2.53632721552331	2.16102581299534
C	-1.61100495578848	1.01161418383660	0.31070500933550
C	2.69517372049382	-1.80642211347163	1.61893296052994
C	2.18630427577187	-1.14433109268166	2.70405237301936
H	3.32722526569044	-0.22402173789902	4.23158564605801
H	3.51679756720829	0.55846079868705	2.65492974062707
H	2.05501346662548	0.81901509343392	3.61491478099382
H	3.55894345382620	-1.43031848710431	1.08499279754116
H	2.40651004037144	-2.82441604627236	1.39444917316657
H	-0.41017620621809	2.65731783141164	0.98039089923222
H	-2.04886898202415	2.49397384114283	1.76646536544801
H	-1.30485745992920	-0.42101125311487	-1.20692483928590
H	-0.13721911922676	0.97518190706193	-1.23979813491739
H	1.45752033711636	-1.65662159403754	3.32060249255774
H	-2.51613867151311	0.48205459234127	0.59780978631417
B	2.28468629145902	2.33086906833058	0.55220892340476
F	1.84290613554699	2.99204231419583	1.67699869869682
F	1.73962182261496	2.87071489348496	-0.60805339192548
F	3.63860784565678	2.17220573635798	0.49615303444337
H	-1.73520071314567	3.16876008150862	-0.14242777021241
N	-1.90680032622179	3.96444789706278	-1.13725399209277
C	-3.13999230541013	4.24344499361768	-1.56195891004734
C	-0.82961187981676	4.46514615474528	-1.74947161942864
C	-3.35046153323732	5.06782401143299	-2.65297547667565
C	-0.96686017839789	5.29699346437545	-2.84995140695355
C	-2.24348824792814	5.60202177711918	-3.30531158066950
H	-3.95300006442987	3.78889836793365	-1.00848819036761
H	0.13352253095158	4.17442744864663	-1.34541139148184
H	-4.35714007180134	5.28367430865654	-2.98301250578532
H	-0.08552885698672	5.69014654721797	-3.33722191102380
H	-2.37695585072875	6.24835986475888	-4.16337043339614

TS2G

Mo	0.54199464326821	0.00023658665382	-0.39720804372485
F	4.79185612085673	-0.05491933029709	-3.20870615015107
F	3.85113411327610	0.77730391802452	-1.43989710321892
F	4.77643910789117	-1.16736770733078	-1.34736115212680
F	2.23807204474164	1.08443021292374	-3.61120432566390
F	2.52909828845355	-0.62486468298886	-4.90170957808747
F	0.71329652659075	-0.44136088812979	-3.73042688329236
F	1.81368686509804	-3.00437344234456	-3.49283394810290
F	3.93293938507389	-2.63975350182957	-3.79150824132880
F	3.19771826261459	-3.24521206497299	-1.85100060198990
F	0.81787480781660	-4.31856758264605	-0.92110943444622
F	-0.34887027147593	-5.22469810819023	0.66322595195197
F	1.14253548788886	-3.72376726867771	1.13016102933775
F	-2.79796767630742	-2.44377007188664	-1.23834948210867
F	-0.92829005187344	-2.70475903725549	-2.28835010105413
F	-1.95430754469614	-4.42542856820890	-1.46156630196656
F	-1.20634045360572	-3.19027525524979	2.45146977598368
F	-2.51037291824647	-1.82127769523626	1.41417494829937
F	-2.76648000975907	-3.95740061308825	1.16096145863312
O	-0.21287478526592	-1.73821930726777	0.07848017031153
O	1.85898728373136	-1.00795934716896	-1.43046494957721
O	0.96770338068809	1.57090296027538	-0.99860160417016
C	4.05795004145420	-0.34545142698948	-2.12739579114371
C	2.04083795718839	-0.22820526278325	-3.72230608529555
C	2.92040843667075	-2.50518123028527	-2.92493883389178
C	0.21191780754773	-4.08225532550490	0.24175032497184
C	-1.65025488171260	-3.14067953299647	-1.25645812459919
C	-1.85779791739932	-2.99086099471031	1.29524071326806
C	-1.53524557001412	0.42517472063224	-0.93099056445565
C	1.87876827900471	1.63643004843113	1.80395624209258
C	-3.00017518893086	2.38597839461363	-0.54945389707549
C	2.68725004197900	-1.00724578921938	-2.51585841274143
C	-0.85427757574941	-2.93263312109105	0.08611295236142
C	-1.98051251648653	1.80643532587804	-1.18038972747112
C	1.06602569764366	-0.60855077694700	2.53819539912896
C	0.95541621113736	0.47360926417739	1.47582565019828
H	1.69183283458011	1.96724582880366	2.83107880333468
H	2.92054503811809	1.30757888881323	1.76078230426411
H	1.76385671896727	2.48367165370514	1.13733428311897
H	0.88812349748614	-0.15498735664984	3.51928435202976
H	0.37566907890961	-1.43064678453706	2.40542027017170
H	-1.51095836302156	-0.14676294456284	-1.86746637907991
H	2.08000090493079	-1.01690234559376	2.55861885312481
H	-2.17684565175205	-0.08993899697790	-0.22192125137865
H	-3.30604570521881	3.39611926829465	-0.78857462782479
H	-3.57163400876924	1.86639177044774	0.21238785876536
H	-1.45371381583920	2.36128174787939	-1.94670017131977
B	1.15324149948622	3.12684600937361	-1.42092091492075
F	2.49713035425764	3.33126161918752	-1.41368365250746
F	0.49497878084747	3.80676697469333	-0.40604550223574
F	0.54751243709768	3.25663461124748	-2.63550100830746
H	-0.34903771280777	1.04412600954746	1.63310064185262
N	-1.32415287471405	1.74596019533283	2.25691008896279
C	-1.37235369702383	3.06340495498659	2.05340815278538
C	-2.26282127151993	1.12482289010267	2.97244583390011
C	-2.38628696560922	3.83115818079714	2.60508009404291
C	-3.31237684897315	1.82455830512084	3.54052043425048
C	-3.36846342957635	3.20271978699708	3.35733082300693
H	-0.59933333521582	3.48457641178879	1.42513382285259
H	-2.16077397398488	0.05274906064772	3.07495036591534
H	-2.40635293586677	4.89720765088338	2.42917252978123
H	-4.06446662153603	1.30111150374171	4.11373220269729
H	-4.17541936234468	3.77758160761636	3.79266051386273

2-methylbut-2-ene

C	-0.92237966554576	4.18493177219057	-1.81339778605452
C	-1.52927418537137	1.37122025547593	0.83422100603067
C	-1.36461930809850	3.32501929854732	-0.66982164251700
C	0.03074857833791	1.28808701586004	-1.15204287397497
C	-0.97161339586143	2.08613284216787	-0.36634382620966
H	-0.73081324519608	1.09181527196813	1.52962948009111
H	-2.25057002232833	1.98645461461029	1.37251268446759
H	-2.02480375180542	0.44026365314130	0.53945897101827
H	-0.41553491957663	0.35048043364263	-1.49878844939437
H	0.41661601710686	1.81762682088569	-2.02014644363733
H	-1.77468660194074	4.46576960748845	-2.44007590900755
H	0.88058673480303	1.01182511209938	-0.51955392560959
H	-0.18618060983472	3.70010305769041	-2.45247571770294
H	-0.48347279875057	5.11904081780025	-1.44901281030096
H	-2.09929780293824	3.78500499143172	-0.01295644219873

IM6D

Mo	-3.32339617258119	1.72643038117867	-2.05779099009323
F	-0.04406009828305	-1.89488103428394	-1.73185256136394
F	-0.50470734348737	0.22208843425697	-1.61225310404184
F	-0.95329544743058	-1.07046784220484	0.05417249434412
F	-1.31451079511651	-0.80219434590165	-3.99729582831639
F	-1.96403817701880	-2.83291519568910	-3.66094404397692
F	-3.42617871530770	-1.27486532839908	-4.01555405057075
F	-4.13477366415912	-2.85736150369725	-1.82371614368465
F	-2.20468677091125	-3.53439935434931	-1.09128722485340
F	-3.39457408484871	-2.16746228308529	0.08471360769048
F	-4.08103116201124	0.26635991666257	1.62905923473592
F	-6.05461655579294	0.54242459329992	2.47897722878345
F	-4.66830256658329	2.20861159665156	2.36568105479214
F	-6.58781874536039	0.44740568206806	-1.62671605974328
F	-5.89155804727763	-0.86477187118874	-0.05981778259491
F	-7.71565517016010	0.26747217851933	0.21696049147627
F	-5.78353239723901	3.87386873621181	0.52680079255967
F	-7.26914560639467	2.93063580130699	-0.72167548635279
F	-7.38526629084460	2.73398177232512	1.43417805688724
O	-4.57343786584890	1.75857081238482	-0.50040524671131
O	-3.16887518760863	-0.17473329164829	-1.61243038194821
O	-2.51038742725782	1.64561930034148	-3.53025913958350
C	-0.93762643167977	-1.00895883250128	-1.28210154525697
C	-2.26278401275316	-1.54905752090652	-3.41943648385614
C	-3.03460257989889	-2.48311839548647	-1.15746718740859
C	-5.10604706023606	1.10942242295523	1.72390186322080
C	-6.47888027177285	0.30759593498252	-0.29805899974559
C	-6.54350813511587	2.77405300734381	0.40007840381422
C	-3.14788503404154	5.06915824889675	-3.25366634583559
C	-2.31714804256303	3.74214806686605	0.05188588030301
C	-4.66398393564462	3.08367427746363	-2.89505956915493
C	-2.36987921489668	-1.24995304942659	-1.87352377772044
C	-5.63378530872089	1.49504863456461	0.29497350387224
C	-3.64722686690200	4.05155337324168	-2.23324552926929
C	-0.95087759658369	3.66409531742181	-2.03870964767064
C	-2.29813761284400	3.36347594217514	-1.41734862978306
H	-2.06934871605057	4.80402242017241	0.15653026328084
H	-3.27201756676469	3.55655505285503	0.52970883871637
H	-1.54960951650158	3.17688336153952	0.58318335515268
H	-0.20399776322832	3.03881851167203	-1.54682916528796
H	-0.91344120579756	3.45077564045232	-3.10250494741024
H	-4.00068810475273	5.62906501145188	-3.63815079987519
H	-0.66251766859133	4.70483298560261	-1.86511022801280
H	-2.66043171689418	4.57676418208217	-4.09395374637002
H	-5.65042452861193	3.10622589459712	-2.45692883484863
H	-4.66343989886236	3.12984962316299	-3.97657963905531
H	-2.44873958386586	5.77640285381304	-2.80980333490564
H	-4.15603526090173	4.51505878924843	-1.39521514932708

TS3D

Mo	0.17322174227289	0.13591440151178	-0.83670167149435
F	4.74649176385910	-0.72466832620751	0.73716218597765
F	3.18168352869454	0.69269554564818	0.24060962090347
F	3.13709070845807	-0.33520366973250	2.13552687350757
F	3.62973872975625	-0.94204578457329	-1.86601702819874
F	4.19789191811561	-2.87389842129411	-1.08623678998619
F	2.23729146966264	-2.59597925031762	-1.96287901421265
F	2.04873619365201	-3.93293528775609	0.47647458327745
F	3.79979874206156	-3.20823654807411	1.53832954172191
F	1.81994081983599	-2.66466615349457	2.20974195062872
F	-0.32180388761508	-0.92071489923489	3.02285622773676
F	-2.23970574232446	-1.70126272593453	3.65975554563640
F	-1.99457643836694	0.42028990228298	3.26999202549891
F	-1.88509228608788	-2.67870216861629	-0.35041447636723
F	-0.88728813315732	-3.11753623330502	1.51366312553909
F	-3.04782831857294	-3.19952147149715	1.40460282116402
F	-3.48329879597936	0.86350918758967	1.04241166309119
F	-3.96702965891068	-0.92776888660046	-0.05986833487629
F	-4.33591266144415	-0.83890535734377	2.07416456798406
O	-1.15709054377161	-0.30718643982331	0.58813060870246
O	1.27076841890798	-1.20735688779966	0.08720131182035
O	1.13970863683217	0.25494745258569	-2.20970597147369
C	3.43555561217243	-0.48622569973940	0.84012305105316
C	3.17498808610400	-2.01662107885851	-1.20925810918754
C	2.56502010621228	-2.88235848778275	1.12804556776704
C	-1.63732951706150	-0.81178369498553	2.86115783815304
C	-1.96234984834968	-2.53750186529171	0.98103572992193
C	-3.48480072889338	-0.47735243305741	1.11233279850202
C	-1.26344461436913	2.70993761149997	-2.79652160104579
C	-0.45656452464613	2.67134583782146	0.78096001487536
C	-1.54141775590725	0.31202409599529	-2.06579640247683
C	2.56616650950802	-1.61830591715230	0.19114256182749
C	-2.02381466683775	-1.00947709315472	1.35062032203278
C	-1.37026925475185	1.74062072517353	-1.63129350185279
C	1.09726715309666	3.03219167294965	-1.14856520161819
C	0.08122649374325	2.11306437406918	-0.51844169037507
H	-0.88276033099318	3.66487840255018	0.60746341019009
H	-1.20459862634015	2.03866942676320	1.24409019744287
H	0.37316193821417	2.79352767662104	1.48044286497801
H	1.97198239946411	3.04790152041615	-0.49460742966804
H	1.42467281491239	2.70199475729072	-2.12943447189315
H	-2.19590925614819	2.67058152563191	-3.36152636893091
H	0.72361946440114	4.05783916202022	-1.20894204147575
H	-0.45301820961117	2.42722562992305	-3.46709008464691
H	-2.40798614716948	-0.20046446846550	-1.67728863460593
H	-1.34730977332438	0.12742036907379	-3.11474317975644
H	-1.10916638145561	3.73716619550968	-2.47178109829461
H	-2.13565714784798	2.00293377716542	-0.91092390749672

IM7D

Mo	-3.72828432639804	1.39194809365634	-2.09061472905140
F	-0.69379566276877	-2.03119477084637	-1.02471820811191
F	-1.58609363139599	-0.06907603837015	-0.76631090503385
F	-2.60259669761029	-1.82203655004655	-0.02130121391944
F	-0.79743715977907	-0.25672269284035	-3.34890450570038
F	-0.85649932792987	-2.38537686429960	-3.77456754933357
F	-2.27431579570314	-1.04111247585220	-4.71303842905785
F	-3.74337376116858	-3.08774161238986	-3.74762512765978
F	-2.53881761271213	-3.86137774023657	-2.11742360181935
F	-4.42273708055229	-2.86895187505553	-1.71050376407448
F	-3.73648953583549	1.00930465194692	2.23966193838333
F	-5.68375084707964	1.61082001440036	2.97234100519492
F	-4.29937274050755	3.09334061945815	2.19845236838849
F	-6.30833294684250	-0.01462228449550	-0.79306368995211
F	-5.50888317844204	-0.66924781143996	1.10151530254771
F	-7.36648729809746	0.44584398344495	1.04552887321658
F	-5.48962443055350	3.93294633679986	-0.12586103584609
F	-7.03386585524221	2.59345204110301	-0.82421596195589
F	-7.02763784363537	3.24960799391656	1.23916690871007
O	-4.25442063686152	1.58651842213763	-0.25497739448930
O	-3.57470034522216	-0.49964824026463	-2.45821912190637
O	-2.53456239071999	2.47909698446092	-2.49376224039350
C	-1.84840734485370	-1.37012541387583	-1.02422191703028
C	-1.60914972500145	-1.29961409314106	-3.58619353060936
C	-3.34327262362723	-2.85877189944066	-2.49235008460003
C	-4.75434488739518	1.84522088648313	2.03827404296248
C	-6.15480941417930	0.33100927052783	0.49947649309139
C	-6.24293231723285	2.88851115560854	0.22054409295695
C	-5.14710197014169	1.93745609802517	-3.18396550370899
C	-2.61197189078907	-1.47426584956767	-2.39228263009838
C	-5.33223677401708	1.66254806931649	0.58914635009826
H	-6.04239731447051	1.31562960248380	-3.15103837122954
H	-5.18203658364387	2.80610628232471	-3.83098481812989

TS8D

Mo	0.06928603028388	0.19176746074334	-0.95675221478698
F	2.93769535773688	-3.22396352853472	0.43573175808001
F	2.08040882802261	-1.23027546941346	0.55340396001991
F	1.12159750332202	-2.88903114107716	1.55962585693720
F	2.80506585484207	-1.85946848624405	-1.99917210809137
F	2.45362376251774	-3.97538805382539	-2.25981525894679
F	1.13417486636673	-2.53730398893998	-3.19785853063953
F	-0.31183737859712	-4.50985927920251	-1.93897881258408
F	0.98408719585600	-5.13276426933989	-0.30998002981116
F	-0.86454955651616	-4.09066540388612	0.10309157045813
F	-0.09560611159771	-0.77675153650285	3.06966450117582
F	-2.01555814489394	-0.59878825880987	4.05992212883543
F	-0.86816022895263	1.17280679447232	3.58409475862527
F	-2.96517895253773	-1.41670950909314	0.15986077535699
F	-1.97957824547866	-2.35289783411127	1.84215247800858
F	-3.86351928013454	-1.32360724687000	2.12716964881522
F	-2.23211670240783	2.40874691393436	1.60253847016963
F	-3.71450685331332	1.16746101077428	0.64624452356034
F	-3.65625012721188	1.31345856382084	2.80952703877568

O	-0.96578779864608	0.21199916843646	0.76517461500670
O	-0.03877801286289	-1.79871425844918	-0.89671922272046
O	1.05037892054674	0.26473347469541	-2.32112903806049
C	1.78300344137720	-2.55878469857097	0.45298766255646
C	1.84271667757609	-2.79217410257989	-2.10037571775773
C	0.16397698508509	-4.15812873728188	-0.73674442551618
C	-1.21118449590675	-0.05090842826980	3.14181544082817
C	-2.69770943724099	-1.29473289791650	1.46262938176404
C	-2.89916198077697	1.24678853501576	1.71349851592632
C	0.24984544031828	3.46148714805549	-2.16811663581094
C	1.21199312679572	2.11653377996935	1.29926559359504
C	-1.37611318505269	1.13103564437618	-1.81216198106678
C	0.90599344248649	-2.77523474799027	-0.82866425566747
C	-1.89694056035216	0.03657956421417	1.72788658159772
C	0.08190737194847	2.69067674150654	-0.88584550527152
C	2.56393362071819	2.14307430107482	-0.82489828999315
C	1.17582559693159	2.08853726059865	-0.21734546904562
H	1.84227024048837	2.95805591837241	1.60388495182924
H	0.23128441174676	2.25218193605415	1.73596227555491
H	1.66002708415729	1.21470853122192	1.71695902413913
H	3.19776321109343	1.36074494401353	-0.40665379734512
H	2.56795604496031	2.03747137876390	-1.90462451509778
H	-0.70839106629833	3.78547660316332	-2.56972193528924
H	3.02030171562308	3.10387523170892	-0.56435329312853
H	0.77174955357667	2.88499751768637	-2.92958179605779
H	-2.30680426693462	1.30267748365610	-1.28360442936054
H	-1.35123787847622	1.44271181379163	-2.84801387200379
H	0.84797346452733	4.35471884790242	-1.96226948039785
H	-0.73586948471583	3.00284530888624	-0.24971089716505

References

1. T. Saito, H. Nishiyama, H. Tanahashi, K. Kawakita, H. Tsurugi and K. Mashima, *J. Am. Chem. Soc.*, 2014, **136**, 5161–5170.
2. B. Kamenar and B. Korparcolig, *Inorg. Chim. Acta*, 1982, **65**, L245–L247.
3. Berkson, Z. J.; Zhu, R.; Ehinger, C.; Lätsch, L.; Schmid, S. P.; Nater, D.; Pollitt, S.; Safonova, O. V.; Björgvinsdóttir, S.; Barnes, A. B.; Román-Leshkov, Y.; Price, G. A.; Sunley, G. J.; Copéret, C. *J. Am. Chem. Soc.* 2023, **145**, 12651–12662.
4. P. A. Zhizhko, V. Mougel, J. de Jesus Silva and C. Copéret, *Helv. Chim. Acta*, 2018, **101**, 2–7.
5. F. Neese, *WIREs Comput. Mol. Sci.*, 2012, **2**, 73–78.
6. F. Neese, *WIREs Comput. Mol. Sci.*, 2025, **15**, e70019.
7. A. D. Becke, *J. Chem. Phys.*, 1993, **98**, 5648–5652.
8. C. Lee, W. Yang and R. G. Parr, *Phys. Rev. B*, 1988, **37**, 785–789.
9. S. Grimme, J. Antony, S. Ehrlich and H. Krieg, *J. Chem. Phys.*, 2010, **132**, 154104.
10. S. Grimme, S. Ehrlich and L. Goerigk, *J. Comput. Chem.*, 2011, **32**, 1456–1465.
11. F. Weigend and R. Ahlrichs, *Phys. Chem. Chem. Phys.*, 2005, **7**, 3297–3305.
12. F. Weigend, *Phys. Chem. Chem. Phys.*, 2006, **8**, 1057–1065.
